# Deep learning framework for epidemiological forecasting: A study on COVID-19 cases and deaths in the Amazon state of Pará, Brazil

**DOI:** 10.1371/journal.pone.0291138

**Published:** 2023-11-17

**Authors:** Gilberto Nerino de Souza, Alícia Graziella Balbino Mendes, Joaquim dos Santos Costa, Mikeias dos Santos Oliveira, Paulo Victor Cunha Lima, Vitor Nunes de Moraes, David Costa Correia Silva, Jonas Elias Castro da Rocha, Marcel do Nascimento Botelho, Fabricio Almeida Araujo, Rafael da Silva Fernandes, Daniel Leal Souza, Marcus de Barros Braga

**Affiliations:** 1 Universidade Federal Rural da Amazônia, Paragominas Campus, Paragominas, Pará, Brazil; 2 Universidade Federal Rural da Amazônia, Capitão Poço Campus, Parauapebas, Pará, Brazil; 3 Universidade Federal Rural da Amazônia, Parauapebas Campus, Parauapebas, Pará, Brazil; 4 Cyberspace Institute, Universidade Federal Rural da Amazônia, Belém, Pará, Brazil; 5 Socio-Environmental Institute of Water Resources, Universidade Federal Rural da Amazônia, Belém, Pará, Brazil; 6 Computer Science Department, Universidade da Amazônia, Belém, Pará, Brazil; 7 Computer Science Institute, Centro Universitário do Estado do Pará, Belém, Pará, Brazil; Jeonbuk National University, REPUBLIC OF KOREA

## Abstract

Modeling time series has been a particularly challenging aspect due to the need for constant adjustments in a rapidly changing environment, data uncertainty, dependencies between variables, volatile fluctuations, and the need to identify ideal hyperparameters. The present study presents a Framework capable of making projections from time series related to cases and deaths by COVID-19 in the Amazonian state of Pará, in Brazil. For the first time, deep learning models such as TCN, TRANSFORMER, TFT, N-BEATS, and N-HiTS were assessed for this purpose. The ARIMA statistical model was also used in post-processing for residual adjustment and short-term smoothing of the generated forecasts. The Framework generates probabilistic forecasts, with multivariate support, considering the following variables: daily cases per day of the first symptom, cases published daily, the occurrence of deaths, deaths published daily, and percentage of daily vaccination. The generated predictions are statistically evaluated by determining the best model for 7-day moving average projections using evaluating metrics such as MSE, RMSE, MAPE, sMAPE, r^2^, Coefficient of Variation, and residual analysis. As a result, the generated projections showed an average error of 5.4% for Cases Publication, 8.0% for Cases Symptoms, 11.12% for Deaths Publication, and 4.6% for Deaths Occurrence, with the N-HiTS and N-BEATS models obtaining better results. In general terms, the use of deep learning models to predict cases and deaths from COVID-19 has proven to be a valuable practice for analyzing the spread of the virus, which allows health managers to better understand and respond to this kind of pandemic outbreak.

## Introduction

Epidemiological predictions have become an important role in combating disease outbreaks around the world and various artificial intelligence models, such as machine and deep learning, have proven useful and have been widely used during these pandemics, including COVID-19, especially when it comes to medical diagnosis, development of new drugs, discovery of vaccine targets, tracking and monitoring of the disease and time series analysis of the incidence of the epidemic [1, 2]. However, the task of forecasting and analyzing time series has shown that there are great challenges, due to factors such as the lack of available and curated large-scale training data, distributed and heterogeneous nature of many data sources, and the need for interdisciplinary cooperation of different knowledge (such as computer science, mathematics, statistics, biology, virology, pharmaceuticals, and medicine) [3, 4].

In this sense, several studies have indicated that deep learning models, in addition to their own methodological qualities, such as the data-oriented approach, which does not require specialized knowledge, also have significant accuracy when compared to other mathematical and statistical models [5, 6]. The extensive use of deep machine learning applications to predict cases, deaths, incidence, transmission, and hospital resources has been applied in several countries like India [7], Saudi Arabia [8], USA [9], Brazil [10–12], Egypt [13], Malaysia [14], and several countries in Europe [15], highlighting the importance of local application. Particularly, deep learning algorithms based on recurrent neural models, such as RNN, LSTM, and GRU, have been widely used, generally generating deterministic projections [16–18].

In terms of time series modeling, there are several goals, challenges, and indications in the scientific literature to achieve the objective of forecasting with accuracy and reliability, especially feasible for decision-makers. Among the most critical challenges is the existence of a high degree of uncertainty in the data, indicating that probabilistic predictions can be better used to project cases and deaths [19]. Another factor to be considered, in the case of COVID-19, is the dependency relationship between the various variables associated with the disease, where one variable influences the values of another variable. For example, the number of daily deaths has a high correlation with the percentage of vaccinated [20, 21]. However, most of the deep learning models applied to the pandemic context do not take into account the recent vaccination campaigns that took place around the world and that favorably change the dynamics of the pandemic [22]. There is also the challenge of predicting the number of new cases given the volatile fluctuations in daily numbers, which may indicate adjusting the longer time period input to build models with reasonable predictions [23]. Another example is the number of published cases, which is associated with variables related to the month of the year, due to seasonality, weather periods, and even year-end and mid-year holidays [24]. Furthermore, the large number of deep learning models and the identification of their optimal hyperparameters represent a significant additional difficulty [25].

The present study presents a Framework for forecasting time series related to COVID-19 cases and deaths in the state of Pará, eastern Brazilian Amazon. The proposed Framework uses the following deep learning models for time series: TRANSFORMER, TCN, TFT, N-BEATS, and N-HiTS; which, as far as we know, have not been jointly addressed in the literature. Additionally, the ARIMA method is used as a post-processing method for the diagnosis of residuals and smooth adjustment of the generated projections, thereby enhancing the suggested framework.

The methods applied in this research generate probabilistic forecasts and support multivariable and auxiliary variables, such as vaccination variables. Consequently, this approach is statistically evaluated and selects the best model for a 7-day short-term projection, which helps combat pandemics such as COVID-19 and for researchers, managers, and decision-makers in the public and private spheres.

In Section 2 (Materials and Methods), we demonstrate the execution of the proposed framework, in addition to presenting the study area, data, techniques, and models used, exemplifying experiments with the developed procedures. In Section 3 (Results and Discussion), we show the data generated after experimenting with the proposed approach through graphs, tables, and information relevant to the study area, where we also comment on the results and limitations of performing an analysis on some experiments of the Framework. Finally, in the final Section, Conclusion, we make some final considerations and indicate future work for this research.

## Materials and methods

**Fig 1:**
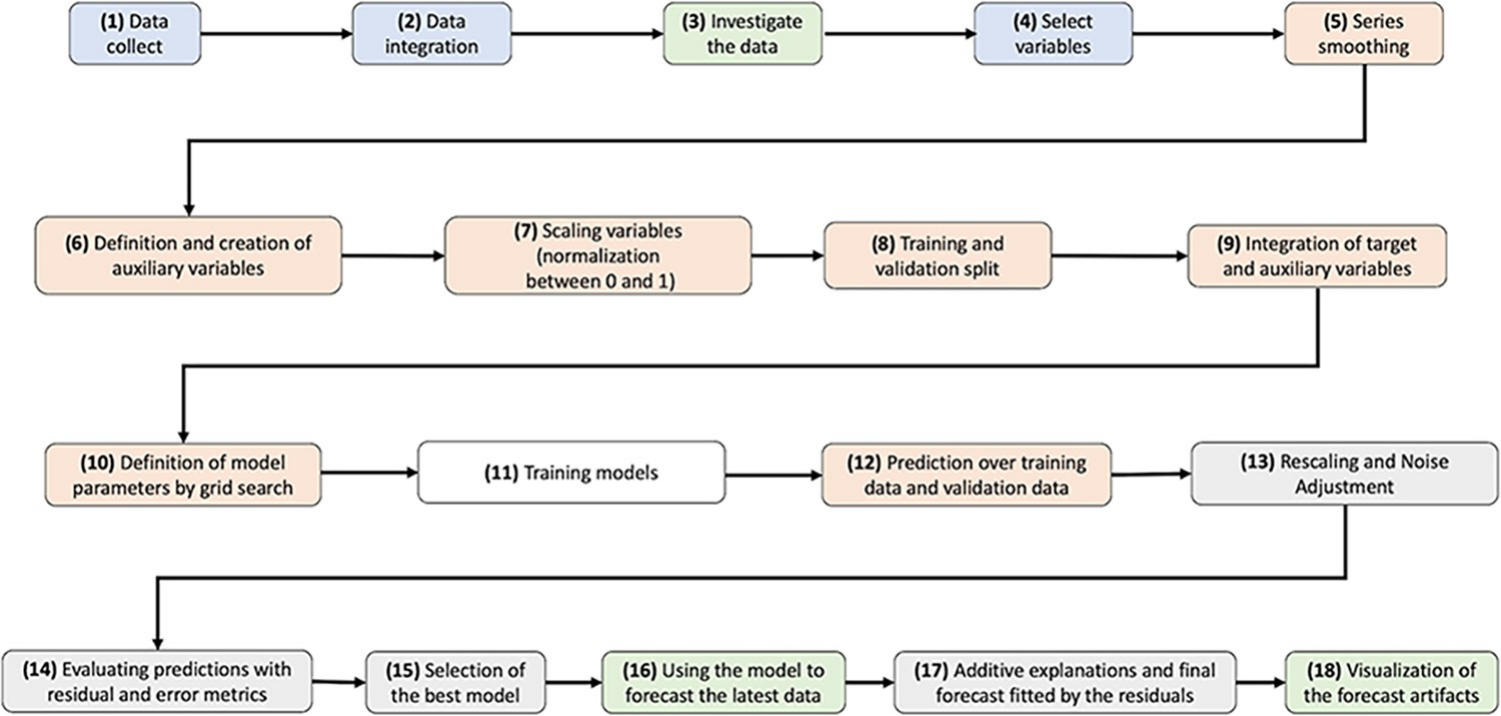
Framework pipeline.

This study proposes a Framework exemplified in the Fig 1 pipeline that can be executed in a timely manner (less than 24 hours), through deep learning techniques, for forecasting growth trends, stabilization or reduction of cases and deaths, in the context of a pandemic, in the state of Pará, Brazil.

The Framework pipeline is categorized with colors to facilitate the understanding of the steps in terms of upcoming procedures in the data mining area, namely: blue, data acquisition and integration procedures; green, information visualization procedures; orange, procedures for handling and processing data; white, model training; gray, post-processing, predictions and evaluation of the models. The procedures are briefly listed in Table 1.

**Table 1:** Procedures listed in order of execution.

Procedures Name		Procedures Description
**1.**	**Data collection**	Collecting data based on various data sources
**2.**	**Data integration**	Integrating various data sources into a single table forming the dataset for machine learning and data mining
**3.**	**Data investigation**	Generating descriptive data and previewing time series data;
**4.**	**Variables selection**	Selecting target data based on preview data and forecast objective
**5.**	**Series smoothing**	Smoothing of target series to reduce noise in the data
**6.**	**Auxiliary variables definition and creation**	Selecting and creating auxiliary series that have not been predicted but are relevant for predicting the target series
**7.**	**Scaling variables**	Normalization of all series between 0 and 1
**8.**	**Training and validation split**	Division of preprocessed dataset between training dataset and validation dataset
**9.**	**Integration of target and auxiliary variables**	Integrating target series and auxiliary series into a single data structure for model training
**10.**	**Definition of model parameters by GridSearch**	Defining parameter values and ranges used for each model to be used in the GridSearch
**11.**	**Train models**	Executing GridSearch that already performs training with the best hyperparameters of each model
**12.**	**Prediction over training data and validation data**	For each model trained by GridSearch, several predictions are made on the historical data (training data) and data ahead of the last day of the training data (validation data) mainly selecting the median of these projections for further analysis
**13.**	**Rescaling and noise adjustment**	Scale the predicted data to the original scale by adjusting inconsistencies (eg: deaths with values negatives)
**14.**	**Evaluating predictions with residual and error metric**	Calculate errors and residuals on the training and validation data for model evaluation
**15.**	**Selection of the best model**	Select the model for each series after the training and validation step based on errors and residuals
**16.**	**Using the model to forecast the latest data**	The best model is used to project *n* days ahead of the forecast horizon and on the observed data
**17.**	**Additive explanations and final forecasts fitted by the residuals**	The observed and forecasted data are passed on as input for a post-processing and prediction smoothing stage, through the ARIMA method, which acts on the residuals, trend, and seasonality of the data
**18.**	**Visualization of the forecast artifacts**	Graphs are generated with the projections of the time series, with upper and lower limits being smoothed by splines, in parallel, complementary graphs are generated on the residuals of the projections and errors of the projections on observed data

The pipeline follows an adapted data mining process, with the steps of data collection, integration and investigation, selection of variables, series smoothing, division between training and validation, definition of model parameters (Grid Search), training the models, predicting and validating the data, post-processing, selecting of the best model, the use of the model, and finally generating visualizations of the produced data. In this sense, the framework involves various deep-learning models (TRANSFORMER, TCN, TFT, N-BEATS, N-HiTS) and one statistical model (ARIMA) technique to create accurate time series forecasts.

Thus, in this study, the pipeline is executed twice, being similar to two flows of analysis and generation of projections, one for Cases of COVID-19, and another for Deaths caused by COVID-19.

### Study area, data collection, and integration

Pará is the second largest state in Brazil in territorial size, with 1,248,000 km^2^, with more than 8 million inhabitants, and has the lowest municipal human development index (0.698) among the states in the North region of Brazil [26]. These characteristics show the difficulties in the states of northern Brazil, which leads to the need to anticipate possible epidemic increases, especially in the variables related to cases and deaths of COVID-19.

The variables used in this study are daily series containing the notified number of COVID-19 cases and deaths and vaccinations applied in the state of Pará. Data are aggregated daily from official sources, available on the official website of the State Department of Health of the Government of Pará [27], together through a repository on GitHub COVID19BR [28]. The latter brings together data from the most diverse sources such as the Ministry of Health of the federal government of Brazil, State Health Secretariats, Brazil.IO (a repository of public data in Brazil), and the Brazilian Institute of Geography and Statistics (IBGE), with the numbers updated daily, providing a reliable resource for researchers.

These data are integrated forming a daily dataset of time series with a 7-day moving average (except for vaccination variables that have a percentage scale), with their names in the header of the dataset listed in Table 2 below.

**Table 2:** Variables and their names in dataset header.

Variables	Variables Description
**Cases_DateSymptom_MM_actual_PA (or Cases Symptoms)**	This series represents the Moving Average of the current day (most recent day) of the number of COVID-19 cases, identified by the date of onset of the first symptoms (target series).
**Cases_Publication_MM_actual_PA (or Cases Publication)**	This series represents the Moving Average of the current day (most recent day) of publication of the number of COVID-19 cases (target series).
**Cases_DateSymptom_MM_7days_PA**	This series represents the Moving Average of the number of COVID-19 cases, with data collected 7 days before the current day, identified by the date of onset of the first symptoms (used only for projection).
**Cases_Publication_MM_7days_PA**	This series represents the Moving Average of the number of COVID-19 cases, referring to the date of publication, with data collected 7 days before the current day (used only for projection).
**Cases_DataSymptom_MM_14days_PA**	This series represents the Moving Average of the number of COVID-19 cases, with data collected 14 days before the current day, identified by the date of onset of the first symptoms (used only for model training).
**Cases_Publication_MM_14days_PA**	This series represents the Moving Average of the number of COVID-19 cases, referring to the publication date, with data collected 14 days before the current day (used only for model training).
**Deaths_DateOccurrence_MM_actual_PA (or Deaths Occurrence)**	This series represents the Moving Average of the number of deaths caused by COVID-19, on the current day (most recent day), identified by the day the death occurred (target series).
**Deaths_Publication_MM_actual_PA (or Deaths Publication)**	This series represents the Moving Average of the number of deaths published on the current day (most recent day), caused by COVID-19 (target series).
**Deaths_DateOccurrence_MM_7days_PA**	This series represents the Moving Average of the number of deaths caused by COVID-19, collected 7 days before the current day, identified by the day the death occurred (used only for the projection).
**Deaths_Publication_MM_7days_PA**	This series represents the Moving Average referring to the publication, collected 7 days before the current day, of the number of deaths from COVID-19, identified by the date of the first publication (used only for the projection).
**Deaths_DateOccurrence_MM_14days_PA**	This series represents the Moving Average of the number of deaths caused by COVID-19, collected 14 days before the current day, identified by the day the death occurred (used only for model training).
**Deaths_Publication_MM_14days_PA**	This series represents the Moving Average referring to the publication, collected 14 days before the current day, of the number of deaths from COVID-19, identified by the date of the first publication (used only for training the models).
**Vaccination_dose1_%decimal_PA, Vaccination_dose2_%decimal_PA and Vaccination_dose3_%decimal _PA**	These series correspond to the percentage of the first, second and third dose of the COVID-19 vaccine, respectively, in the federative unit of Pará, already normalized (0 = 0% and 1 = 100%).

In this research, the time series related to cases and vaccination were used for the case projection flow. All series (i.e., cases, deaths, and vaccination) were used, as it is also considered that the variables of cases and vaccination interfere with the number of deaths [20]. Therefore, the target time series of this study are the Moving Average of Cases by date of onset of Symptoms (Symptom Cases), Moving Average of Cases on the day of Publication (Publication Cases), Moving Average of Deaths by date of death (Deaths Occurrence) and Moving Average of Deaths on the day of Publication (Publication Deaths). Respectively: **Cases_DateSymptom_MM_actual_PA, Cases_Publication_MM_actual_PA, Deaths_DateOccurrence_MM_actual_PA, Deaths_DateOccurrence_MM_actual_PA.** The other series (e.g.: vaccination series) are auxiliary variables for the predictive models. It is necessary to highlight that the target variables of cases are predicted together in the case projection flow. As in the flow of deaths, the target variables of deaths are also projected together, due to their interdependencies multivariable.

In this study, we present the individualized results of the execution of the framework with series data from 01/03/2020 to 30/10/2022. Subsequently, a stability analysis is carried out based on the execution of three experiments of the proposed framework.

### Preprocessing

The proposed framework was programmed in Python language with the aid of the DARTS library [29], with all scripts shared on GitHub e Jupyter Notebook (https://github.com/NPCA-TEAM/COVID-19).

In the preprocessing phase, empty/missing values were replaced by zero. After, the target series undergo a Kalman-type filtering to smooth the series and reduce the noise and outliers of the variables [30]. Subsequently, the series of cases and deaths (auxiliaries and targets) are scaled (normalized) between 0 and 1, necessary as input to the neural models.

**Fig 2:**
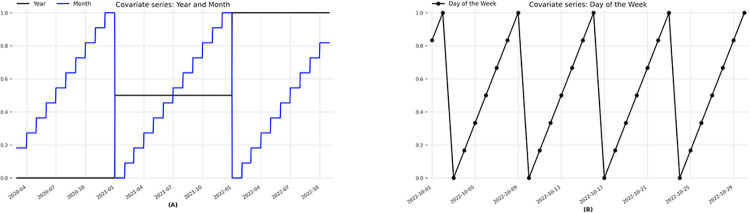
Sample of auxiliary series referring to the year, month (A), and week (B).

In preprocessing, auxiliary series related to the date are also generated, namely: **Year**, **Month, and Week**, as shown in Fig 2.

Thus, the models proposed for use are trained with several time series, supporting covariates (whose past values are known at the time of forecasting) generating probabilistic forecasts as the output of the models.

### Model training

**Fig 3:**
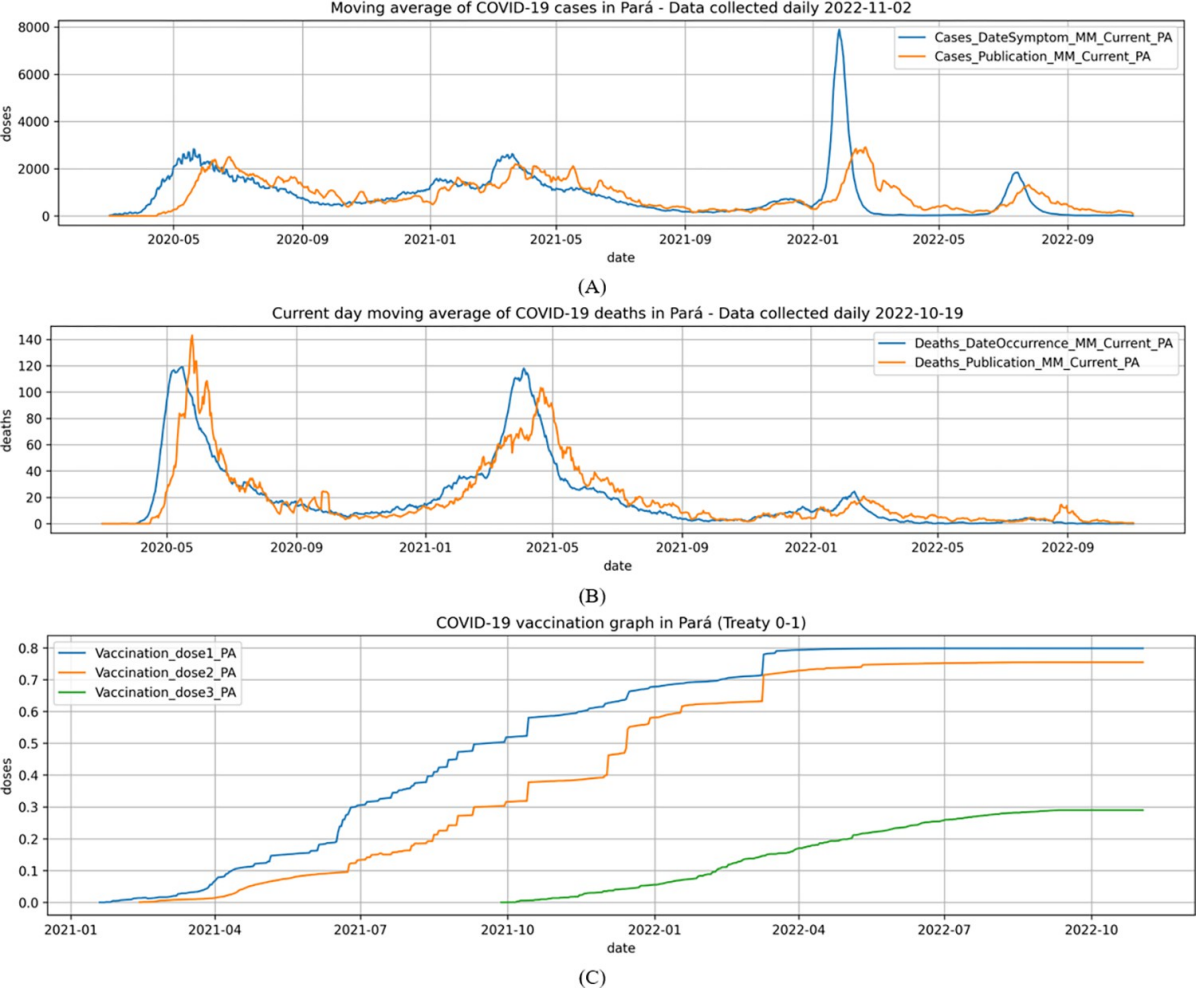
Moving average (7 days) of target series of cases (A), target series of deaths (B), and auxiliary series of percentage vaccination (C).

The dataset (Fig 3), is divided into TrainingDataSet and ValidationDataSet, with the validation dataset having the data of the last 7 days, and the training dataset with the data of the rest of the series. In this sense, the search pipeline forecasts 7 days ahead.

The architecture of the global forecasting models used in this study is based on “blocks”, which are chunks of time series used as input and emit chunks of output (predicted) time series values. In this regard, the size of the input piece is equal to 30 days of the series, working as a sliding window with a daily step (stride = 28) in the process of training the neural models.

To help adjust the hyperparameters of the models, GridSearch was used, which evaluates all possible combinations of hyperparameters, oriented per the well-known scheme of time series cross-validation, returning the model with the best performance in relation to the mean of the RMSE metric function (Root Mean Square Error).

Among the methods/parameters, common to all models and inserted in GridSearch, the following were used: the early stop, which checks at the end of each epoch if the loss function is no longer decreasing, helping to minimize overfitting; Univariate Gaussian probability distribution and quantile regression with quantiles from 0.05 to 0.95 with step 0.05 [31, 32], to enable probabilistic predictions of the models; and, number of epochs equal to 30 for all models. The other parameters of each model, defined after several tests, are presented in Table 3.

**Table 3:** Description of the hyperparameters of the models.

Models	Parameters	Description	Parameters values
For all models	forecast_horizon	Days of forecast	7 days
	start_day	Training starts from one tenth of the time series, excluding that first part	0.05 * length (time_serie)
	input_chunk_length	The length of the input sequence which is applied to the model	30 days
	output_chunk_length	The length of the output sequence (forecast) which is applied to the model	7 days
	stride	Number of time steps between two consecutive training sets	28 days
	n_epochs	Max number of epochs	[30]
	batch_size	An amount of training data (input and output) used at each epoch	[38]
	force_reset	Any previously existing model with the same name will be reset	[True]
	likelihood	Probabilistic distribution used in forecasts models	[Gaussian, QuantileRegression (0.05, 0.1, 0.15,…,0.95)]
	dropout	Probability ratio used to select a certain amount of non-fully connected topologies	[0.05, 0.1]
	metric_validation	A function that takes two series as inputs (actual and prediction) and returns an error value.	RMSE—Root Mean Squared Error
	early_stopping	Training process will stop earlier if the "train_loss" parameters does not improve	Yes, in train_loss with patience = 5.
TCN	kernel_size	Kernel size in a convolutional layer	[3,4]
	num_layers	The number of convolutional layers	[None,1,2]
	num_filters	The number of filters in a convolutional layer	[2,3,4]
	dilation_base	A dilatation factor applied to the convolution process	[2]
	weight_norm	If weight normalization is used	[True, False]
Transformer	d_model	The number of expected features in the transformer encoder/decoder inputs	[32]
	nhead	Number of layers function (heads) of the attention mechanism running in parallel	[2,4]
	num_encoder_layers	Number of encode layers applied to the input data processing	[2,3,4]
	num_decoder_layers	Number of decode layers, used to generate the output	[2,3,4]
	dim_feedforward	The feature number of hidden layer of the Feedfoward Network (FFN)	[256]
	activation	The activation function of encoder/decoder intermediate layer	[’relu’,’gelu’]
TFT	hidden_size	Hidden state size	[8]
	lstm_layers	Number of layers for the LSTM encoder and decoder	[1,2,3]
	num_attention_heads	Number of attention layers running in parallel. The attention module splits its input parameters (Query, Key, and Value) and passes each chunk independently through a separate head	[3,4,5]
	full_attention	If true, it applies multi-head attention query on past (encoder) and future (decoder) parts. Otherwise, only queries on future part	[True]
	hidden_continuous_size	Number of hidden nodes for continuous variables	[8]
	add_relative_index	The TFT model need at least one future covariate to works (ie, known variables into the future, e.g., weather forecasts, days of the week. . .). If True, allow to use the model without having to pass a future covariate, giving a value of crescent index to each step from input and output chunk relative to the prediction point	[True]
NBEATS and NHITS	generic_architecture^1^	If false a non-generic architecture is used, consisting in the utilization one trend stack and one seasonality stack	[True]
	num_stacks	The number of stacks that make up the entire model	[3]
	num_layers	The number of fully connected layers before of the final layers in each block and of each stack	[2,3]
	num_blocks	The number of blocks making up each stack	[1,2]
	layer_widths	The number of neurons that make up each fully connected layer in each block of every stack	[128]
	activation	The activation function of encoder/decoder intermediate layer	[’ReLU’,
			’Tanh’,
			’Sigmoid’]

### Network models

The models used in the training and validation process were based on deep learning architectures and recurrent neural network schemes: Temporal Convolutional Network (TCN); Transformer Network; Temporal Fusion Transformer (TFT); Neural Basis Expansion Analysis Time Series (N-BEATS); Neural Hierarchical Interpolation for Time Series (N-HiTS). All these are recently developed architectures, with applications in the most diverse areas of knowledge (e.g., natural language processing, video segmentation).

#### TCN (Temporal Convolutional Network)

Models derived from convolutional architectures have been applied and improved since the 1980s, with many of these solutions applied in speech recognition systems [33]. The temporal convolutional networks (TCN) model shares the aforementioned architecture, in addition to its characteristic of using the fully convolutional networks (FCN) architecture, with a one-dimensional structure, proposed by Jonathan Long in 2015 [34], where each hidden layer has the same size as the output layer, ensured by the architecture itself. In summary, the TCN model combines the architectures of the one-dimensional FCN model with casual convolutions [35].

In general, TCN networks have three important characteristics [34]:

- The matrix circumvolution process is causal and with defined intervals. In this scenario, there is no leakage of information from the future to the past in time series.
- It produces an output of the same length as the input, as in recurrent networks.
- TCN-based models provide a unified approach to hierarchically capture all two levels of information: spatiotemporal (convolutional networks) and temporal (recurrent networks).

Based on the characteristics described, the study carried out by [33] lists some advantages of using the model, with emphasis on stable gradients, low memory requirements for training, and variable-size inputs. Regarding the listed disadvantages, the need for greater storage for the evaluation due to the raw sequence, in addition to the fact that the TCN model does not have a sufficiently large receptive field, which may lead to low performance.

**Fig 4:**
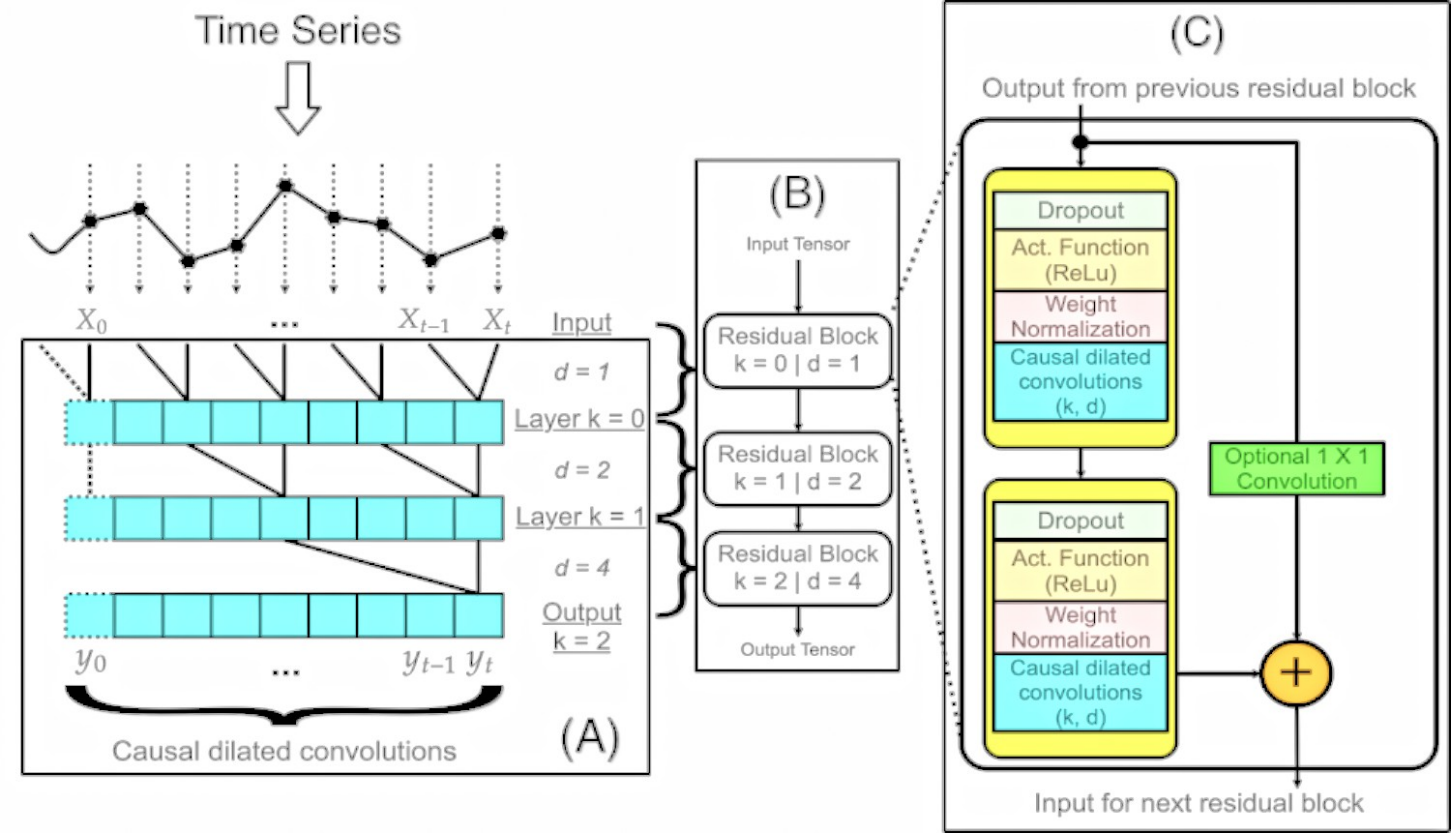
TCN model architecture [32]. For the example above, the causal convolution (A) is set with dilation factors d = 1, 2, 4. By this configuration, the receptive field can cover all values from the input sequence. A series of residual blocks connected in a unidirectional queue scheme are responsible for tensor processing (B). Also, a TCN residual block, which includes activation, normalization, and regularization procedures (C). 1 x 1 convolution is added whether the residual input and output have different dimensions.

In the TCN model, residual blocks are inserted instead of conventional convolutional layers, found in other architectures (e.g., CNN—Convolutional Neural Network). Therefore, each layer integrates a residual block formed by two dilated causal convolutional layers, with dilation factor *d*. For both layers, the ReLU activation function is used, normalization in the filter weights and dropout for regularization. To ensure that the elementary addition takes the tensors properly, an additional 1 X 1 convolution is built into the model in the residual blocks as an optional feature. Fig 4 depicts the TCN model based on the model by Bai et al [33].

#### Transformer Network

Transformer is a deep learning model introduced in 2017, which receives its name because it is structured around an attention mechanism that ensures its high efficiency in “sequence-to-sequence” tasks, that is, those that receive a sequence and produce another. This feature makes it a viable solution for tasks such as translating between languages or subtitling an image. The model learns from a graph passing information between its inputs. Because they don’t parse their input sequentially, Transformers largely solve the problem of gradient runaway, present in recurrent neural networks in long-term forecasting. For this reason, Transformers can be applied to a dataset with long historical information [36].

**Fig 5:**
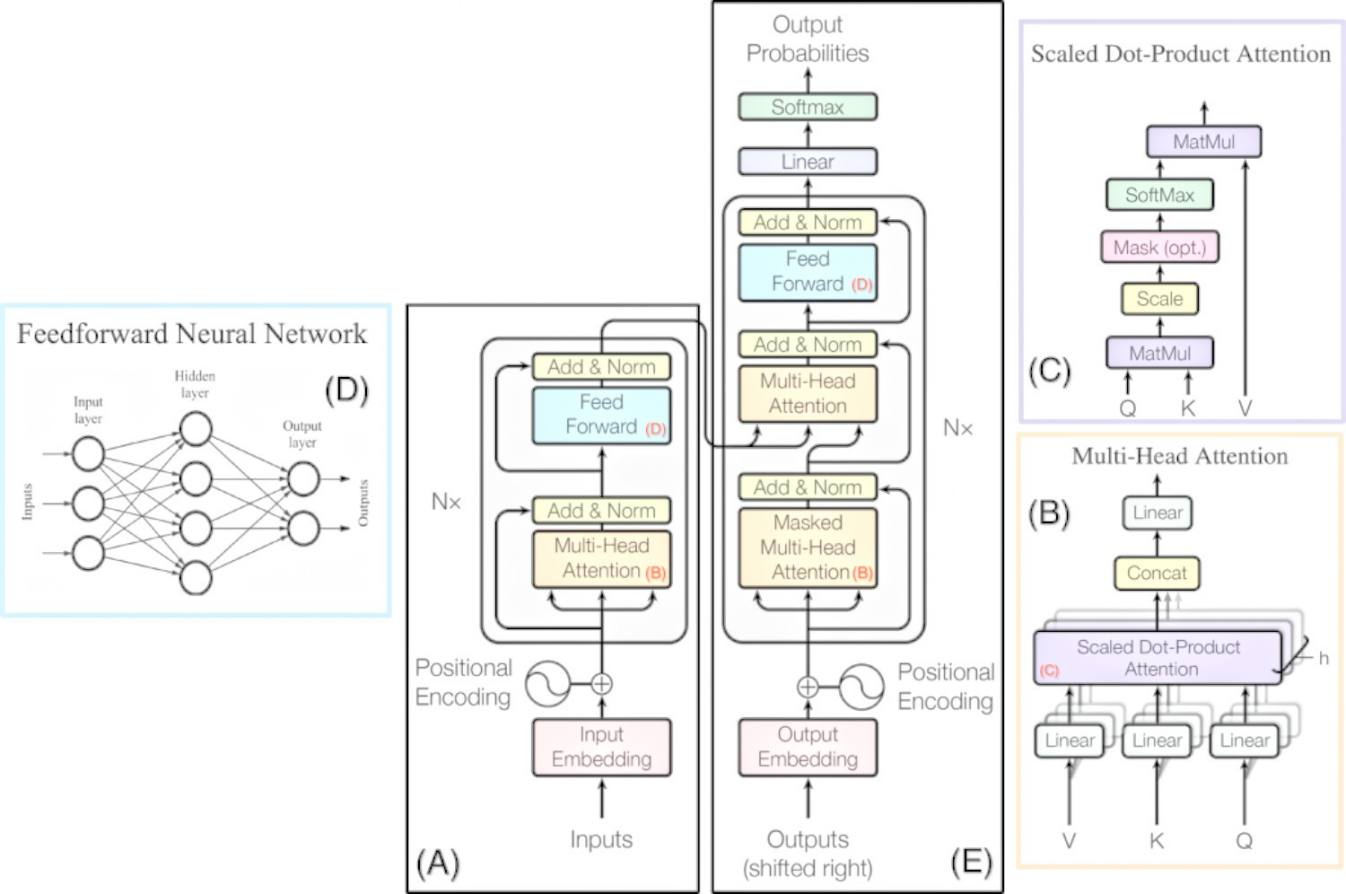
Transformer Network model architecture [35]. The main components are described as follows [34, 35]: Encoder block (A), a set of mechanisms organized in a stack of N identical layers, each of them containing two sub-layers. These are constituted of a series of multi-head self-attention mechanisms (B) with scale-dot product attention (C), as well as a simple, position-wise fully connected feed-forward neural network (D). Like the encoder, the decoder block (E) is also composed of a stack of N identical layers. In addition to the two sub-layers in each encoder layer, the decoder inserts a third sub-layer, which performs multi-head attention over the output of the encoder stack.

The model has an encoder-decoder architecture whose main feature is the Multihead-Self-Attention (MSA) mechanism in which each token along the input sequence is compared to all other tokens to collect information and learn dynamic contextual information [37]. Each encoder is made up of two units: one called “Self-Attention”, which applies the attention mechanism to the data it receives, and another of the feed-forward type, which converts the result of the Self-Attention layer into a structure of data of fixed size and smaller length, called embedding (a sample of data). The Decoder is made up of these same units, but between them, there is an additional layer called “Encoder-Decoder Attention”, which maps the result of the Self-Attention layer of the Decoder with the embeddings produced by the Encoder. Thus, the model can identify interdependencies between the input and output vectors (“Self-Attention”), as well as interdependencies between the same vectors (“Encoder-Decoder Attention”) [38]. Fig 5 illustrates the architecture of a Transformer network.

#### TFT (Temporal Fusion Transformer)

TFT is an attention-based deep neural network optimized for performance and interpretability. The temporal fusion decoder consists of combining specialized layers to learn the relationships along the temporal axis [39]. The model has some advantages, notably the support for temporal data with inputs known in the future and inputs known up to now. In addition, the TFT model uses exogenous categorical variables (also known as time invariants), as well as support for multi-step predictions.

**Fig 6:**
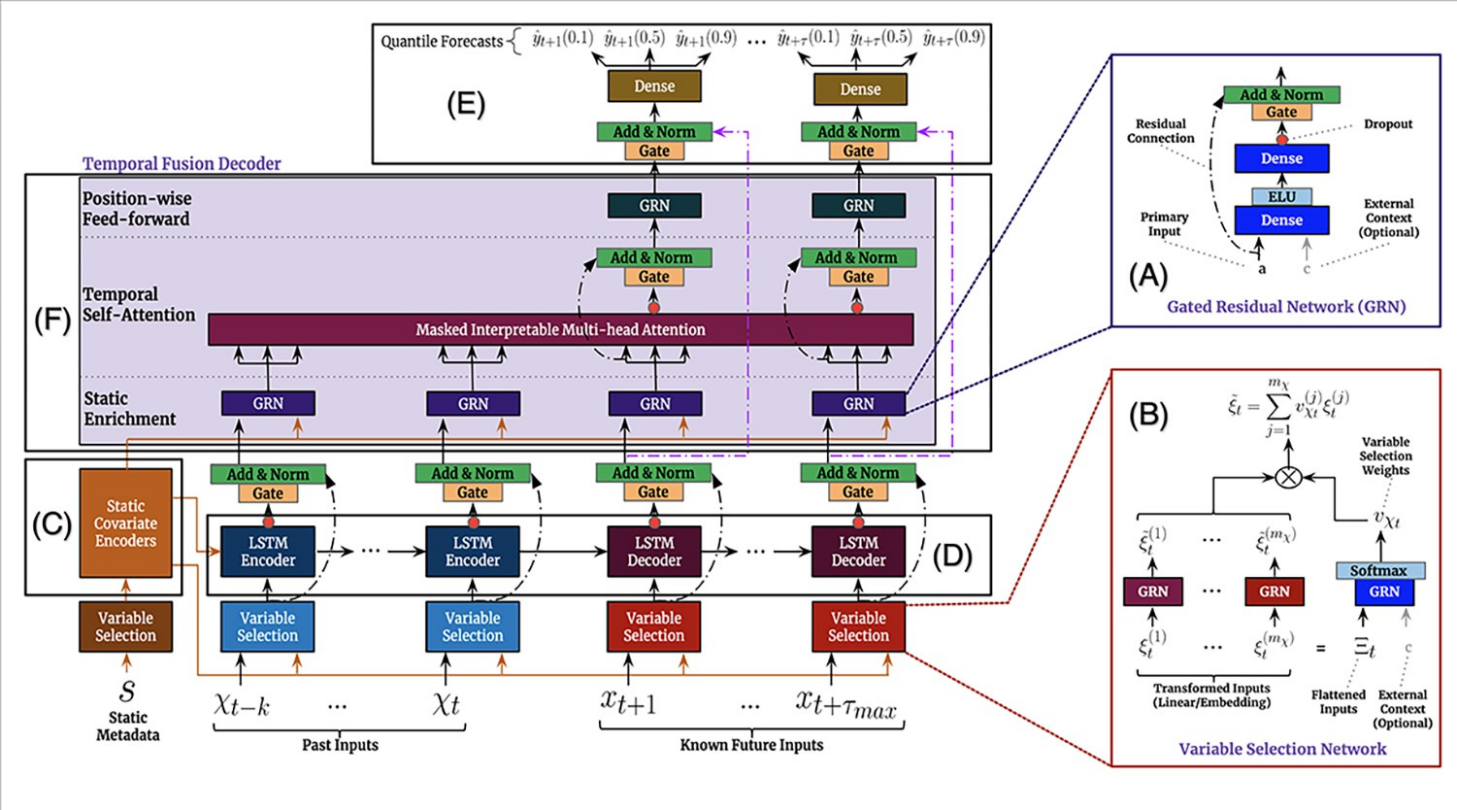
TFT model architecture [39]. The main components are described as follows [39]: Gating mechanisms (GRUs) for adaptive depth and network complexity (A); Variable Selection Networks (B); Static Covariate Encoders to integrate static features into the network (C); Temporal processing to learn both long and short-term temporal relationships (D); Prediction Intervals via quantile forecasts (E); Temporal Fusion Decoder built as a series of layers to learn temporal relationships present in the dataset (F).

At its core, TFT is a Transformer-based architecture. In traditional architecture, there are different “heads” to project the entrance into different subspaces of representation. As a disadvantage of this approach, weight matrices have no common ground and cannot be interpreted. In the case of a model based on “Multi-Head Attention”, a new matrix is added, in such a way that the different Heads will share some weight values, which can then be interpreted in terms of seasonality analysis [40]. Fig 6 illustrates the architecture of a TFT network, as well as its main components.

The architecture of the basic building blocks found in the TFT model is specialized in finding different aspects or patterns in the time series:

- A temporal “Multi-Head Attention” block that identifies the long-range patterns that the time series may contain and prioritizes the most relevant patterns.
- LSTM sequential encoders/decoders summarize shorter patterns, to identify time-step relationships with their surrounding values while long-range relationships are left to attention modules.
- Closed residual network blocks are used to eliminate unimportant inputs in addition to preventing over-adaptation.

#### N-BEATS (Neural Basis Expansion Analysis Time Series)

Introduced in 2019 by Oreshkin et al [41], the N-BEATS model is a hybrid deep learning model originally used in solutions aimed at predicting univariate time series, and has the title of the first pure deep learning model with prediction accuracy superior to that of traditional statistical methods, emphasizing that the model does not use specific components of time series [42]. It is an architecture based on the fusion of a statistical model (Exponential Smoothing) and a recurrent neural network of the LSTM type (Long Short-Term Memory). As a result of hybridization, the model (N-BEATS) presents superior results when compared to non-hybrid models, especially in predicting univariate sequences [43].

N-BEATS neural networks work in a "block" architecture, where parts of a time series are used as input (input), while the output (output) consists of future values obtained based on the input data. The basic building block–a stacked, multilayer, fully connected network with a nonlinearity function–predicts base expansion coefficients both forward (forecast) and backward (backcast). The operation of N-BEATs networks can be described as follows [41]:

1. Start the sequence of stacks, represented by a combination of several blocks, which must connect feedforward networks through forecast and backcast links.
2. The block must remove a portion of the input signal, subsequently working on the residual error.
3. A partial forecast is generated for each block, focusing on the local characteristics of the time series.
4. Transfer the partial forecast data to the next stack, which should identify non-local patterns along the entire time axis.
5. Finally, the partial (local) forecasts are grouped into a global forecast.

**Fig 7:**
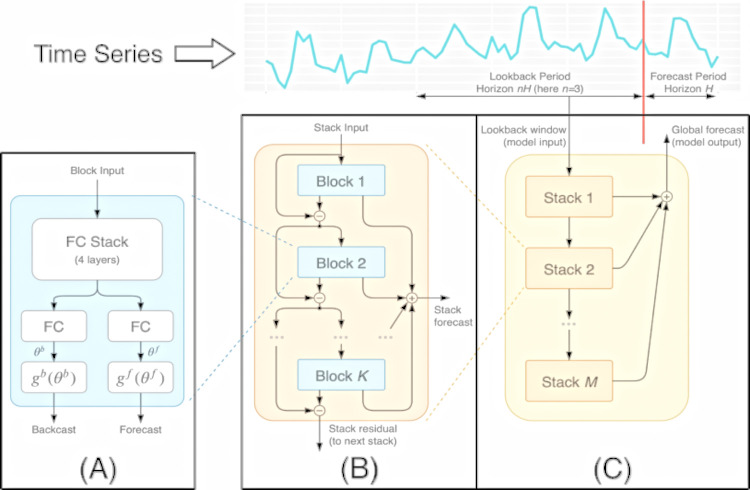
N-BEATS model architecture [41]. The main components are described as follows: The basic building block is a multilayer Fully-Connected network with nonlinearities based on the ReLu activation function (A). It predicts base expansion coefficients both forward (forecast) and backward (backcast). Blocks are arranged into stacks using the residual double stacking principle (B). A stack can have layers with shared gb and gf. Forecasts are aggregated hierarchically (C), where the first one is responsible to receive (input) a part of its respective time series (lookback window).

Fig 7 illustrates the architecture of an N-BEATS network, as well as its main components.

#### N-HiTS (Neural Hierarchical Interpolation for Time Series forecasting)

Derived from the N-BEATS algorithm, N-HiTS introduces a specialization scheme applied to “long-horizon forecasting” problems [44]. More specifically, the objective of the N-HiTS model is to improve the result of predictions whose period to be predicted is significantly large. The main technique applied by the model is the “multi-scale hierarchical interpolation”, which simplifies the forecasting process by reducing the dimensionality of the predictions [44]. Allied to this, multi-rate sampling is applied to the input data, resulting in a model whose accuracy is 25% higher when compared to the most recent Transformer architectures at the time of its creation, and whose processing time is reduced by 50x [44].

**Fig 8:**
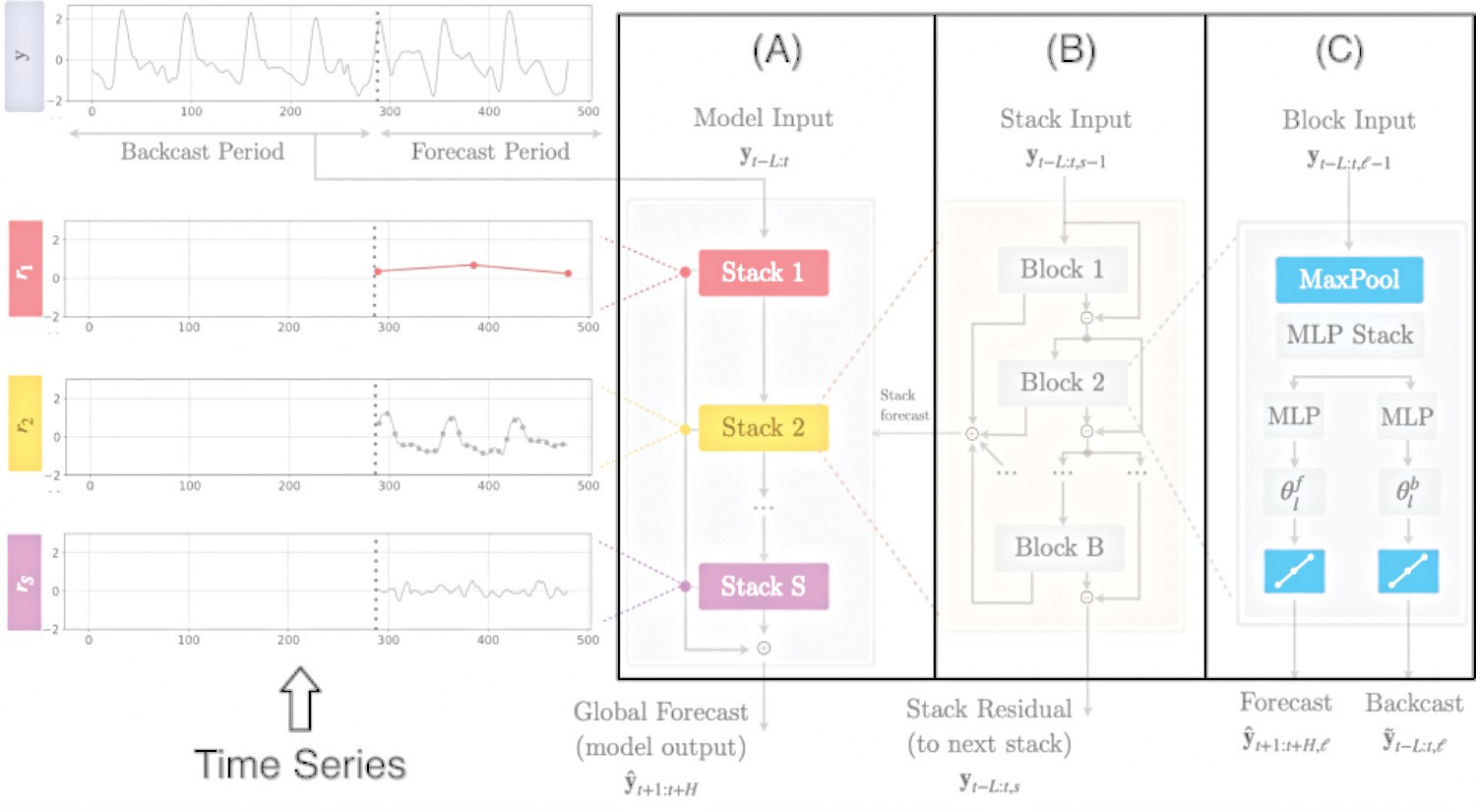
The architecture of the N-HiTS model [44]. According to [44], the network model consists of several Multi-Layer Perceptron (MLP) with nonlinear ReLU output functions, organized into a pool of blocks (B), located in a group of stacks (A). These blocks are connected via residual stacking principle (B) with the backcast and forecast, l-th outputs of the l-th block (C). Multi-rate input pooling, hierarchical interpolation, and backcast residual connections together induce the specialization of the additive predictions in different signal bands, reducing memory footprint and compute time, and improving architecture parsimony and accuracy.

The N-HiTS model is composed of a set of stacks specialized in learning–using several base functions–different characteristics of the data. The stacks are composed of a certain number of blocks containing a Multilayer Perceptron (MLP), which learns to produce coefficients both for the backcast and for the forecast of the data. From the results of each block, the backcast is used to clean up the entries of subsequent blocks, while the predictions are recorded for use in composing the final prediction. The model supports multivariate series, past covariates, and the production of probabilistic predictions (by specifying a probability pattern) [44]. Fig 8 illustrates the architecture of an N-HiTS network, as well as its main components.

### Post processing, evaluation, and selection of models

After training the five models by GridSearch, fifty historical predictions on the training data and fifty 7-day ahead predictions are generated for validation. The median of the 50 predictions is selected and a rescale is performed so that the values return to the original scale. It is checked if there are values less than 0, and if there are, values less than 0 are replaced by the number 0.01 (< 1) since it is unlikely that the number of deaths and cases is less than 0.

To evaluate the models, error metrics were calculated from the training and validation dataset (in this context the validation dataset is similar to the test data). For the purposes of this article, the following metrics were used: mean squared error (MSE; Eq 1), root mean squared error (RMSE; Eq 2), mean absolute percentage error (MAPE; Eq 3), symmetric mean absolute percentage error (sMAPE; Eq 4), coefficient of determination (*r*^2^; Eq 5), and coefficient of variation (*Coef*_*var*_; Eq 6).


$$MSE=\frac{1}{n}\sum_{t=1}^{n}{\left(y_{t}-{\hat{y}}_{t}\right)}^{2}$$ (1)

$$RMSE=\sqrt{\sum_{t=1}^{n}\frac{{\left(y_{t}-{\hat{y}}_{t}\right)}^{2}}{n}}$$ (2)

$$MAPE=100\frac{1}{n}\sum_{t=1}^{n}\left|\frac{y_{t}-{\hat{y}}_{t}}{y_{t}}\right|$$ (3)

$$sMAPE=200\frac{1}{n}\sum_{t=1}^{n}\frac{\left|y_{t}-{\hat{y}}_{t}\right|}{\left|{\hat{y}}_{t}|+|y_{t}\right|}$$ (4)

$$r^{2}=1–\frac{\sum_{t=1}^{n}{\left(y_{t}-{\hat{y}}_{t}\right)}^{2}}{\sum_{t=1}^{n}{\left({\bar{y}}_{t}–y_{t}\right)}^{2}}$$ (5)

$$Coef_{Var}=100\frac{\sqrt{\frac{1}{n}\sum_{t=1}^{n}{\left(y_{t}-{\hat{y}}_{t}\right)}^{2}}}{{\bar{y}}_{t}}$$ (6)

A forecast error means an unpredictable component of an observation, given by $\left(e_{T+h}=y_{T+h}-{\hat{y}}_{T+h|T}\right)$, being the training data {*y*_1_,…,*y*_*T*_} and validation data {*y*_*T*+1_, *y*_*T*+2_,…}. The variables found are defined as follows: Time index (*t*); Element of actual sequence (*y*_*t*_); *t-th* element of predicted sequence (${\hat{y}}_{t}$); the average of sequence ${\bar{y}}_{t}$.

It is important to emphasize that, in this case, there are the adjustment errors, which are calculated in the training set, and the prediction errors, calculated from the validation set. In this way, the residual metrics were calculated: mean, standard deviation, and the angular coefficient of the trend generated by linear regression of the residues, where the closer to zero, the better the model is evaluated. In other terms: The smaller the errors (of the various metrics); the closer to zero the mean, standard deviation, and angular coefficient of the residuals; best is evaluated in a given model.

In this process, the models for the training and validation data are scored according to the selection of the sum between the best-weighted values of the error metrics and the best-weighted values of the residual analysis. This computation generates a “score” value for the models, both for the training and validation data, as per Algorithm 1.

**Algorithm 1:** Calculate Model Score (input: A set of columns vector with the metrics values for each model)

1: For each column vector of the values of a metric *n*, of the *m* models

2:    Sort column vector from best to worst value

3:   Apply an equidistant normalization between 0 and 1 on the ordered column vector, resulting in a new column vector

4: For each new sorted vector of all calculated metrics

5:     Realize the sum between the new column vectors of the sorted metrics, respecting the matching between the models, and generating a single-column vector that represents the overall Score of the models

6: Return a column vector with the score for each model

**Fig 9:**
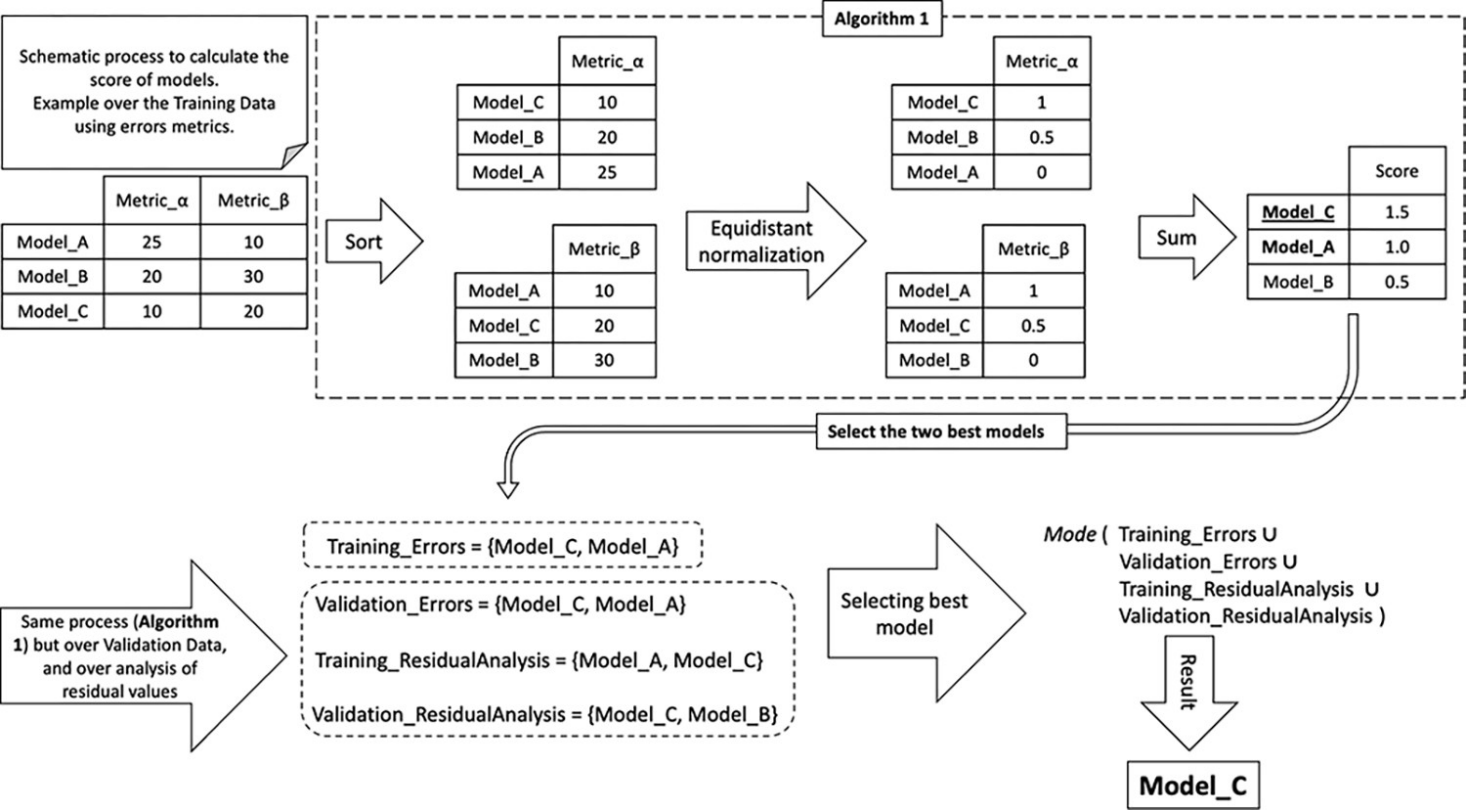
Example schematic of the process to calculate the score of models and select the best model based on training and validation data, in the error’s metrics, and the metrics of the residual values analysis.

With the score defined, the two best models are selected based on this score for each data set (training and validation), forming a small pre-selected group of best models. The model that was most frequent in this pre-selection is the chosen model. If there is a tie, the best validation data values are considered, and if there is still a tie, the one with the best MSE will be chosen. An example sample of this process is described in Fig 9.

In this sense, the best-evaluated model is selected, and used for the projection 7 days ahead of the last horizon day, using all the available time series and its auxiliary variables as input to the selected model.

### Diagnosis of residuals and smoothing of projections by ARIMA

The time series models used in this research allow the inclusion of information from past observations of a series and relevant information from other variables. From these advantages, it is possible to relate with the ASIMOV approach [45], in which Biecek [46] proposes three requirements for any predictive model, namely: prediction validation, prediction justification, and prediction speculation.

For the purposes of this study, the rationale is fulfilled by understanding which variables affect the prediction to what extent. In the background, speculation is the understanding of how the forecast would change if the values of the variables included in the model changed, and thus, compliance with the proposed framework is expected. Finally, validation is the verification of how strong the evidence is that supports the prediction and is typically accomplished by diagnosing the residuals.

Thus, given the importance of analyzing the residuals generated by the models, in summary, we are interested in evaluating the 4 well-known properties in the literature, such as (1) the autocorrelation of the residuals; (2) the mean of the zero-valued residuals; (3) homoscedasticity and finally, (4) the normality of the residuals. Good prediction models will produce residuals that meet the 4 properties; however, a crucial issue is fulfilling the validation when the properties are not met. Therefore, it is possible to extract the subtle dynamics of time series that can be treated with ARIMA (Autoregressive Integrated Moving Average) models [47].

The great contribution of this analysis is, in large part, to extract the terms from the non-seasonal and seasonal part of the residuals and, consequently, to obtain the improvement of the residual diagnoses. Then, given that the forecast function $\left(y_{t}={\hat{y}}_{t}+\eta_{t}\right)$, for a given model, we have *η*_*t*_ which is white noise, and which must meet the properties of the residues. By assuming that the series of errors *η*_*t*_ follow an ARIMA (1,1,1) model, for example, we can write the following Eq 7 and Eq 8:

$$y_{t}=\beta_{0}+\beta_{1}{\hat{y}}_{t}+\eta_{t}$$ (7)

$$\eta_{t}=\eta_{t-1}+\phi_{1}\eta_{t-1}+\theta_{1}\varepsilon_{t-1}+\varepsilon_{t}$$ (8)

where: *η*_*t*_ is the error from the best model, and (*ε*_*t*_) is the error from the ARIMA model. *β*_0_ (intercept), *β*_1_ slope or trend coefficient, and (*ϕ*_1_, *θ*_1_) are the coefficients of the autoregressive (AR) and moving average (MA) part, respectively.

Notice that the model has two error terms here—the error from the regression model, which we denote by *η*_*t*_, and the error from the ARIMA model, which we denote by *ε*_*t*_ [48]. Only the ARIMA model errors are assumed to be white noise [49]. Therefore, the objective of this step is to adjust the projection of the selected model based on the projection residuals on the observed data and projection 7 days ahead of the observed horizon, composing an essential post-processing step for the proposed framework.

## Results and discussion

The next topic will present the aspects of the conducted experiments, as well as the results obtained by using the aforementioned data in the five machine learning algorithms (see "Network Models’ section).

### Framework experimentation

**Fig 10:**
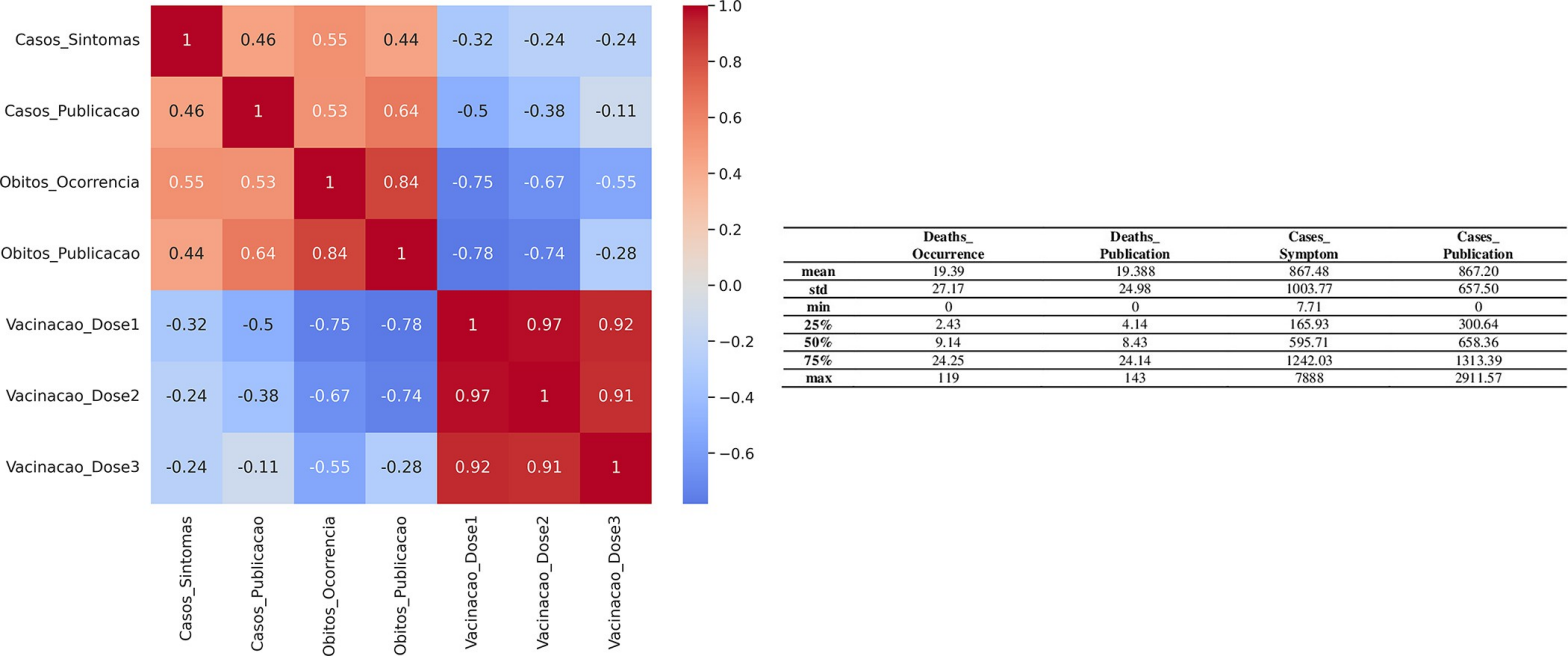
Heatmap with the correlation between the main series of the dataset and table with the descriptive statistics of the main series of the dataset containing 974 records.

For this first experiment, it is relevant to analyze the correlation between the variables and the previous descriptive statistics, as shown in Fig 10.

It is observed that the variables related to deaths and cases are negatively correlated with the vaccination variables, which suggests that the first dose of the vaccine possibly had a significant weight in the containment of deaths and cases in the state of Pará.

In this experiment, the process of training the models and their prediction took just over 6 hours and had the best parameters described in Table 4. The experiment was performed on a machine with an Intel Core i9 9980HK processor with 8 cores/16 threads at 2.4 GHz (clock base) and up to 4.2 GHz (TurboBoost); 2TB SSD; 64 GB of 2500 MHz LPDDR4 RAM; NVIDIA GeForce RTX 3080 Ti 12GB GDDR6X eGPU (Thunderbolt 3).

**Table 4:** Best parameters of each model for experimental execution.

Models	Parameters	Parameter values (cases | deaths)
TCN	kernel_size	4 | 3
	num_layers	2 | 1
	num_filters	4
	weight_norm	False
	dropout	0.05
	likelihood	GaussianLikelihood
TRANSFORMER	nhead	2
	num_encoder_layers	3 | 4
	num_decoder_layers	4 | 3
	activation	Gelu | ReLu
	dropout	0.1 | 0.05
	likelihood	GaussianLikelihood
TFT	lstm_layers	3
	num_attention_heads	4
	dropout	0.05 | 0.1
	likelihood	GaussianLikelihood
NHITS	num_layers	3
	num_blocks	2
	activation	ReLu
	dropout	0.1 | 0.05
	likelihood	GaussianLikelihood
NBEATS	num_layers	2
	num_blocks	1 | 2
	activation	Tanh | ReLu
	dropout	0.1
	likelihood	GaussianLikelihood

### Model analysis and selection

In this experiment, the calculated errors have the values shown in Table 5, for the prediction of publication of cases, publication of deaths, occurrence of deaths, and cases by date of symptom onset. It is observed that the NHITS model reached a better score, obtaining values for the metrics MSE, MAPE, sMAPE, and R2 better than the other models for the time series of published cases. This does not imply that NHITS will be the best model for all possible implementations of the framework, but it does indicate better modeling of the behaviors of these time series. It is important to note that, typically, training data is used to estimate any parameters of a prediction method and test data is used to assess its accuracy, the latter being a reliable indication of how well the model is likely to predict new data. Table 6, on the other hand, indicates the statistical data of calculated residual metrics for each model and each target series.

**Table 5:** Error measures for the training and validation dataset for the predicted series.

	Cases Publication time series													
Models	Training Data							Validation Data						
	MSE	MAPE	SMAPE	RMSE	R2	COEF_VAR	SCORE	MSE	MAPE	SMAPE	RMSE	R2	COEF_VAR	SCORE
NBEATS	28456,57	19,42	18,53	155,53	0,92	19,49	1,50	3375,26	41,66	17,67	32,05	-12,52	3,7	3,5
NHITS	24189,58	16,38	15,99	191,72	0,94	20,37	4,00	1174,39	39,32	24,37	71,13	-14,45	7,78	2,5
TCN	36755,99	17,72	17,69	168,69	0,94	22,15	1,75	4539,76	29,2	34,36	58,1	-65,56	3,96	1,75
TFT	26574,06	18,28	17,07	163,02	0,91	17,97	2,75	5059,78	17,08	19,2	67,38	-43,4	8,22	2,25
TRANSFORMER	31109	22,23	15,48	176,38	0,93	18,83	2,50	1027,39	17,58	49,2	34,27	-58,72	6,71	2,5
	Cases Symptoms time series													
NBEATS	116481,4	49,11	34,39	385,73	0,89	37,09	3,25	7328,26	272,79	117,34	22,77	-14,79	2,63	2,75
NHITS	35598,8	58,93	39,34	561,83	0,97	44,54	3,25	516,18	345,32	128,27	85,61	-222,28	9,88	1,75
TCN	315648,1	36,61	40,99	341,29	0,9	64,87	2,00	518,25	97,53	122,77	54,59	-89,8	6,3	3,25
TFT	103177,9	77,03	61,24	188,68	0,86	39,41	1,50	2980,1	225,86	80,3	91,49	-14,73	10,56	3,00
TRANSFORMER	148790,3	38,83	39,42	321,21	0,7	21,79	2,50	8369,55	98,3	191,58	22,72	-254	2,62	1,75
	Deaths Publication time series													
NBEATS	70,14	6502,71	37,00	9,34	0,86	48,18	3,50	9,98	507,40	140,51	3,16	-3993,07	16,29	1,25
NHITS	87,70	399954,4	39,70	8,37	0,88	55,97	1,75	1,06	377,22	136,77	3,65	-423,04	11,75	4,00
TCN	117,77	242981,0	51,36	9,36	0,89	44,94	2,50	6,60	609,80	85,22	2,28	-2639,25	18,82	2,00
TFT	87,26	552571,5	35,74	8,71	0,81	48,30	2,00	13,31	151,59	126,55	2,57	-5326,44	5,31	2,75
TRANSFORMER	75,93	590990,5	36,68	10,85	0,86	43,19	2,75	5,19	433,95	150,31	1,03	-2077,32	13,25	2,50
	Deaths Occurrence time series													
NBEATS	73,56	352,77	64,63	8,03	0,87	57,60	2,25	2,84	685626,6	0	1,17	0	8,69	2,00
NHITS	64,47	15196,92	45,22	11,17	0,90	64,12	2,50	0,02	0	142,85	0,51	0	2,62	3,25
TCN	154,58	4444,48	42,14	12,43	0,91	50,53	2,75	0	7684803	142,86	1,69	0	0,70	2,25
TFT	124,75	36,89	33,91	9,80	0,83	44,23	3,25	0,26	3140365	199,96	0,14	0	0	2,75
TRANSFORMER	96,00	72146,39	46,32	8,58	0,80	41,41	1,75	1,38	12925581	114,23	0	0	6,05	2,25

**Table 6:** Score, standard deviation, mean, and trend slope of the training and validation data residuals for the predicted series.

	Cases Publication time series							
Models	Training Data				Validation Data			
	ANGLE_COEF	MEAN	STD	SCORE	ANGLE_COEF	MEAN	STD	SCORE
NBEATS	-0,12	-74,70	151,33	1,00	-14,69	-38,39	47,10	0,50
NHITS	-0,10	-75,60	135,99	1,25	-2,96	5,69	36,50	2,00
TCN	0,03	16,75	191,09	1,75	14,43	66,40	12,37	1,75
TFT	-0,05	-20,22	161,85	1,50	-14,65	-70,09	13,10	1,00
TRANSFORMER	-0,02	-19,94	175,34	2,00	14,43	66,40	12,37	1,75
	Cases Symptoms time series							
NBEATS	0,06	31,15	340,06	1,00	-7,77	-62,92	62,69	1,00
NHITS	0,14	100,24	159,93	1,00	0,08	5,90	23,70	2,75
TCN	0,00	1,21	562,13	2,00	4,42	22,18	5,52	2,50
TFT	0,02	29,47	320,03	2,00	-12,20	-41,38	38,46	1,25
TRANSFORMER	0,04	18,20	385,51	1,50	-14,12	-67,42	66,79	0,00
	Deaths Publication time series							
NBEATS	0,00	-0,25	8,38	2,25	-0,67	-3,00	1,06	0,50
NHITS	0,00	0,36	9,36	2,00	-0,21	-0,73	0,78	2,25
TCN	-0,01	-3,39	10,31	0,00	-0,59	-2,56	0,21	2,00
TFT	0,00	2,18	9,09	1,50	-0,76	-3,61	0,55	0,75
TRANSFORMER	0,00	-0,50	8,70	1,75	-0,53	-2,18	0,70	2,00
	Deaths Occurrence time series							
NBEATS	0,00	0,71	8,55	2,25	-0,22	-1,29	1,17	0,00
NHITS	0,00	0,86	7,99	2,75	-0,01	-0,07	0,13	2,25
TCN	0,00	5,75	11,03	0,00	0,00	0,00	0,00	3,00
TFT	0,00	4,24	10,34	0,75	-0,03	-0,31	0,43	1,50
TRANSFORMER	0,00	1,36	9,71	1,75	-0,10	-0,77	0,96	0,75

After applying Algorithm 1, the TCN Model was selected with the best scores for the Cases_Symptoms series. The NHITS Model was the best model for the series Cases_Publication, Deaths_Occurrence, and Deaths_Publication. After selecting the models, all the observed data are used to forecast 7 days ahead of the observed horizon, with post-processing together with dynamic regression (method with ARIMA), acting to adjust the generated projections. The other prediction graphs on the training and validation data and projections of the selected models (without the effect of the application of ARIMA) are present in S1 File of this study.

### Residual diagnosis of projections

Carrying out the diagnosis of residuals helps us to understand the behavior of the projections and the best models selected for the four-target series of the present study. In the body of this article, we present an analysis of each target series and their respective projections generated by the selected models with the effect of the application of ARIMA. Residual information, or white noise, is also presented, displayed in graphs of residual values, residual distribution, ACF plot (Autocorrelation Function), and Q-Q Plot (Quatile-Quantile). Thus, the final projections of this experiment were generated and demonstrated individually in the following subsections. More information about the residuals of each subseries (residuals about the training and validation data without the effect of the application of ARIMA) is available in S2 File.

### Publication of daily cases

**Fig 11:**
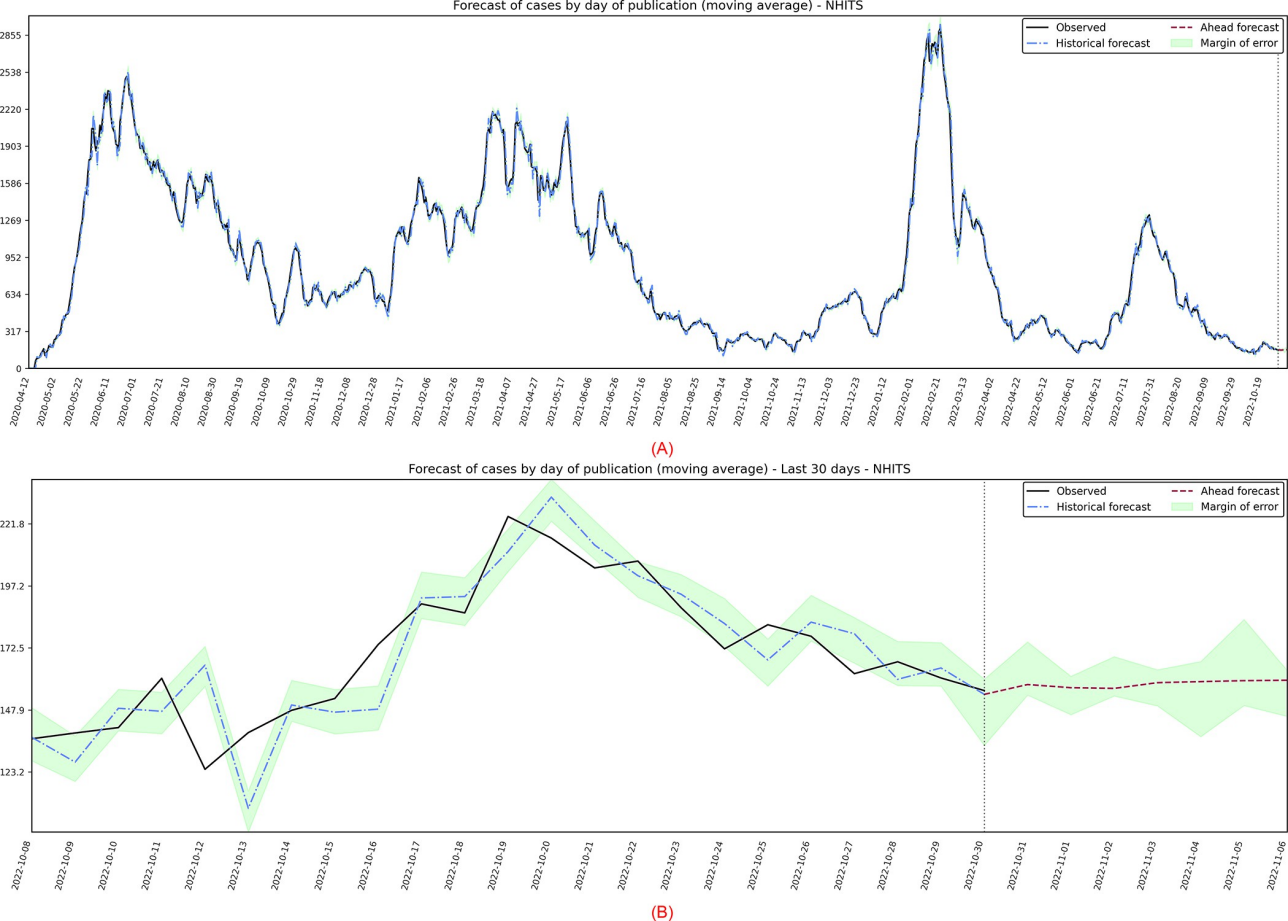
(A) Projection referring to the moving average of daily published cases. (B) 7-day forward projection showing the last 30 days.

The first target series is the cases published per day, in which the best performing model was the NHITS, with projections presented in Fig 11(A). In particular, there is great volatility in the data, which makes it difficult to visually verify the stationarity of the data, in addition to the fact that, for the pandemic context, it is expected that there will be both peaks in cases and a decreasing trend over time, given by the containment and preventive measures adopted by managers.

When running the Hyndman-Khandakar algorithm [49] for automatic ARIMA modeling, for the residuals, without intercept, an ARIMA model was obtained with a non-seasonal part (1,1,2) and a seasonal part (1,0,1) with a period of 7 days. The coefficients and their standard errors (*se*) are presented in Table 7.

**Table 7:** Report of estimated parameters and standard errors modeled by ARIMA (publication cases).

	ar1	ma1	ma2	sar1	sma1
Estimated	0.9656	-0.4244	-0.2594	0.2199	-0.8131
s.e.	0.0123	0.0345	0.0341	0.0653	0.0523

**Fig 12:**
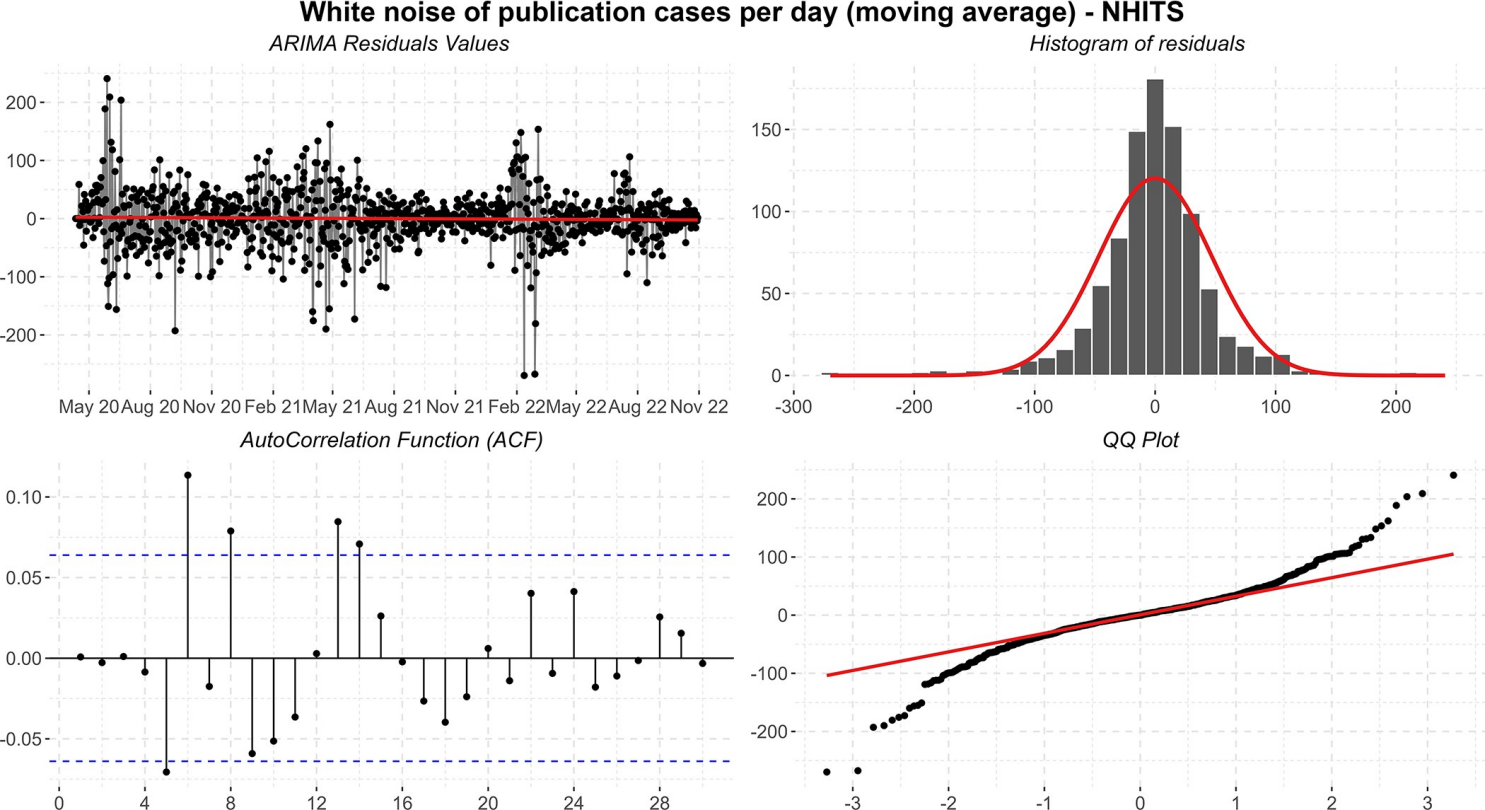
Residual diagnostic graphs for the ARIMA method applied to the errors of publication cases.

Then the residuals are plotted in time series, histogram, ACF, and QQ-plot (Fig 12). The average of the residuals is close to zero, but there is clear heteroscedasticity, with greater variance in June/2020, May/2021, and February/2022, and lower variance in October/2022. The model also shows significant autocorrelation in the residuals, and the histogram shows long tails but suggests normality.

Using the estimated model, a forecast was made for 7 days ahead, referring to the period from 31/10/2022 to 06/11/2022. According to Fig 11(B), the punctual forecasts seem reasonable for the 7 days in which it shows a slight incline. Despite the problems presented by residuals, point predictions are expected to be correct, however, prediction intervals assuming a normal distribution can be inaccurate.

Finally, the precision measures for this period are calculated. When comparing with Table 7, residual modeling produced improvements in the accuracy metrics for observed data, with a value of *MSE* = 2199.41; *MAPE* = 4.44; *sMAPE* = 4.51; *RMSE* = 46.90; *R*2 = 0.99; *Coef*_*var*_ = 5.18.

### Cases by day of the first symptom

**Fig 13:**
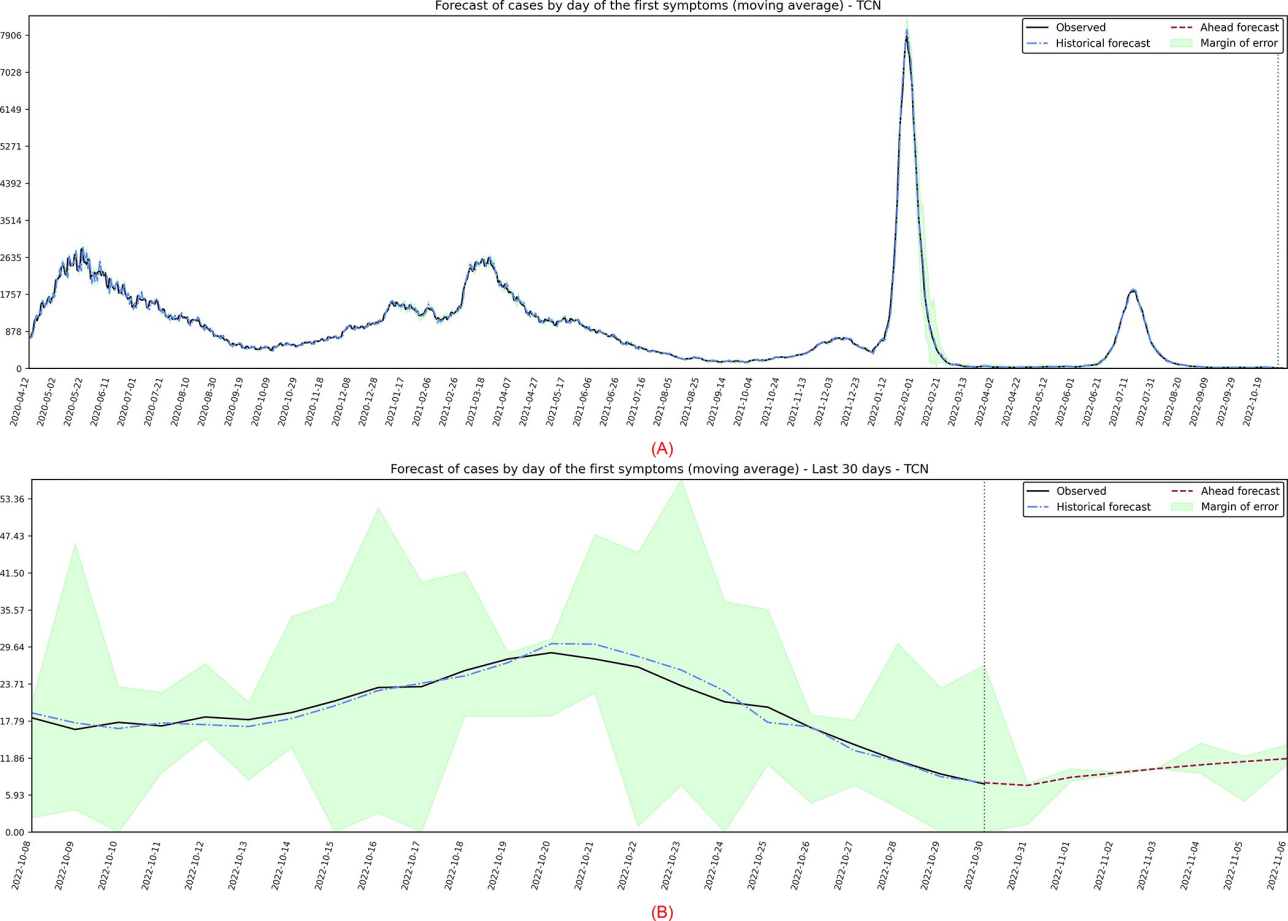
(A) Projection referring to the moving average of cases by day of symptoms. (B) 7-day forward projection showing the last 30 days.

The second target series analyzed are the cases registered from the first day of symptoms, in which the best performing model was the TCN. By analyzing the projections in Fig 13(A), it’s possible to infer that the data is clearly decreasing and not stationary, so some kind of differentiation might be needed.

When executing the algorithm for automatic ARIMA modeling, without intercept, it was obtained a model *ARIMA*(1,1,3)(2,0,0)_7_. Their coefficients and standard errors (*se*) are presented in Table 8.

**Table 8:** Report of estimated parameters and standard errors modeled by ARIMA (symptomatic cases).

	ar1	ma1	ma2	ma3	sar1	sar2
Estimated	0.9387	-0.6239	-0.067	0.3888	-0.5312	-0.3511
s.e.	0.0125	0.0326	0.035	0.0301	0.0322	0.0315

**Fig 14:**
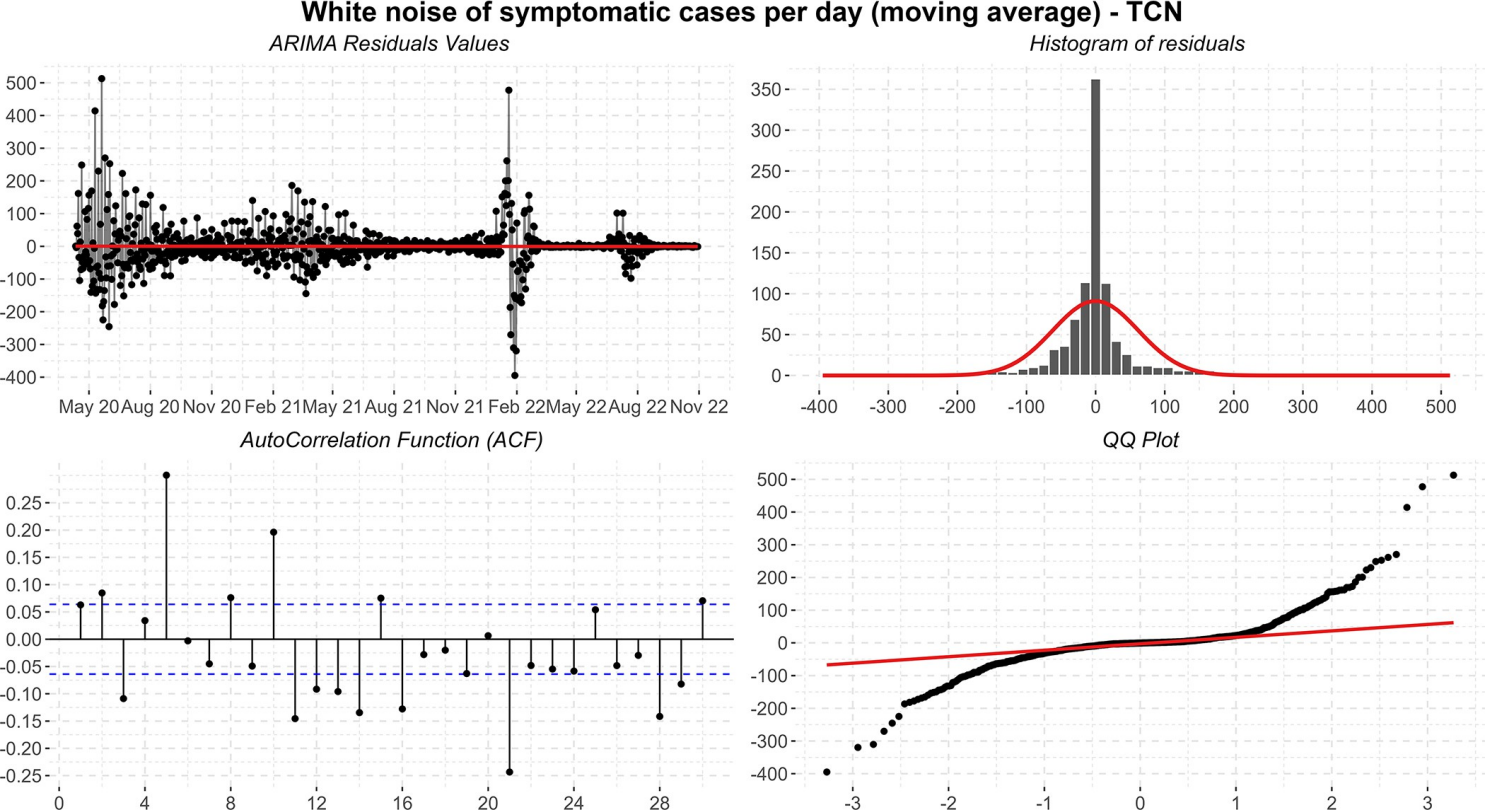
Residual diagnostic graphs for the ARIMA method applied to the errors of symptomatic cases.

Then the residuals are plotted in time series, histogram, ACF, and QQ-plot (Fig 14). Although the short-term forecasts seem reasonable, the residuals demonstrate that there may still be information that was not captured by the model and, despite the robustness of the modeling and a complex process, none of the evaluated models pass all the residual tests. In practice, the presented configuration refers to the model that behaved better during training and, broadly speaking, it is reasonable to think that there are several other sources of uncertainty not captured by the variables used.

Finally, the metrics discussed in the work showed significant improvements in the observed data. The calculated values are: *MSE* = 3840.33; *MAPE* = 4.18; *sMAPE* = 4.28; *RMSE* = 61.97; *R*2 = 1.00; *Coef*_*var*_ = 6.90.

### Publication of daily deaths

**Fig 15:**
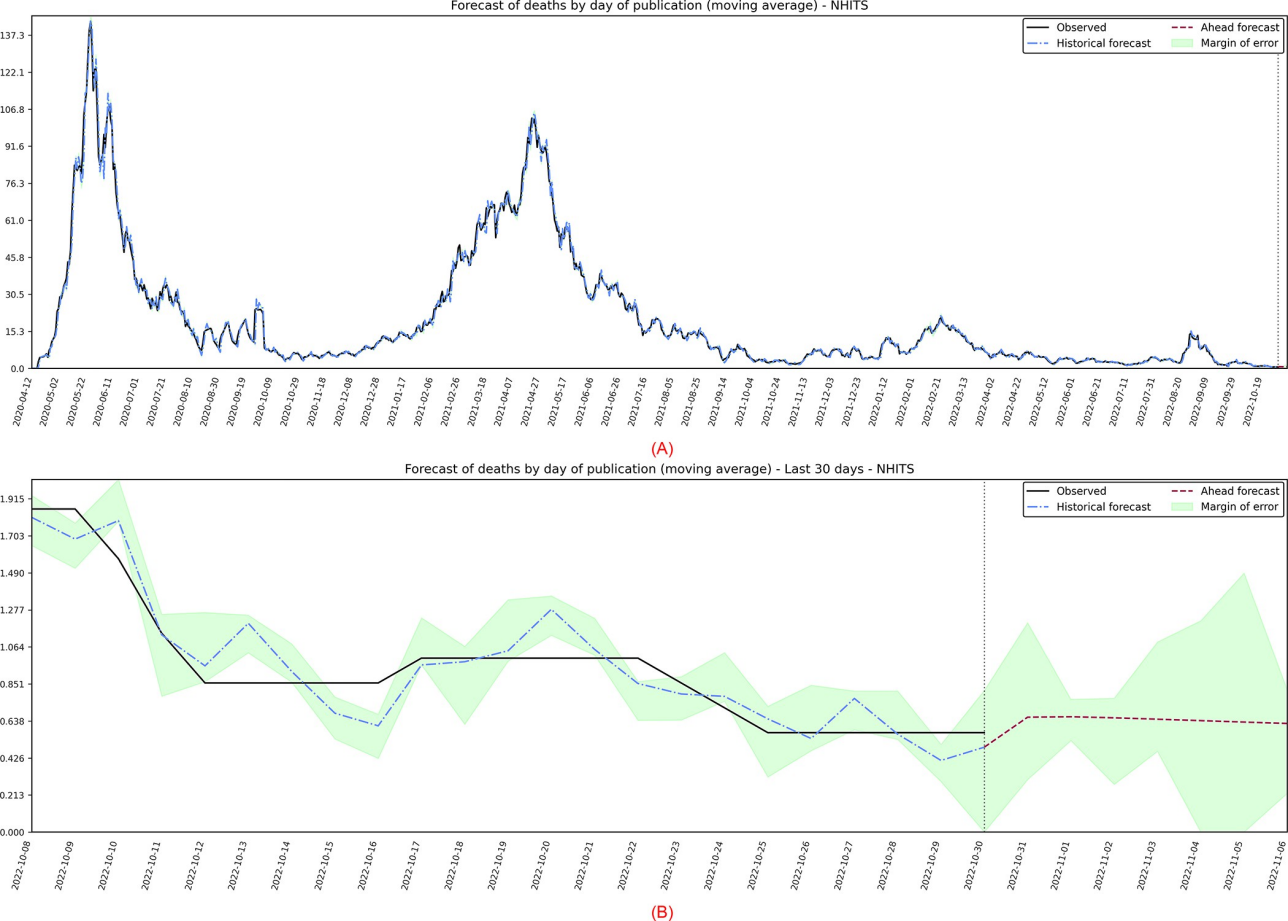
(A) Projection referring to the moving average of published daily deaths. (B) 7-day forward projection showing the last 30 days.

The third target series analyzed are the deaths published daily, in which the best performing model was the NHITS, with projections presented in Fig 15(A). It is observed that there are two strong peaks in the data and, as expected, a trend of stability in the data after October 2021. Using the estimated model, a forecast was made for 7 days ahead, referring to the period of 31/10/2022 on 06/11/2022. According to Fig 15(B), punctual forecasts seem reasonable for the 7 days, with the trend being maintained at values below 1. The error margins generate a greater amplitude on 05/11/2022, with a maximum of 1.49 deaths. It is important to notice that, in practice, the number of deaths will always be discreet, and the estimate is a reference value.

The algorithm for automatic ARIMA modeling, without intercept, it was obtained a model *ARIMA*(2,1,3)(0,0,1)_7_. Due to the peaks presented in the observed data, it was necessary to add a delay of 14 days for a better estimate of the parameters in the training phase and, consequently, to seek predictions with better accuracy. Their coefficients and standard errors (*se*) are presented in Table 9.

**Table 9:** Report of estimated parameters and standard errors with residuals modeled by ARIMA (deaths publication).

	ar1	ar2	ma1	ma2	ma3	sma1	lag (14)
Estimated	1.8667	-0.8731	-1.5679	0.4121	0.2097	-0.8610	0.0055
s.e.	0.0541	0.0531	0.0623	0.0854	0.0399	0.0254	0.0185

**Fig 16:**
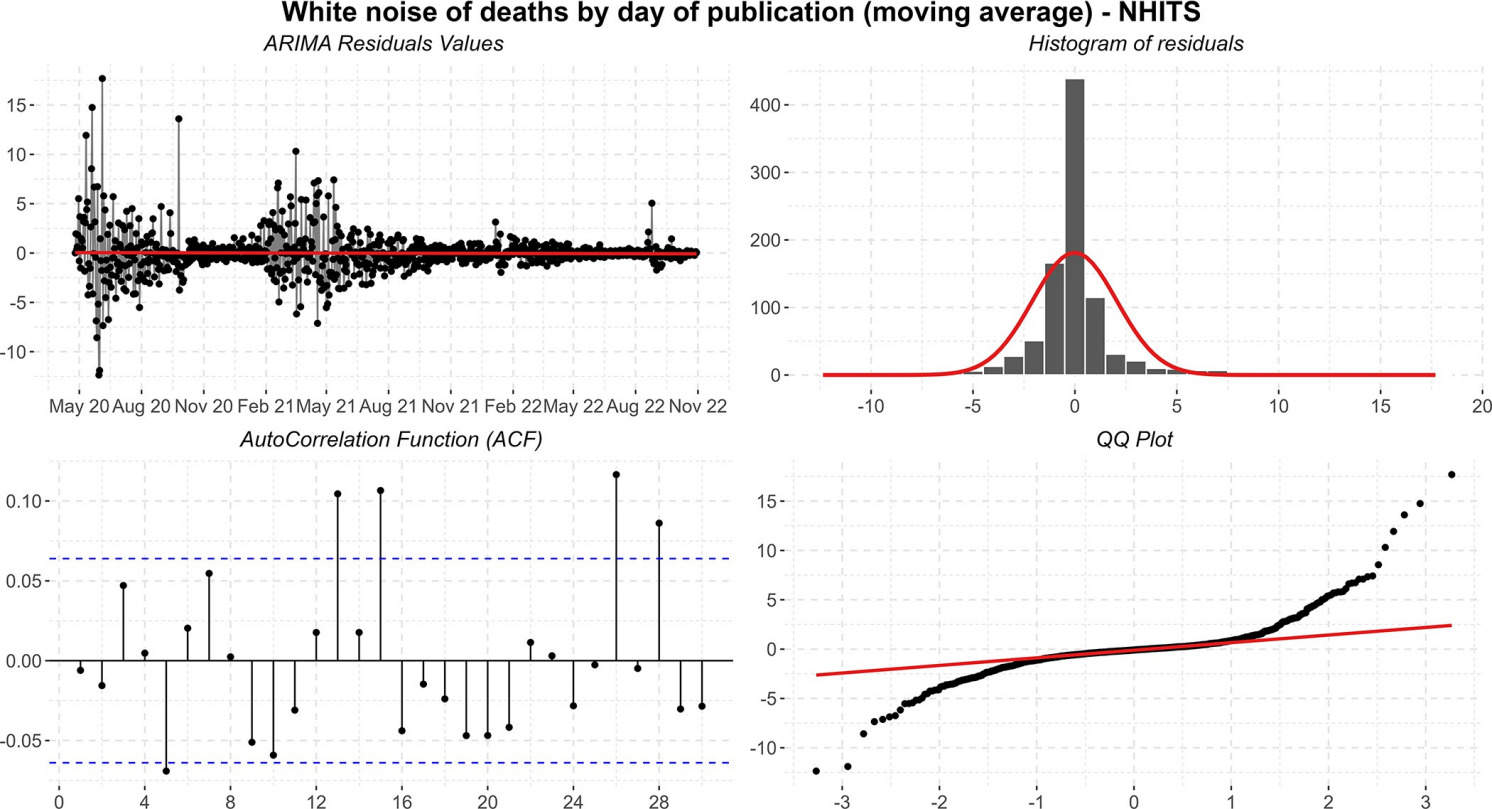
Residual diagnostic graphs for the ARIMA method applied to the errors of symptomatic cases.

In Fig 16, the residuals have heteroscedasticity problems, some significant autocorrelation of order 9 and 21, and the histogram tails are long. Positively, the histogram appears to be normally distributed, the residuals maintain zero mean and, despite the problems presented, it is reasonable to think that the cause comes from the peaks presented. In this way, due to the robustness of the analyses, the specific forecasts-maintained values consistent with what was expected by the pandemic.

Finally, the metrics discussed in the work showed significant improvements in the observed data. The calculated values are: *MSE* = 4.38; *MAPE* = 7.94; *sMAPE* = 8.01; *RMSE* = 2.09; *R*2 = 0.99; *Coef*_*var*_ = 10.34.

### Deaths by day of occurrence

**Fig 17:**
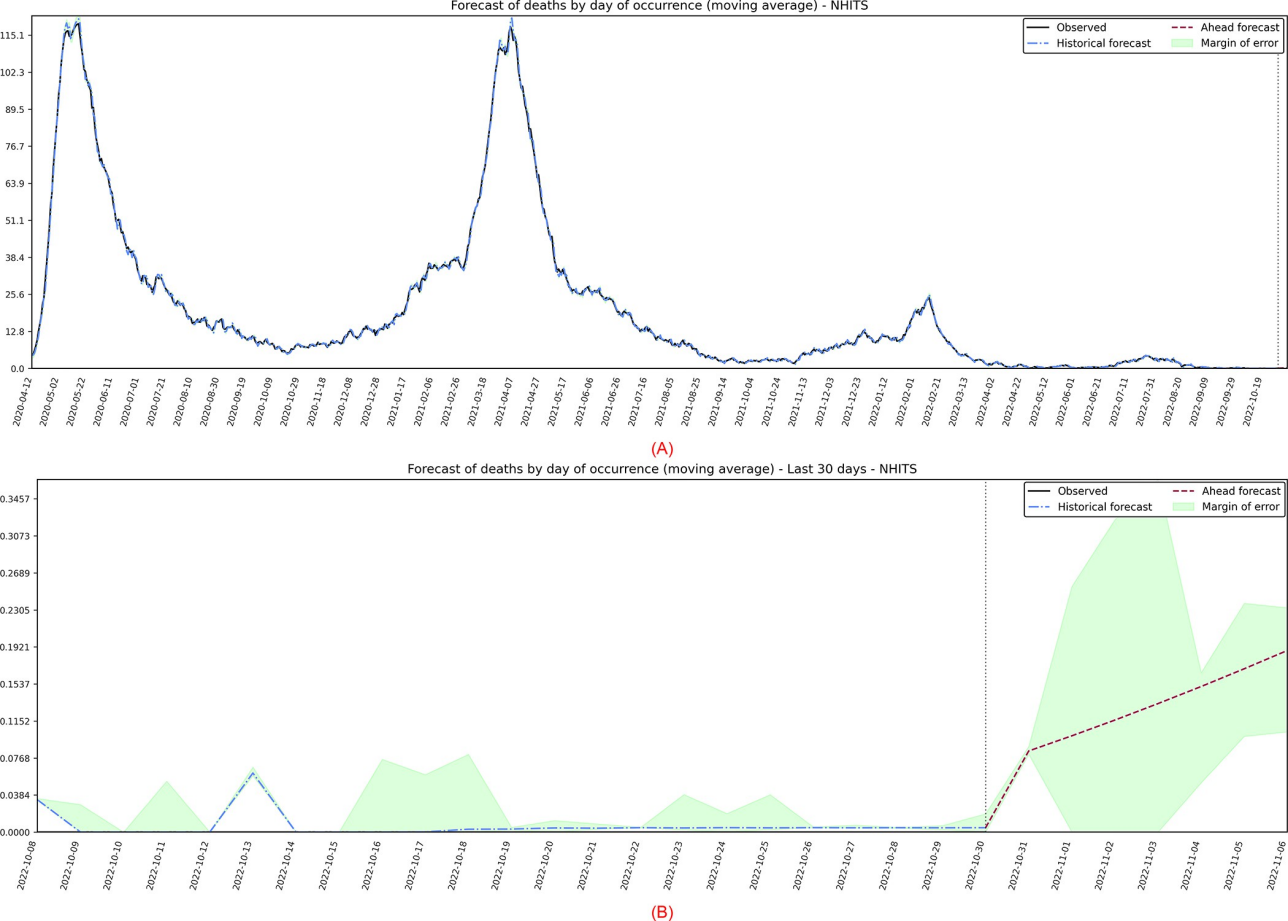
(A) Projection refers to the moving average of publication of deaths by the day of occurrence. (B) 7-day forward projection showing the last 30 days.

The fourth target series analyzed are the deaths registered on the day of the death, in which the best performing model was the NHITS, with projections presented in Fig 17(A). Similar to the publication series of daily deaths, two peaks are observed in May/2020 and April/2021, and a smaller peak in February/2022. As expected, there is a downward trend in these deaths and, according to Fig 17(B), a stabilization occurs in the final occurrences.

The algorithm for automatic ARIMA modeling, without intercept, it was obtained a model *ARIMA*(2,1,2)(2,0,0)_7_. Due to the peaks presented in the observed data, it was necessary to add a delay of 14 days for a better estimate of the parameters in the training phase and, consequently, to seek predictions with better accuracy. Their coefficients and standard errors (*se*) are presented in Table 10.

**Table 10:** Report of estimated parameters and standard errors with residuals modeled by ARIMA (occurrence of deaths).

	ar1	ar2	ma1	ma2	sar1	sar2	lag (14)
Estimated	0.0337	0.9244	0.3956	-0.4326	-0.5204	-0.3137	0.0165
s.e.	0.0313	0.0309	0.0462	0.0379	0.0332	0.0336	0.0164

**Fig 18:**
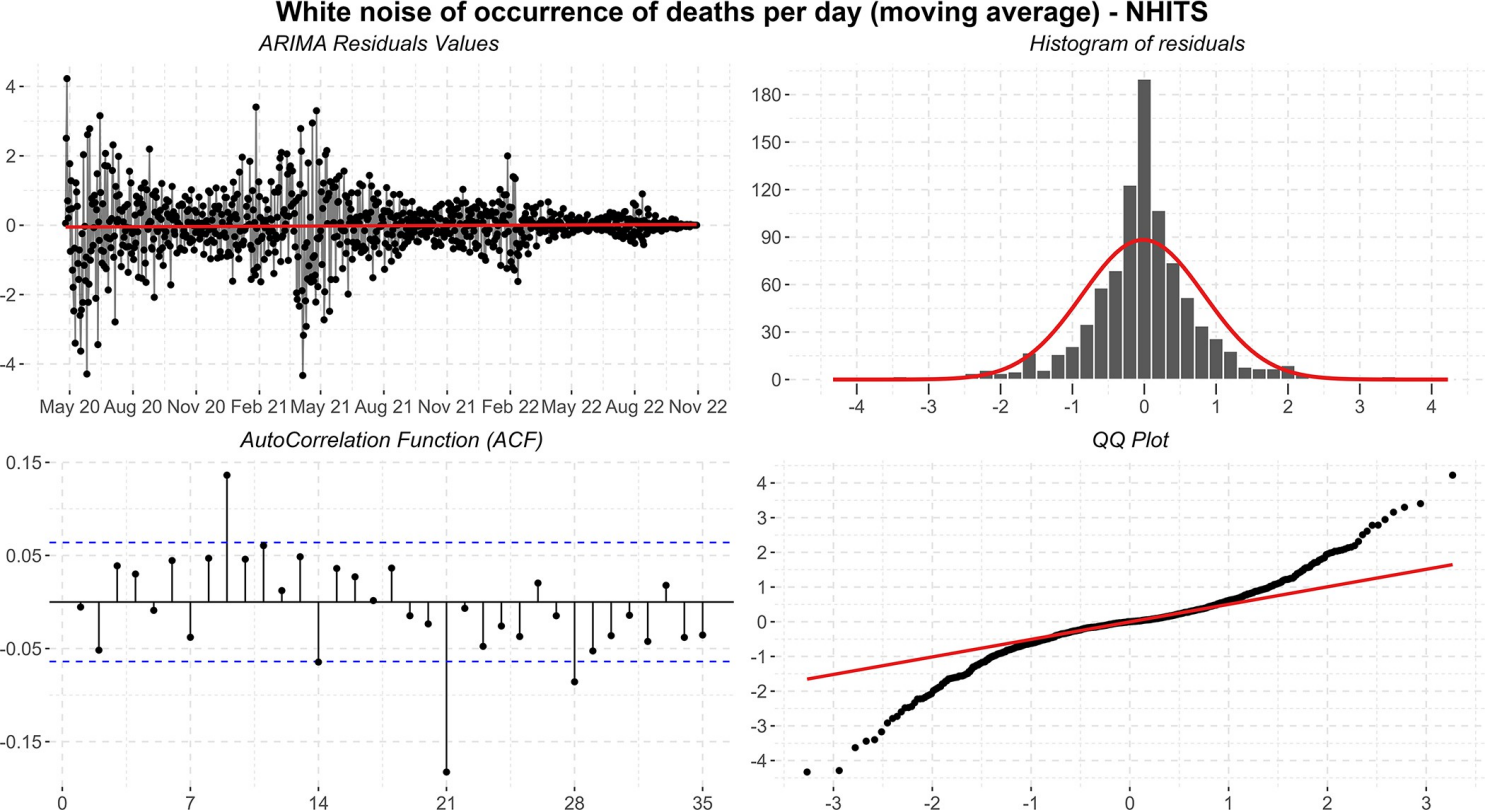
Residual diagnostic graphs for the ARIMA method were applied to the errors of occurrence of deaths.

In Fig 18, the residuals present heteroscedasticity problems due to the displayed peaks, some significant autocorrelation of order 9 and 21, and long tails in the histogram. Positively, the histogram appears to represent a normal distribution, and the residuals maintain zero mean. In this way, due to the robustness of the analyses, the specific forecasts-maintained values consistent with what was expected by the pandemic. In practice, the number of deaths in this series is quite discreet, predicting values lower than one at the end of the tail.

Finally, the metrics discussed in the work showed significant improvements in the observed data. The calculated values are: *MSE* = 0.72; *MAPE* = 15.17; *sMAPE* = 15.04; *RMSE* = 0.85; *R*2 = 1.00; *Coef*_*var*_ = 4.197.

### Model stability analysis

**Fig 19:**
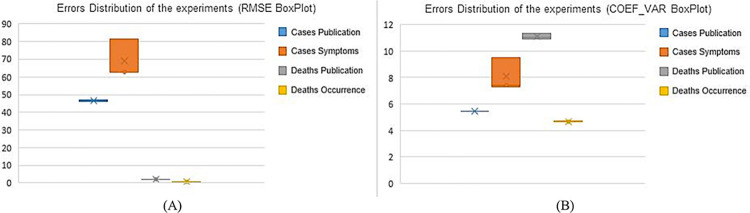
Residual Distribution of the experiments. (A) RMSE BoxPlot, (B) COEF_VAR BoxPlot.

In a highly changing pandemic environment, there is the need for constant adjustment of parameters with the rerun of models [50]. To analyze the stability of the framework, two more experiments were carried out in later periods. The data show the distribution of errors that can give us indications about the stability of the proposed approach (Table 11 and Fig 19).

**Table 11:** Error forecast evaluation of each experiment.

	**Cases Publication**			**Cases Symptoms**			**Deaths Publication**			**Deaths Occurrence**		
	MODEL SELECTED	RMSE	COEF_VAR (%)	MODEL SELECTED	RMSE	COEF_VAR (%)	MODEL SELECTED	RMSE	COEFF_VAR (%)	MODEL SELECTED	RMSE	COEF_VAR (%)
**1**^**st**^ **experiment (oct. 30 to nov. 11)**	**NHITS**	46.88	5.43	TCN	62.85	7.28	**NHITS**	2.10	10.94	**NHITS**	0.89	5.00
**2**^**nd**^ **experiment (nov. 13 to nov. 20)**	TFT	46.5	5.45	TFT	81.37	9.53	**NBEATS**	2.10	11.05	**NBEATS**	**0.88**	**4.65**
**3**^**rd**^ **experiment (nov. 27 to dec. 04)**	TRANSFORMER	46.25	5.47	**NHITS**	62.61	7.40	**NBEATS**	2.13	*11*.*36*	**NBEATS**	**0.88**	4.70
**Mean**	-	46.54	5.45	-	68.94	8.07	-	2.11	11.12	-	0.88	4.66
**SD**		00.32	0.02		10.76	1.27		0.02	00.22		0.01	0.04

A small standard deviation is observed between the projected series, but the Cases Symptoms series showed a larger standard deviation. This may indicate the need for further studies on this variable, with greater analysis of possible factors that interfere with the prediction of this series. It is worth noting that this data is collected by frontline professionals fighting the pandemic, usually doctors, nurses, or health care personnel. These professionals collect data via a paper questionnaire with questions such as “What was the day you had your first respiratory symptom?”. Another characteristic of this series is that, depending on the moment of the pandemic, such as pandemic peaks, health personnel are overloaded, which can generate damned data that are only recorded in the system at times when the epidemic peak has significantly decreased.

It should be noted that the Cases and Publication variables generally suffer from underreporting and delay in reporting, which is common in an Amazonian state with a large territory. This can influence the overall performance of the proposed approach. For the Cases Publication series, however, the collection of this variable is more objective, as its registration is done by laboratory verification of the Covid-19 test. Deaths Publication and Deaths Occurrence also have more objective data, the first being provided by the hospital’s health personnel when daily accounting for deaths in ICU beds, and the second via verification in registry offices.

Low values in relation to COEF_VAR (percentage measure) stand out, indicating an average error of 5.4% in Cases Publication, 8.0% in Cases Symptoms, 11.12% in Deaths Publication, and 4.6% in Deaths Occurrence. Thus, the proposed framework, based on the calculated error data, tends to behave better for the series Deaths Occurrence, Cases Publication, Deaths Publication, and Cases Symptoms, with the first three indicating less variation in the data, therefore more reliable.

## Conclusion

The objective of this study is to present a semi-automated framework for projecting the 7-day Moving Average for COVID-19 cases (by the day of the first symptom and by the day of publication) and deaths (by day of occurrence of death and by the day of death publication) of COVID-19, using global and advanced deep learning models that are probabilistic and that allow the use of multivariable and auxiliary variables. It is noteworthy that the generated projections showed low mean error for Cases Publication (5.4%) and Deaths Occurrence (4.6%), with the N-HiTS and N-BEATS models having better results, aided by the ARIMA statistical model as post-processing method for residual adjustment and short-term smoothing.

In addition to using the mentioned models, as far as we know, there is no framework that uses the characteristics described in this study. However, one of the benefits of the framework is the assistance to researchers and analysts in the epidemiological area, in order to serve as a prior analysis, similar to an epidemiological alert system [51]. For example, if the generated projections indicate a significant increase in cases and/or deaths, other analyzes can be carried out to prove a real increase, such as, for example, the regionalized geographically investigation (i.e., cities, states/provinces and country) of clinical beds and ICUs [51–53].

In conclusion, the use of deep learning models to predict cases and deaths from COVID-19 has proven to be a valuable practice for analyzing the spread of the virus, which allows health managers to better understand and respond to the pandemic. The satisfactory accuracy of these models indicates reliability and consequently makes them useful for decision-making, as they can help guide the development and implementation of effective public health policies and interventions. This is especially important given the rapidly changing nature of the COVID-19 pandemic and the need for timely and accurate information to inform decision-making, control planning, and mitigate the impact of the disease. Overall, this study highlights the importance of using advanced analytical techniques such as deep learning to better understand and respond to the COVID-19 pandemic.

As future contributions, the study areas can be expanded, as well as the period to increase the possibilities of comparative analyzes, aiming at a better understanding of the pandemic, in a more regionalized way. Another work to be done would be to add metaheuristics and better hyperparameter selection models to optimize deep learning models, which can further increase the performance and accuracy of the framework. Likewise, another contribution to the present framework can be to implement it in the form of a web service, where managers from different regions, states, and countries can enter their data and obtain the results to support their analyses. Thus, this research also becomes an important reminder of using data-driven approaches and the power of technology to address complex public health challenges.

## Supporting information

S1 File Prediction plots of the trained models. Presents the graphs of model predictions for the variables related to COVID-19 for the first experiment of this study. Each graph helps us understand the behavior of the predictions after training each model, tested for the 4-target series of this study: number of cases by publication date, number of cases by date of symptom onset, number of deaths by publication date, and number of deaths by date of death occurrence. However, without smoothing by ARIMA or post-processing on margin of error, just using deep learning models.
(DOCX)

S2 File Residual analysis graphs of the best models. Presents the graphs for the analysis of the residuals of the best projection models of variables related to COVID-19. Each graph helps us to understand the behavior of the projections and the best models selected for the 4-target series of the present study: number of cases by date of publication, number of cases by date of onset of symptoms, number of deaths by date of publication, and number of deaths by date of death occurrence.
(DOCX)
