# Supplementary material for: Deep learning framework for epidemiological forecasting: A study on COVID-19 cases and deaths in the Amazon state of Pará, Brazil
Source: PLoS One. 2023 Nov 17;18(11):e0291138. doi: 10.1371/journal.pone.0291138 (PMC10656034; doi:10.1371/journal.pone.0291138)
Supplement: S1 File — Presents the graphs of model predictions for the variables related to COVID-19 for the first experiment of this study. Each graph helps us understand the behavior of the predictions after training each model, tested for the 4-target series of this study: number of cases by publication date, number of cases by date of symptom onset, number of deaths by publication date, and number of deaths by date of death occurrence. However, without smoothing by ARIMA or post-processing on margin of error, just using deep learning models. (DOCX) [file pone.0291138.s001.docx]

**S1 File – Deep Learning Framework for epidemiological forecasting: A study on COVID-19 cases and deaths in the Amazon state of Pará, Brazil**

**Prediction plots of the trained models**

This supplementary material presents the graphs of model predictions for the variables related to COVID-19 for the first experiment of this study. Each graph helps us understand the behavior of the predictions after training each model, tested for the 4 target series of this study: number of cases by publication date, number of cases by date of symptom onset, number of deaths by publication date, and number of deaths by date of death occurrence. However, without smoothing by ARIMA or post-processing on margin of error, just using deep learning models. In the paper, we presents the final projection.

The graphs Fig S1.1 to Fig S1.5, projection of case series using the models selected by the proposed pipeline, compared to the actual data and projection 7 days ahead (showing last 30 days).


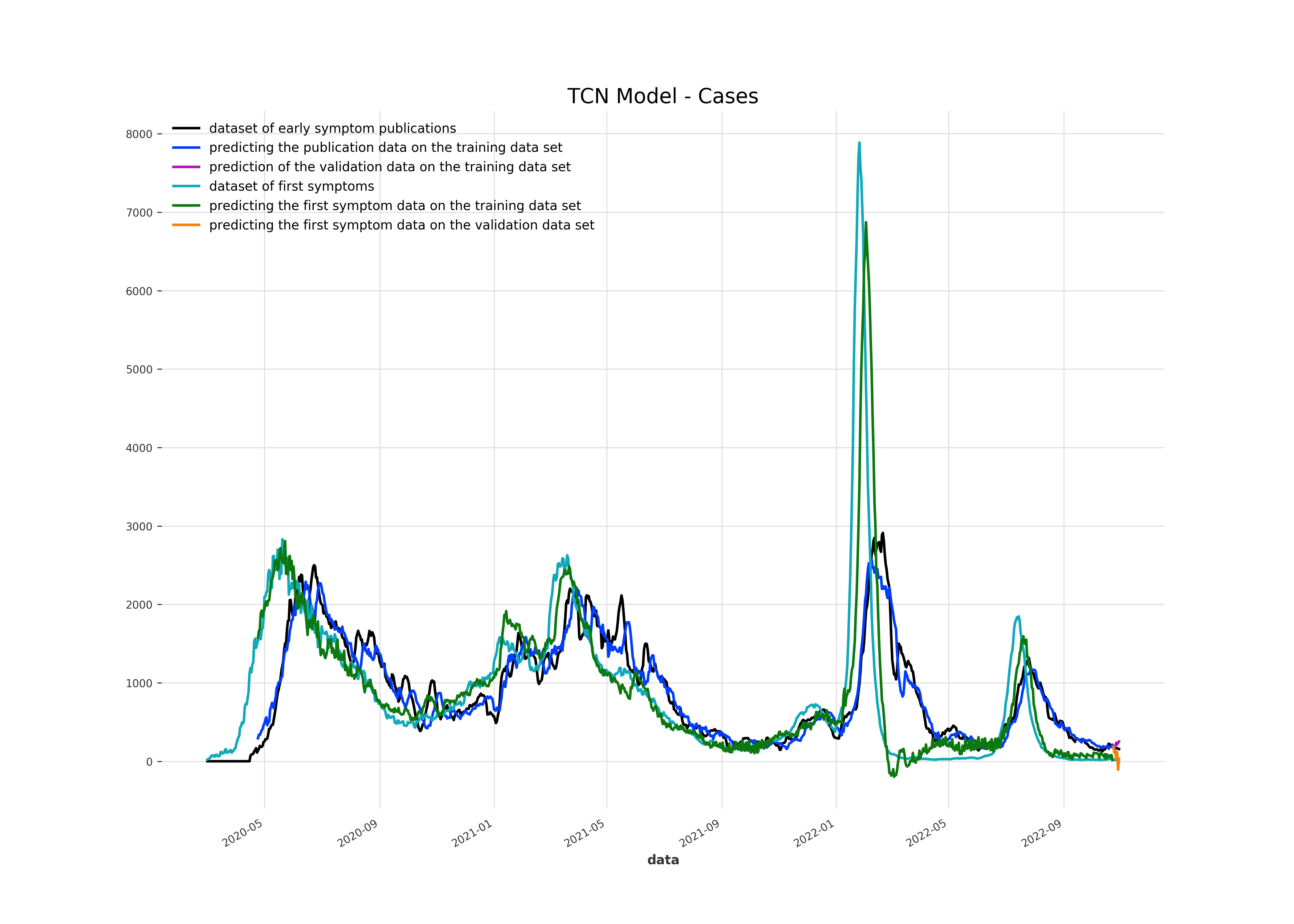

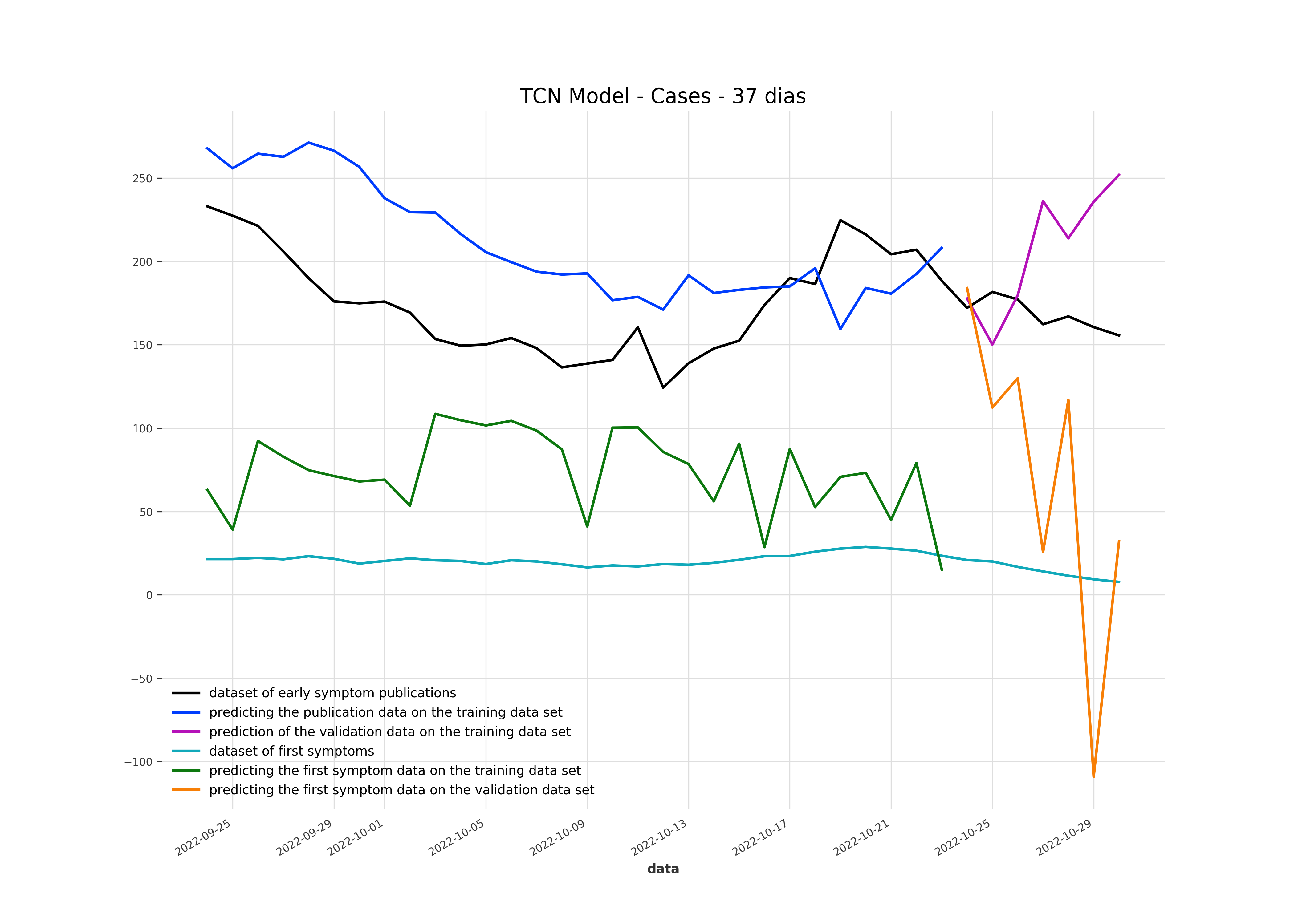


(A) (B)

**Fig S1.1**. Prediction plots on Training and Validation data on 10-31-2022 using TCN model for cases. (A) all days, (B) last 37 days.


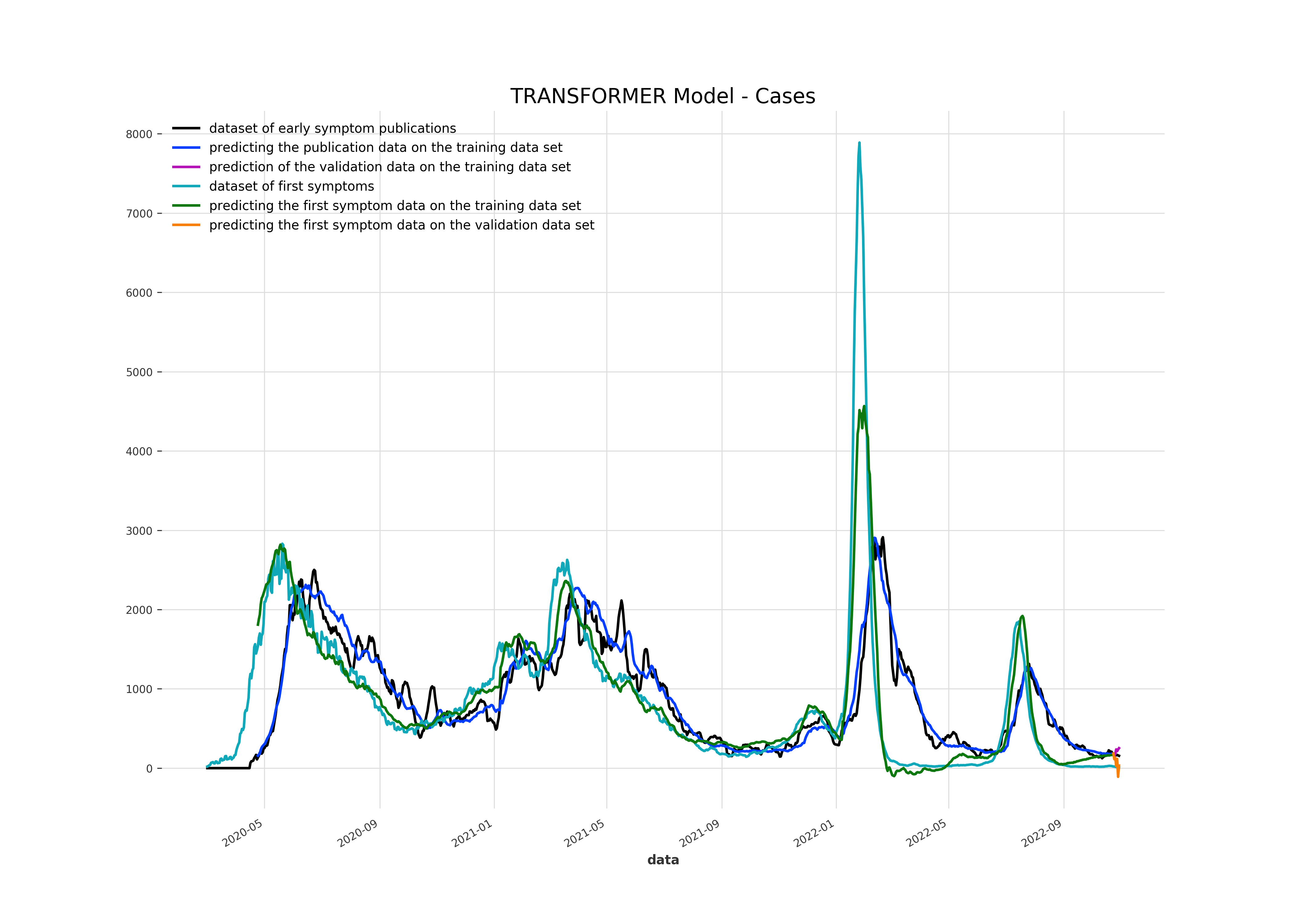

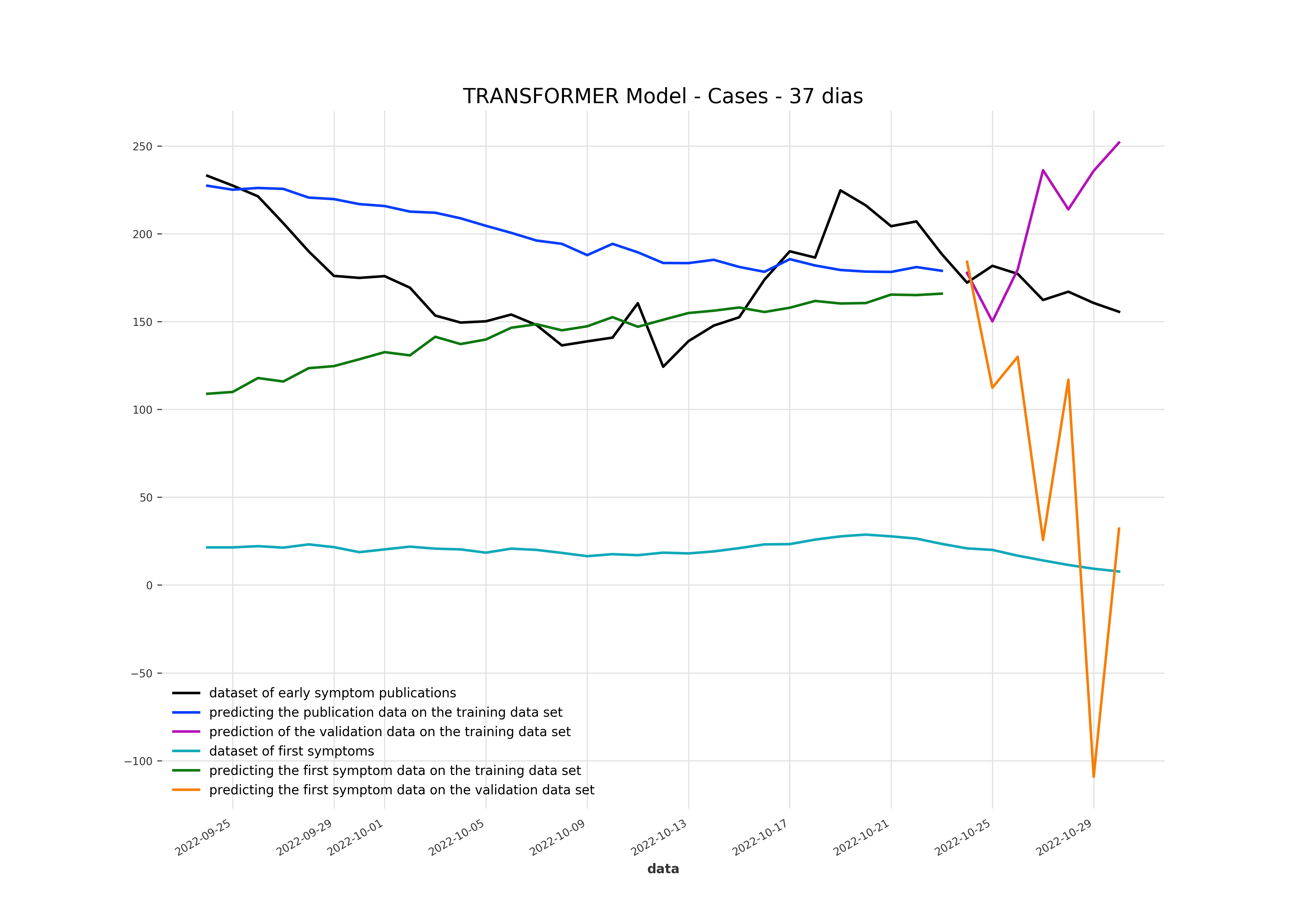


(A) (B)

**Fig S1.2**. Prediction plots on Training and Validation data on 10-31-2022 using TRANSFORMER model for cases. (A) all days, (B) last 37 days.


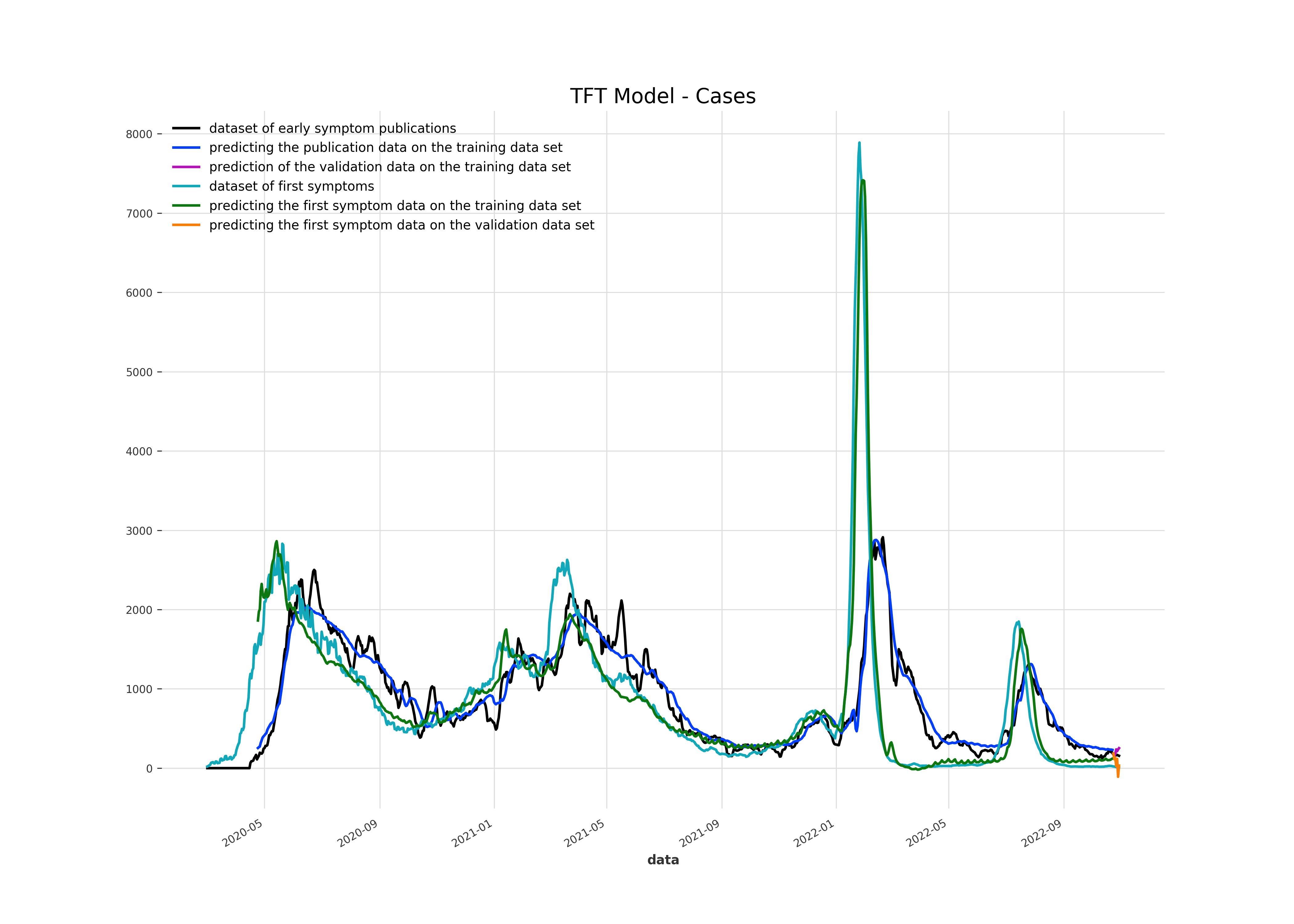

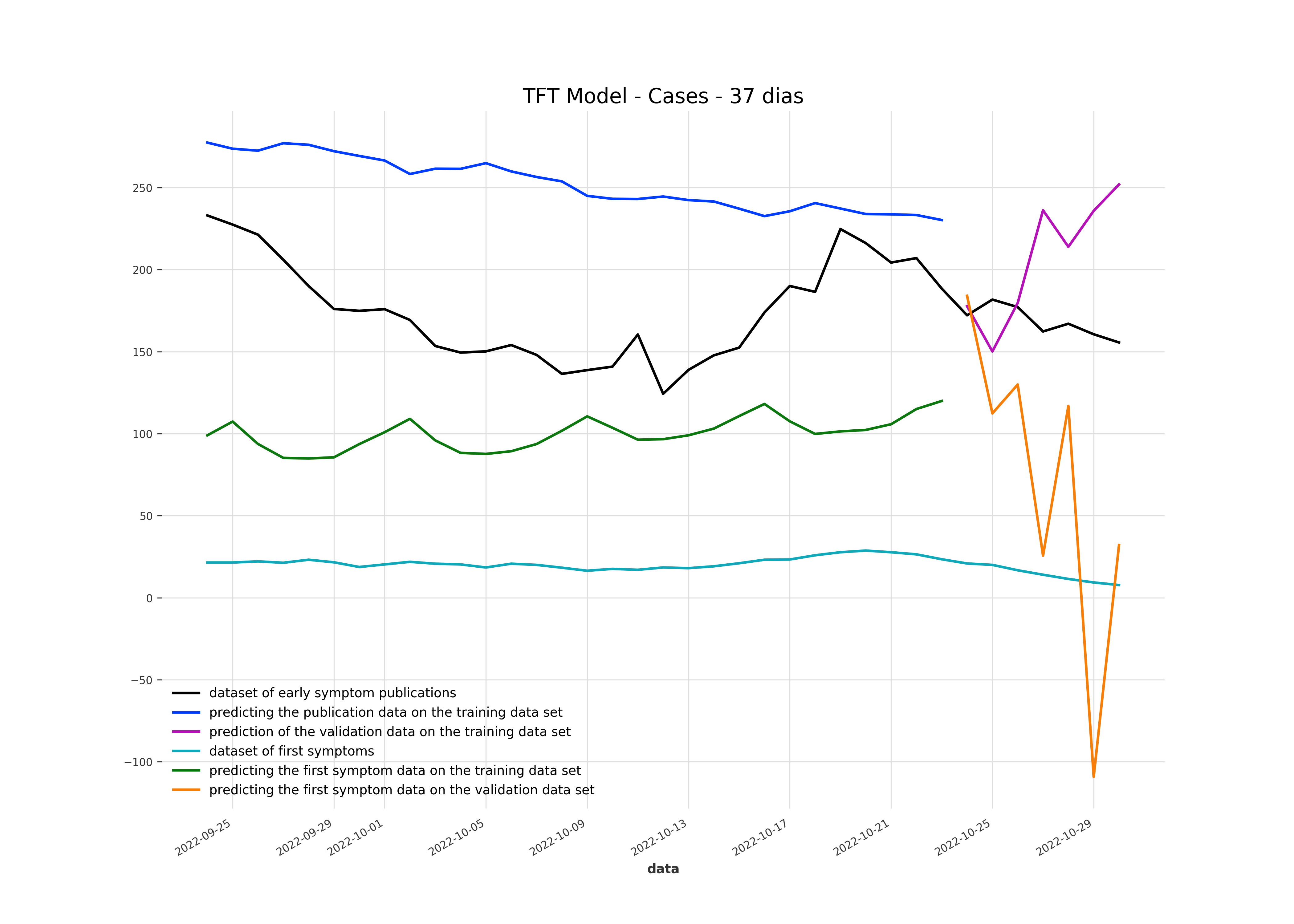


(A) (B)

**Fig S1.3**. Prediction plots on Training and Validation data on 10-31-2022 using TFT model for cases. (A) all days, (B) last 37 days.


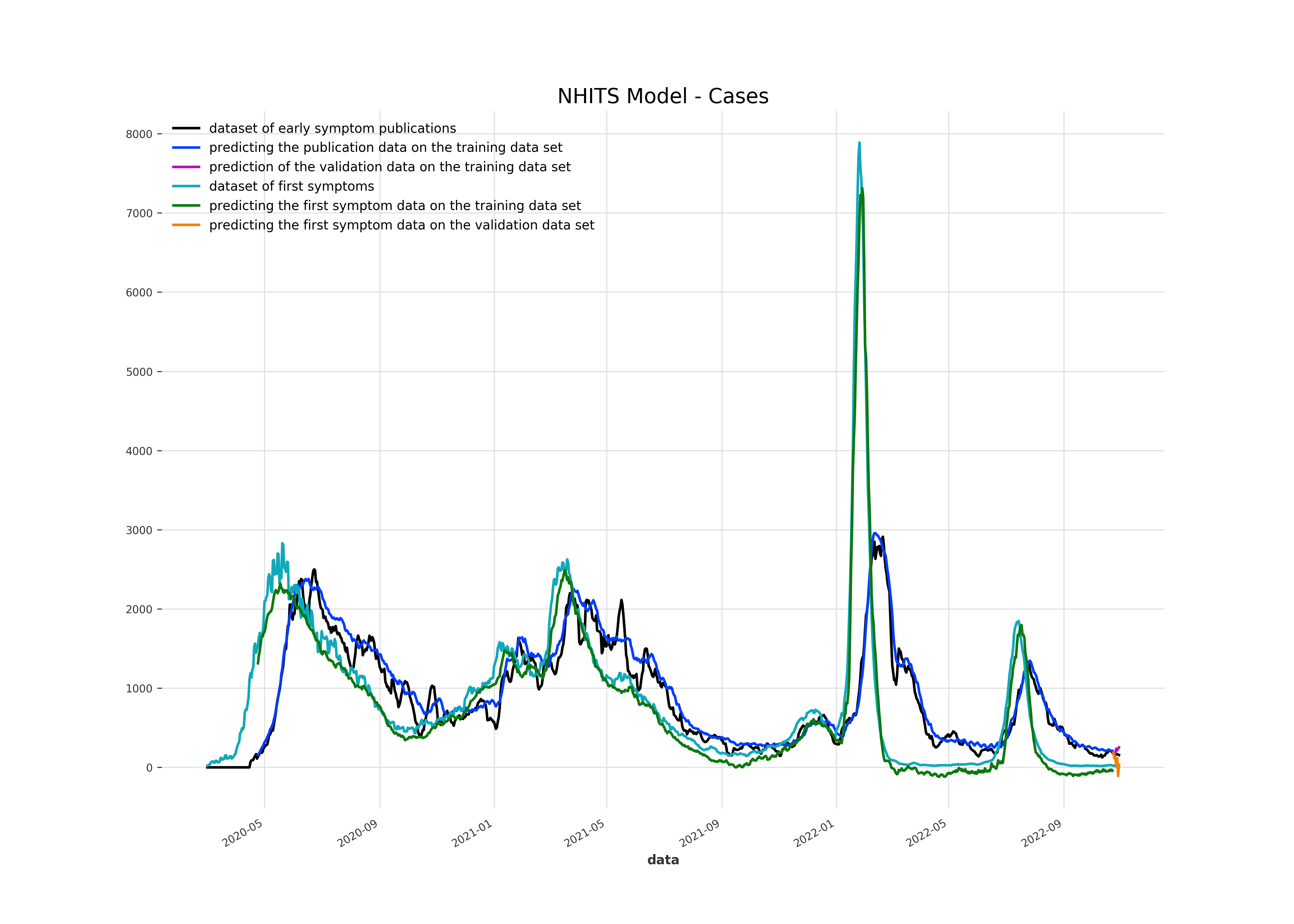

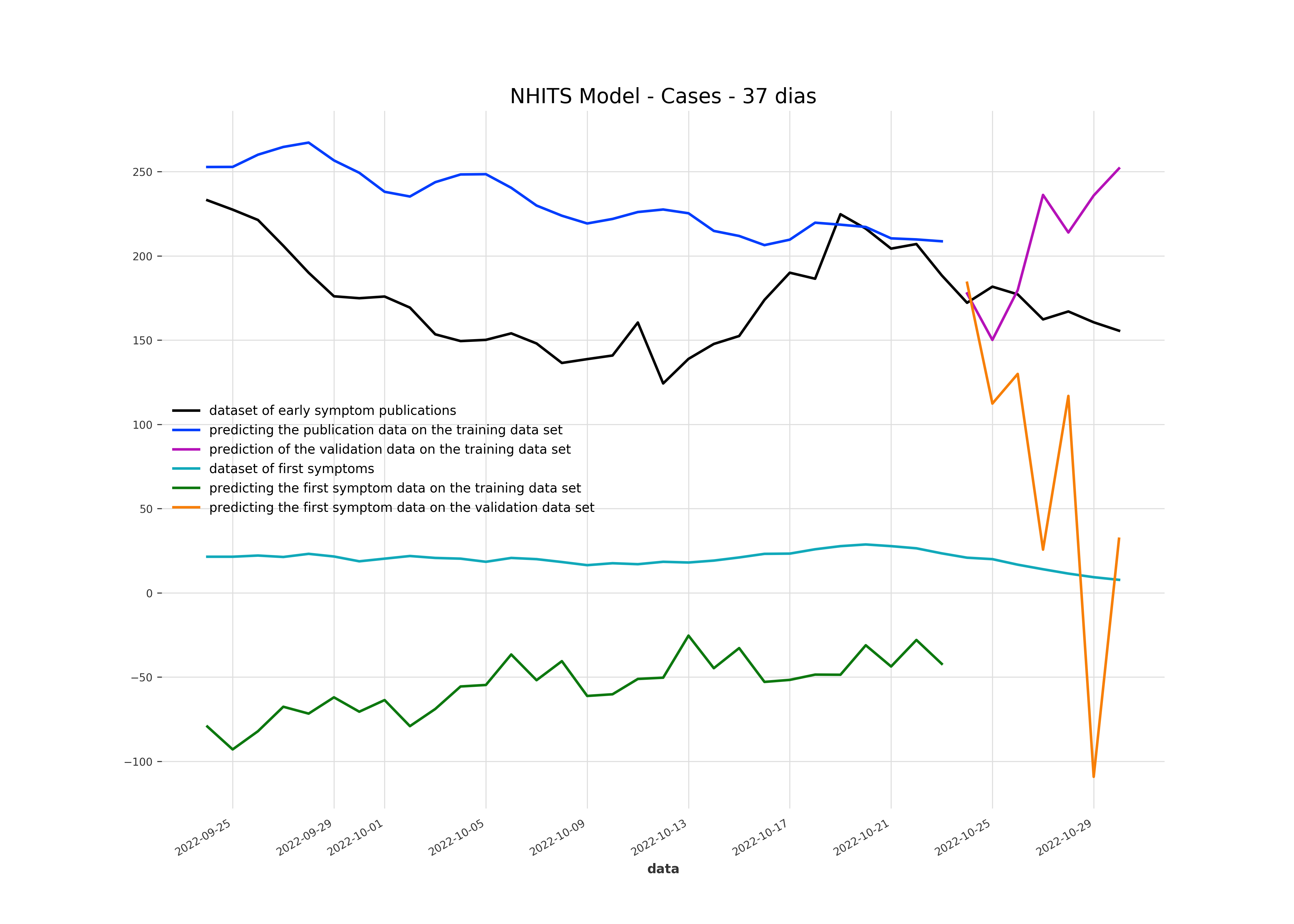


(A) (B)

**Fig S1.4**. Prediction plots on Training and Validation data on 10-31-2022 using NHITS model for cases. (A) all days, (B) last 37 days.


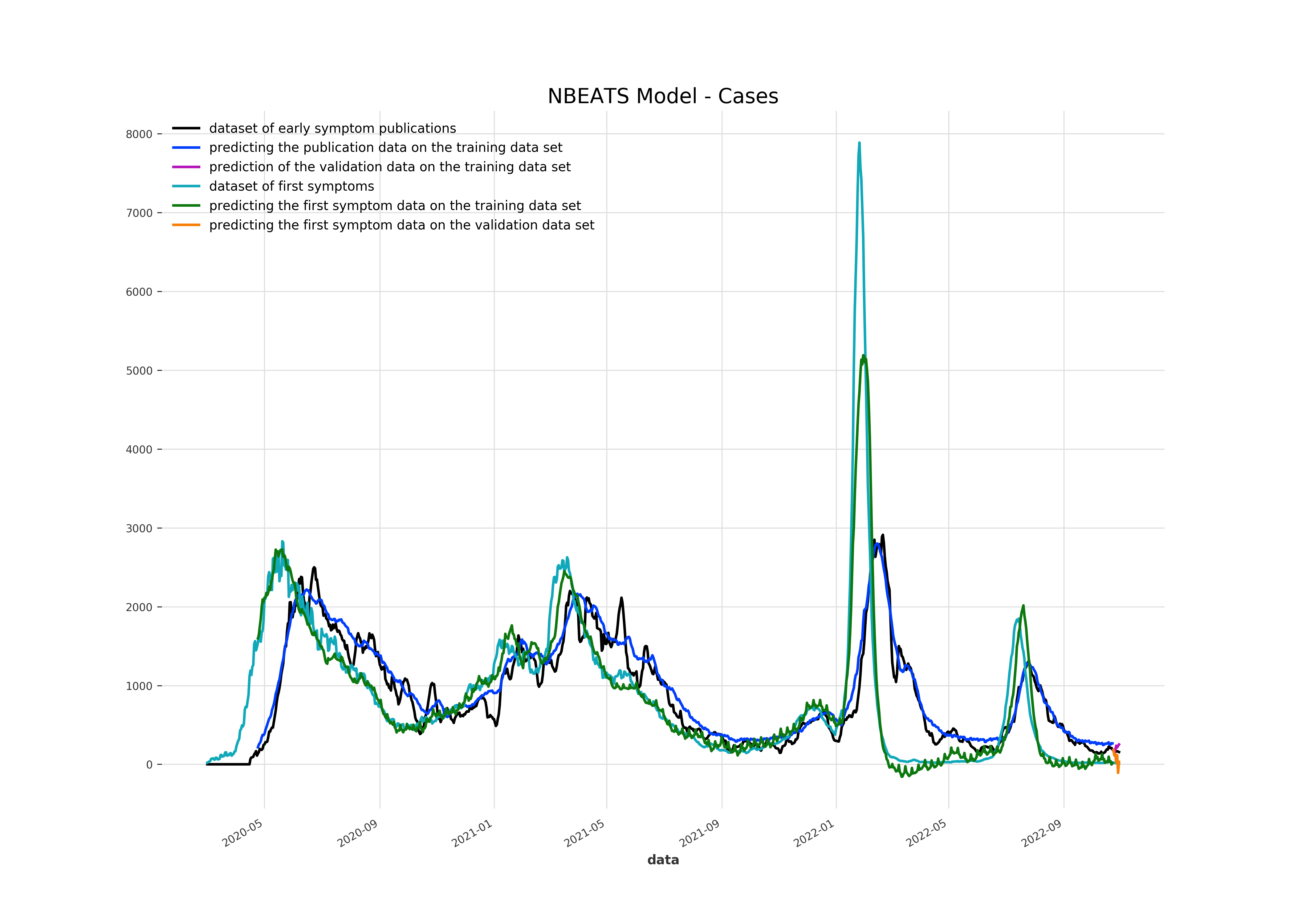

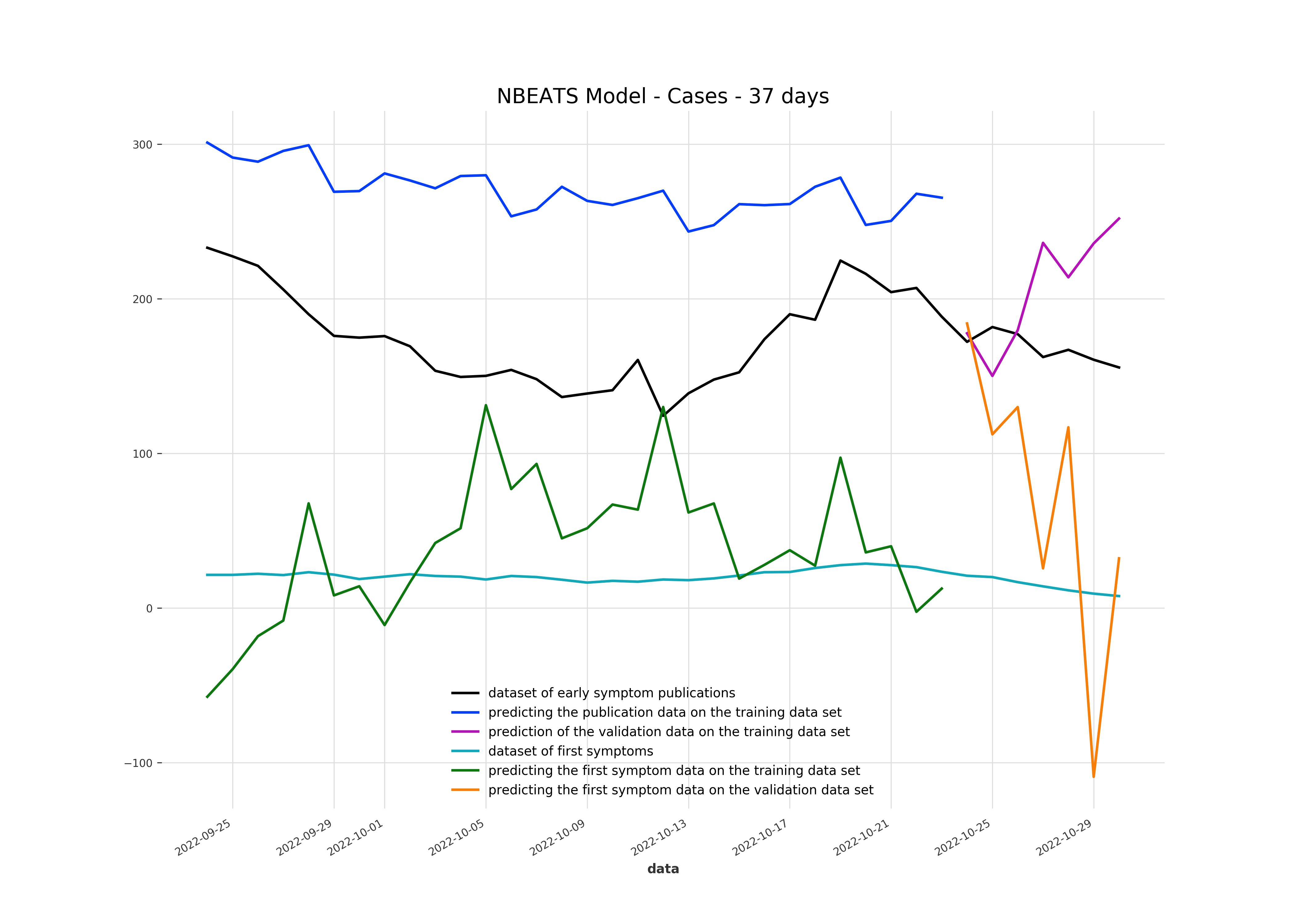


(A) (B)

**Fig S1.5**. Prediction plots on Training and Validation data on 10-31-2022 using NBEATS model for cases. (A) all days, (B) last 37 days.

The graphs Fig S1.6 to Fig S1.10, projection of the series referring to deaths using the models selected by the proposed pipeline, compared to the actual data and projection 7 days ahead (showing last 30 days).


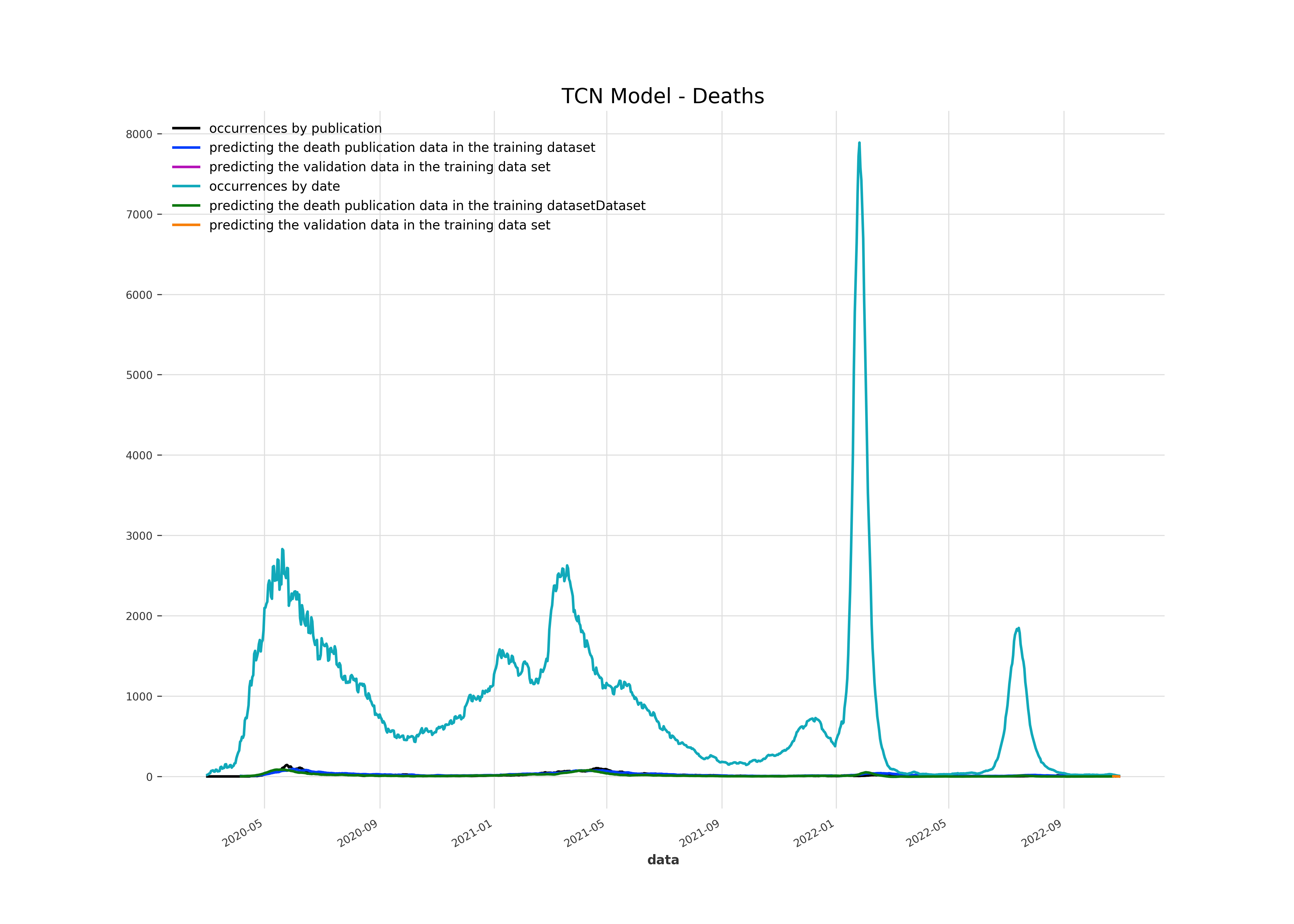

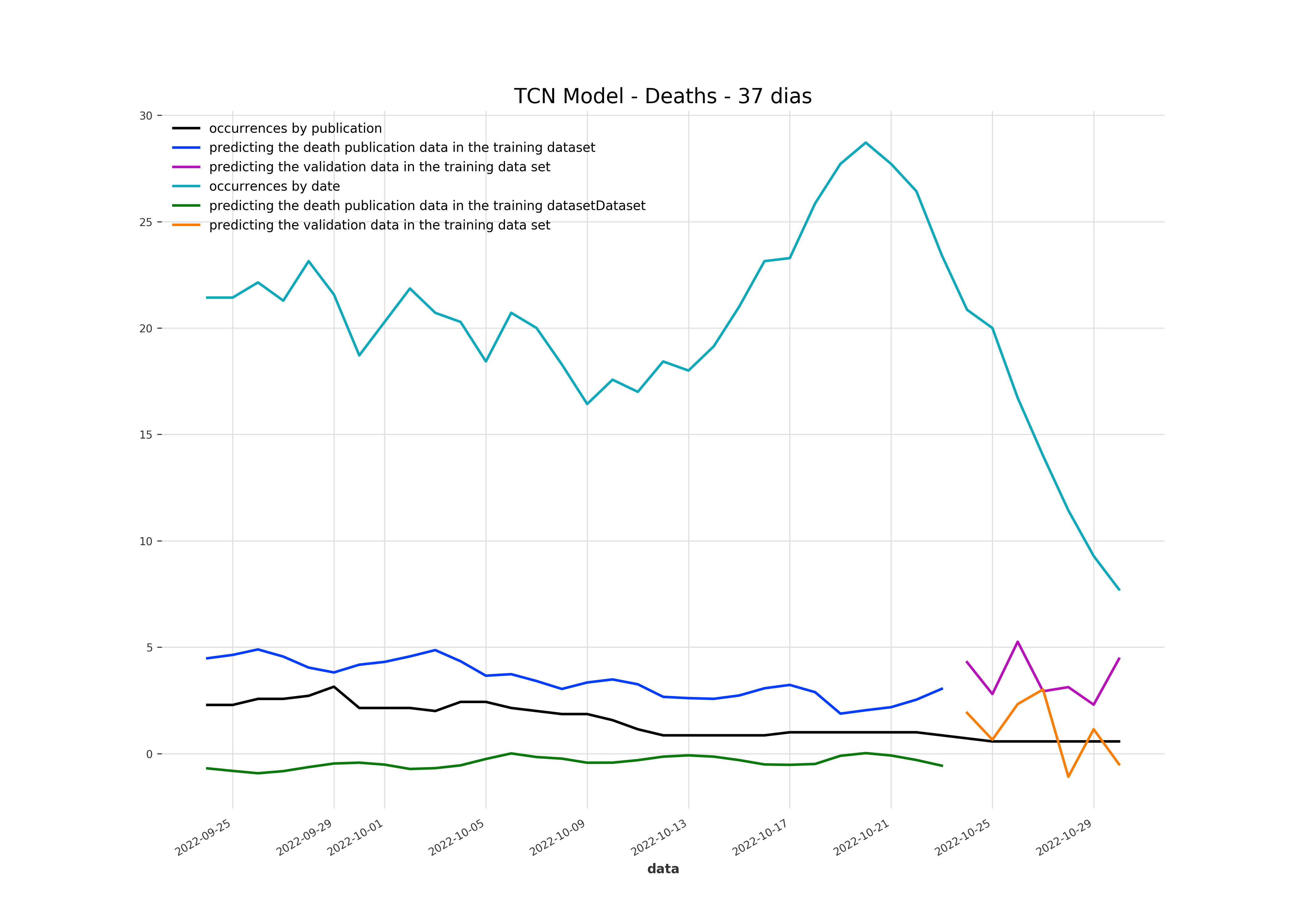


(A) (B)

**Fig S1.6**. Prediction plots on Training and Validation data on 10-31-2022 using TCN model for deaths. (A) all days, (B) last 37 days.


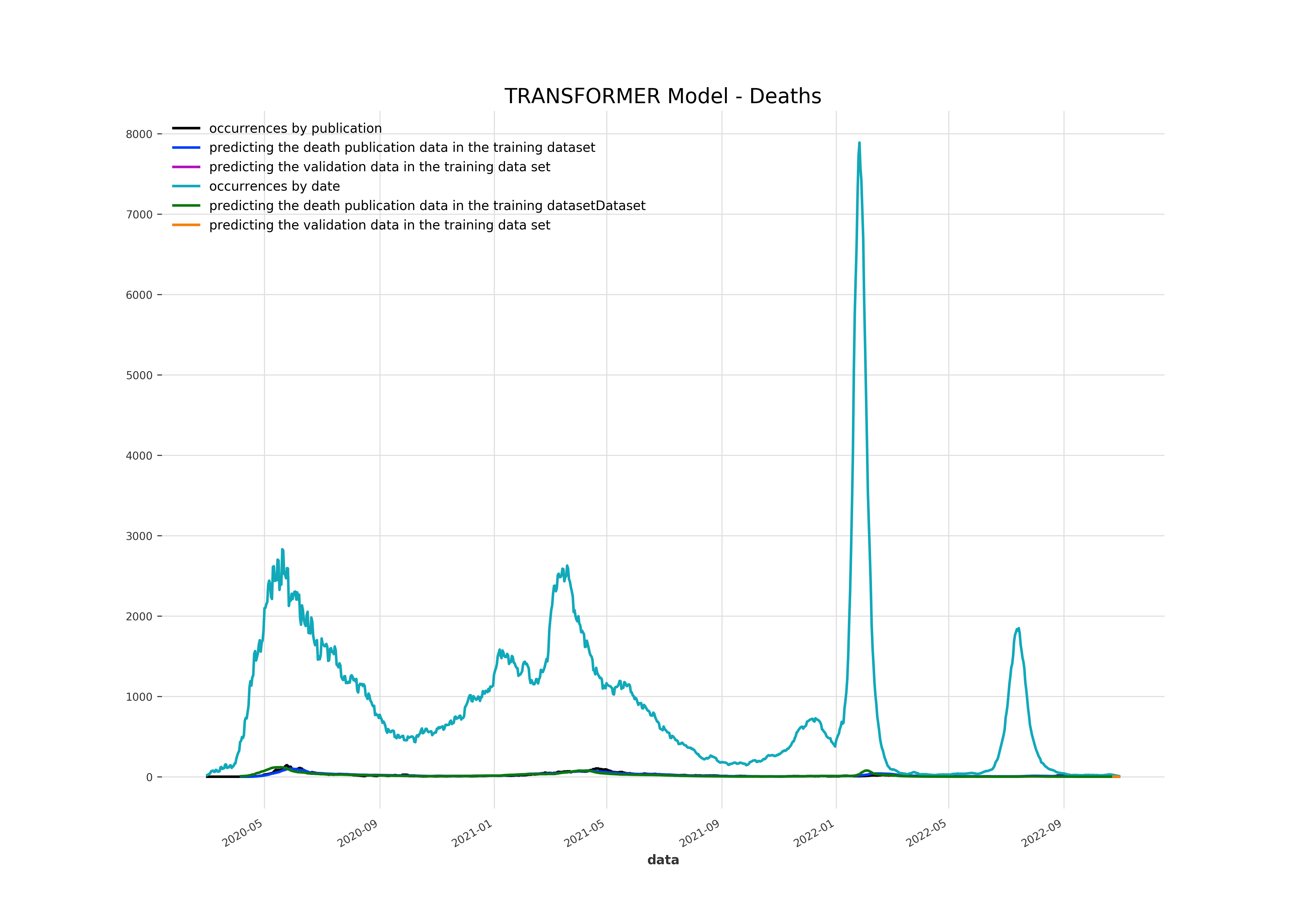

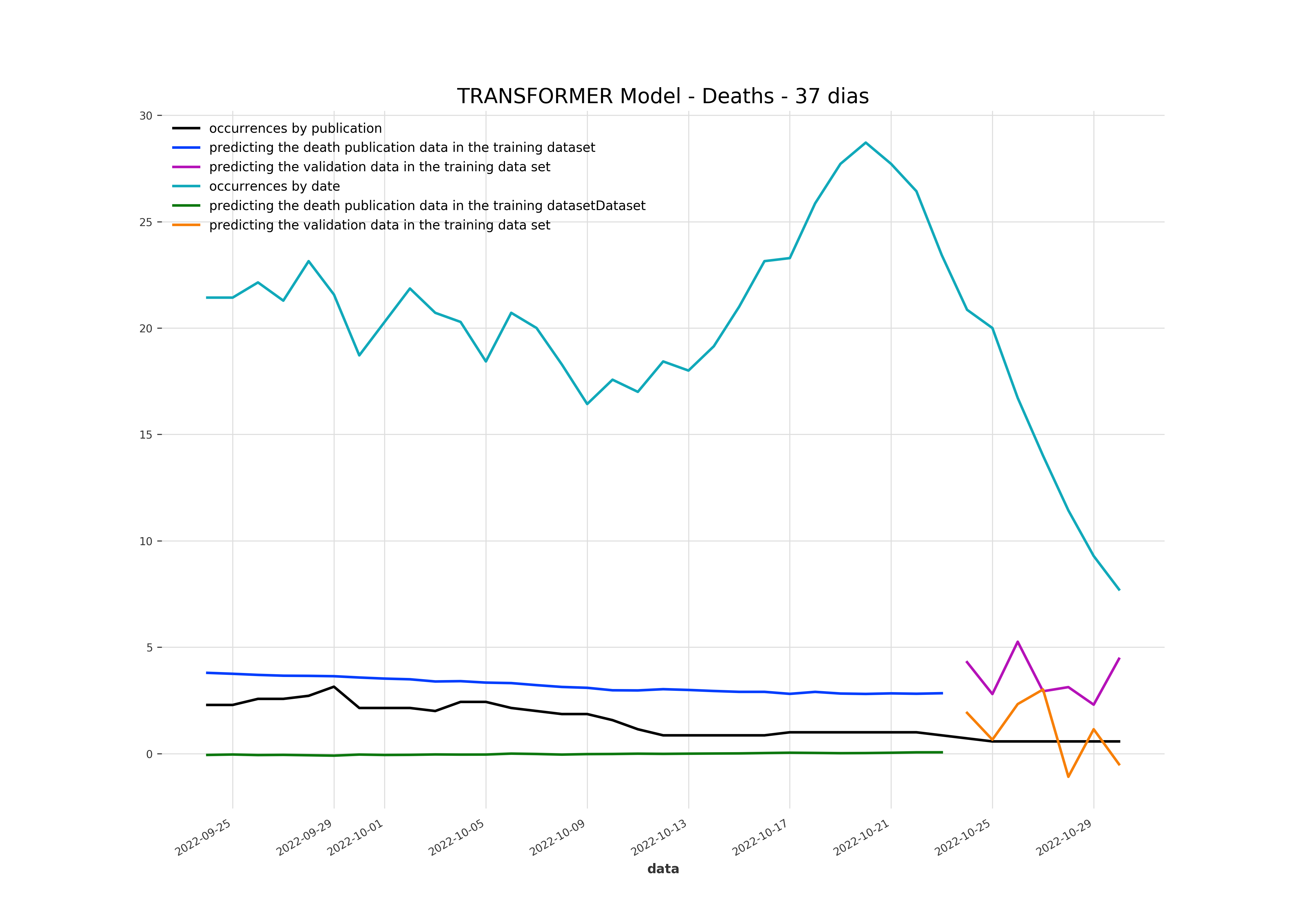


(A) (B)

**Fig S1.7**. Prediction plots on Training and Validation data on 10-31-2022 using TRANSFORMER model for deaths. (A) all days, (B) last 37 days.


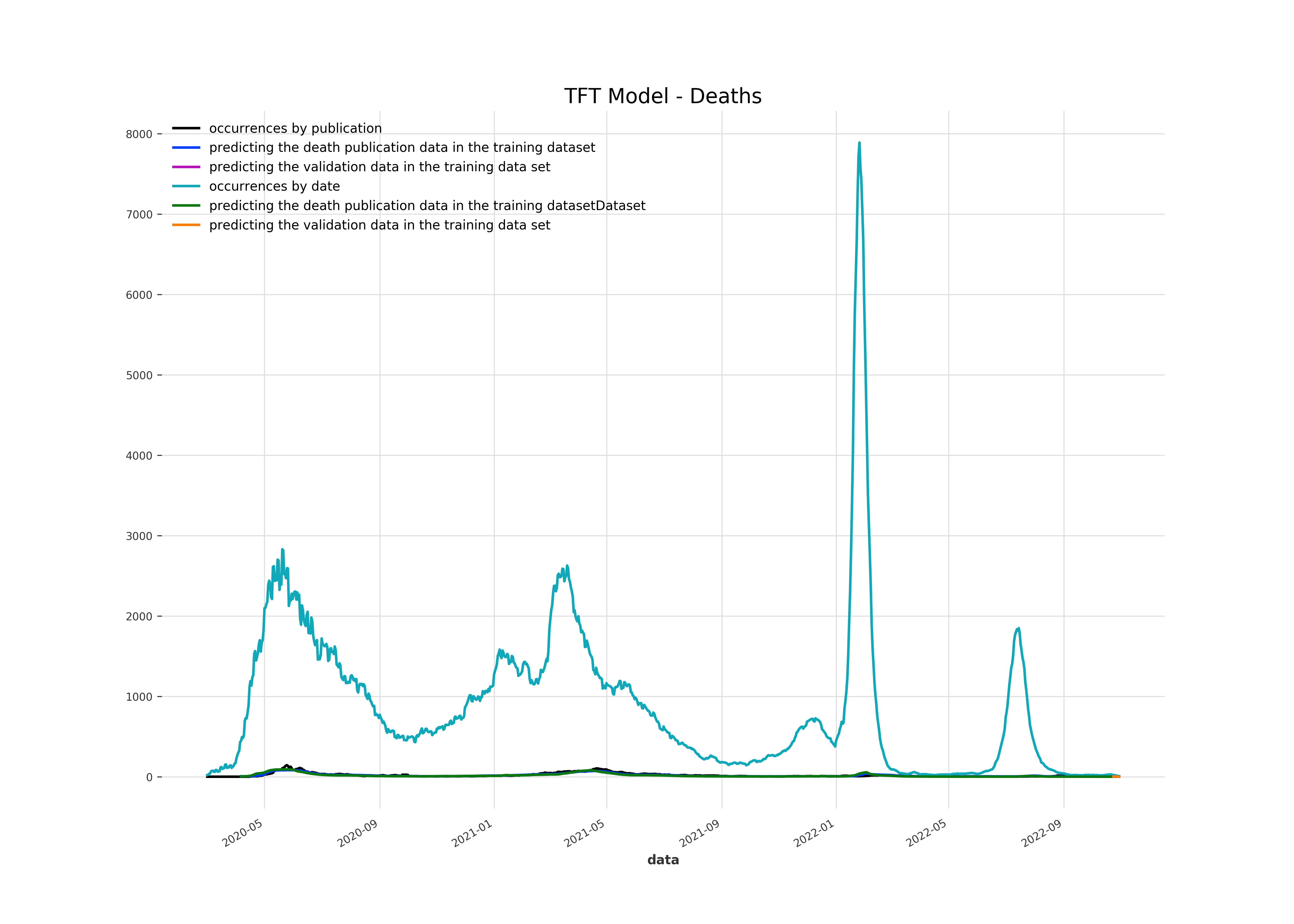

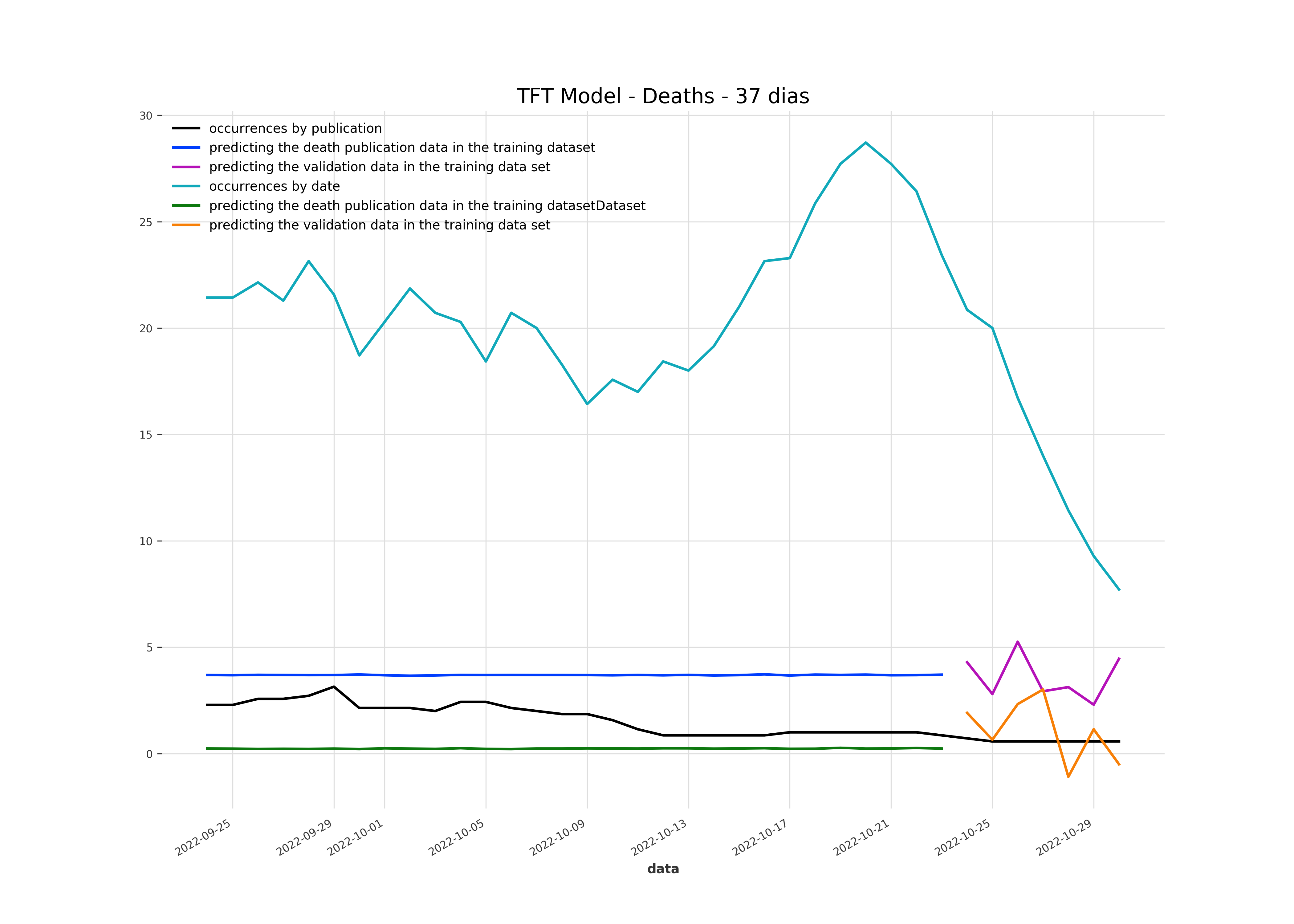


(A) (B)

**Fig S1.8**. Prediction plots on Training and Validation data on 10-31-2022 using TFT model for deaths. (A) all days, (B) last 37 days.


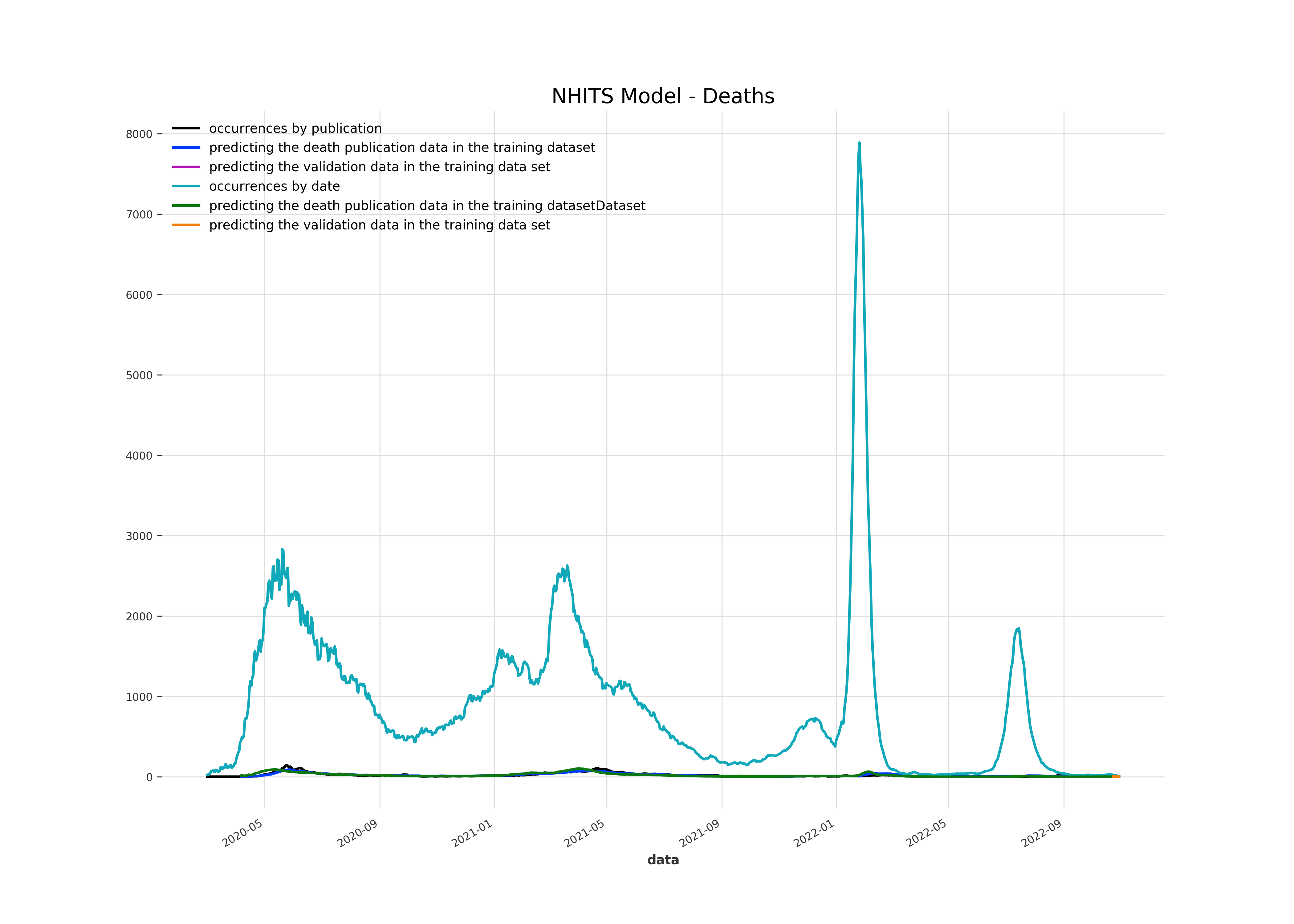

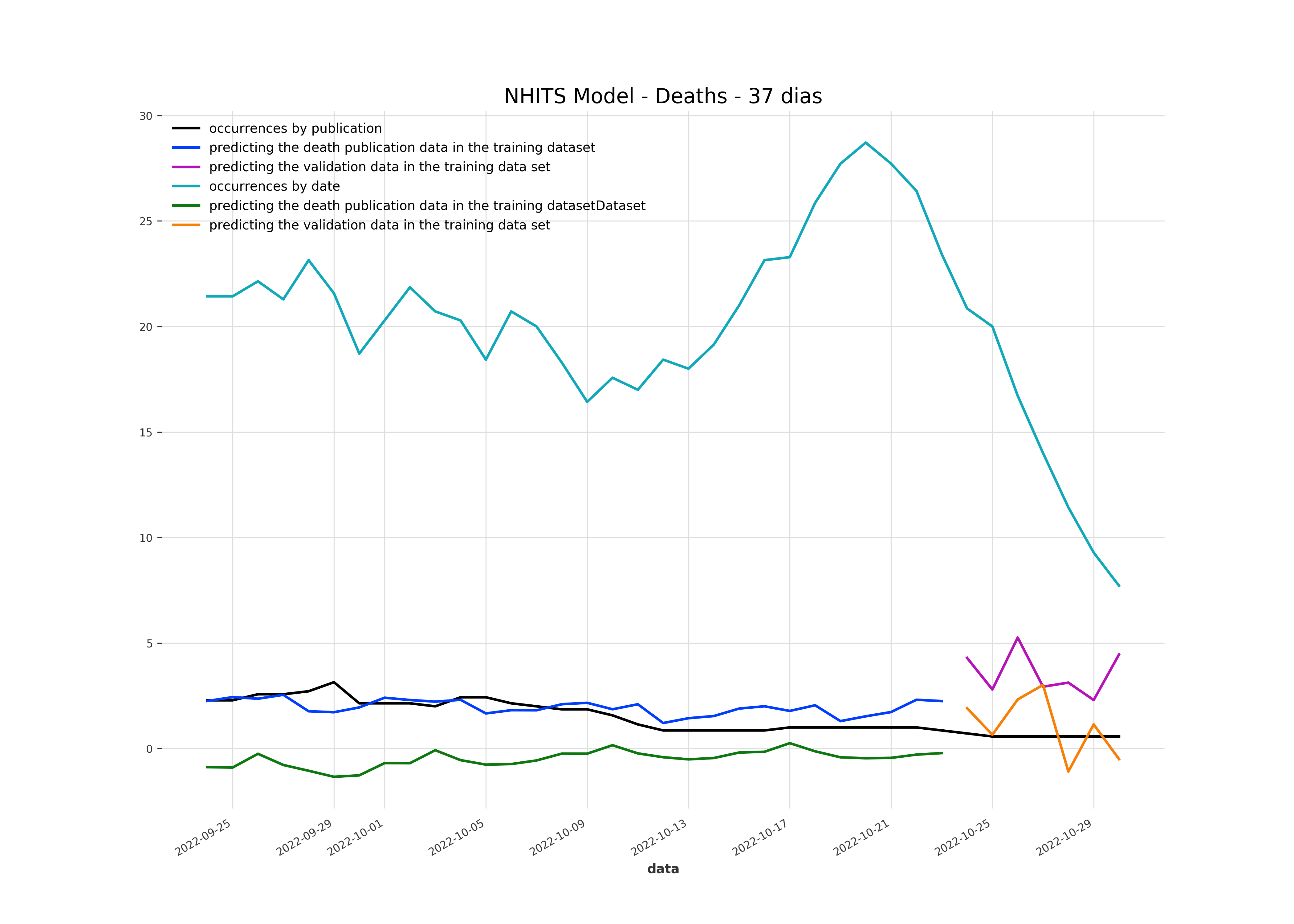


(A) (B)

**Fig S1.9**. Prediction plots on Training and Validation data on 10-31-2022 using TFT model for deaths. (A) all days, (B) last 37 days.


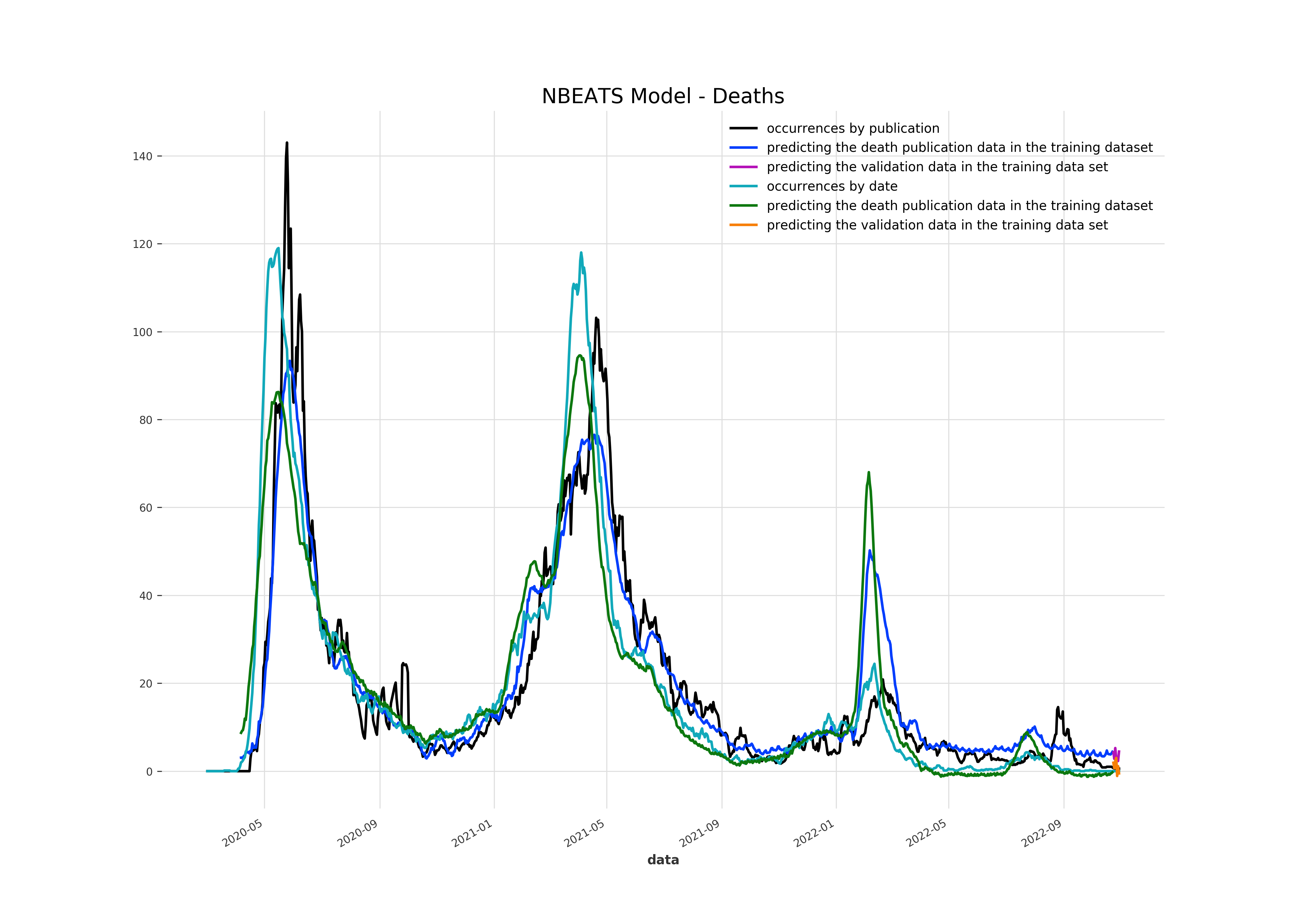

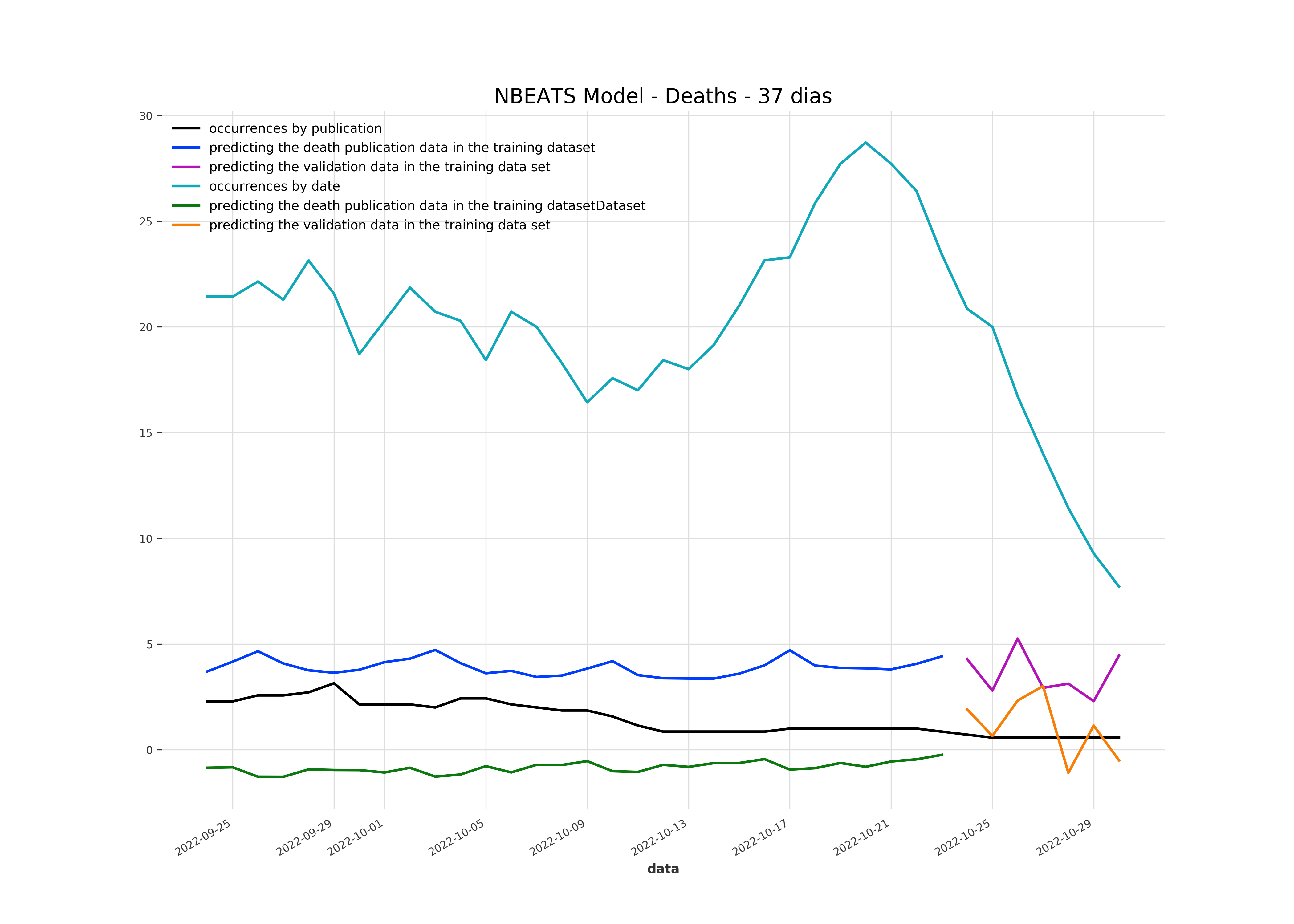


(A) (B)

**Fig S1.10**. Prediction plots on Training and Validation data on 10-31-2022 using NBEATS model for deaths. (A) all days, (B) last 37 days.

After selecting the best models, the entire series is used to forecast 7 days ahead, (but without smoothing by ARIMA or post-processing on margin of error) as shown in Fig S1.11 to Fig S1.14.


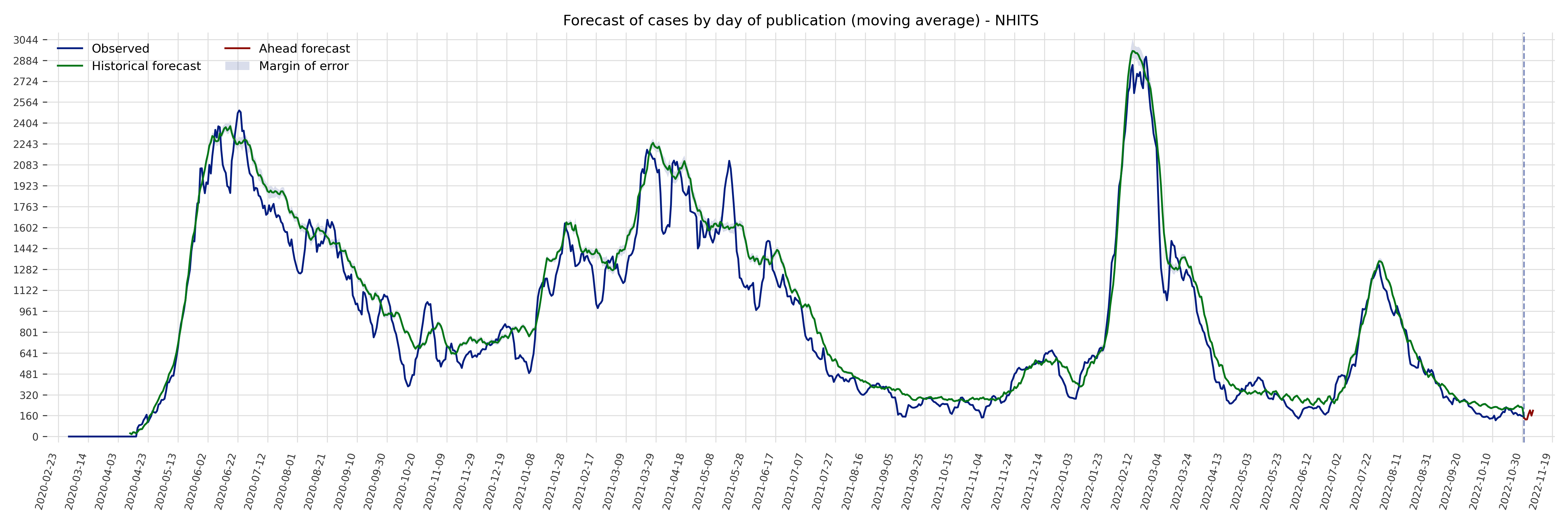


(A)


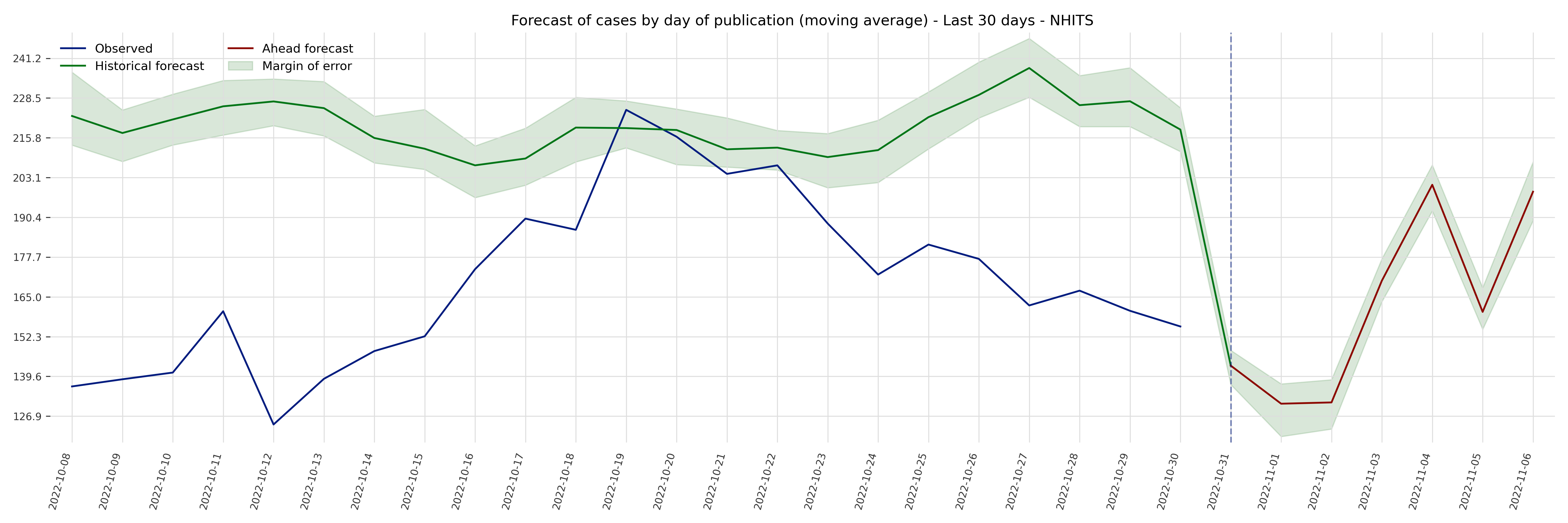


(B)

**Fig. S1.11.** Projection of the two case series using the best models selected by the proposed pipeline for cases by publication. A and B in with the actual data and projection 7 ahead (showing last 30 days).


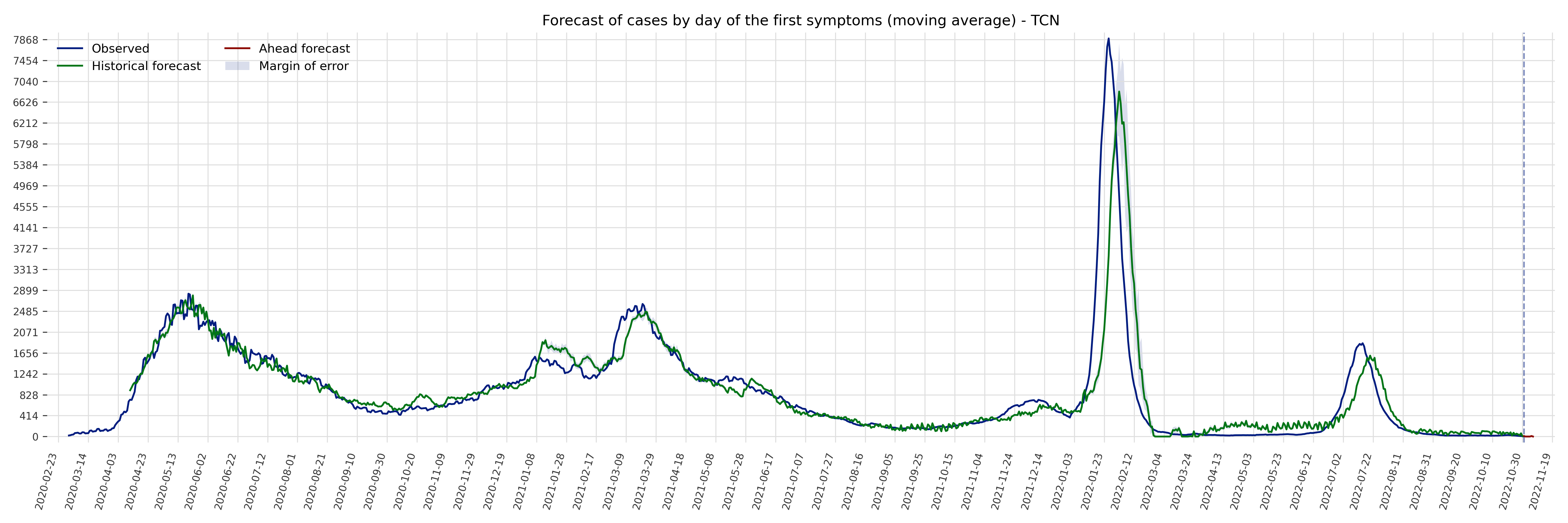


(A)


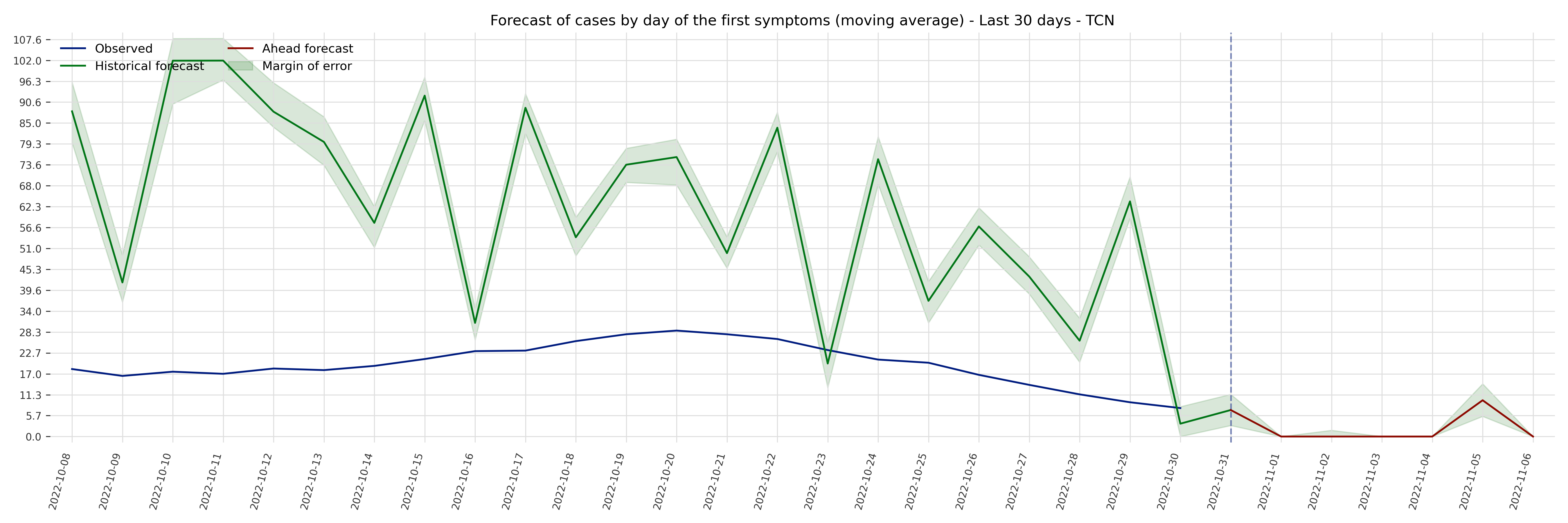


(B)

**Fig. S1.12.** Projection of the two case series using the best models selected by the proposed pipeline for cases by days of symptomns. A and B in with the actual data and projection 7 ahead (showing last 30 days).


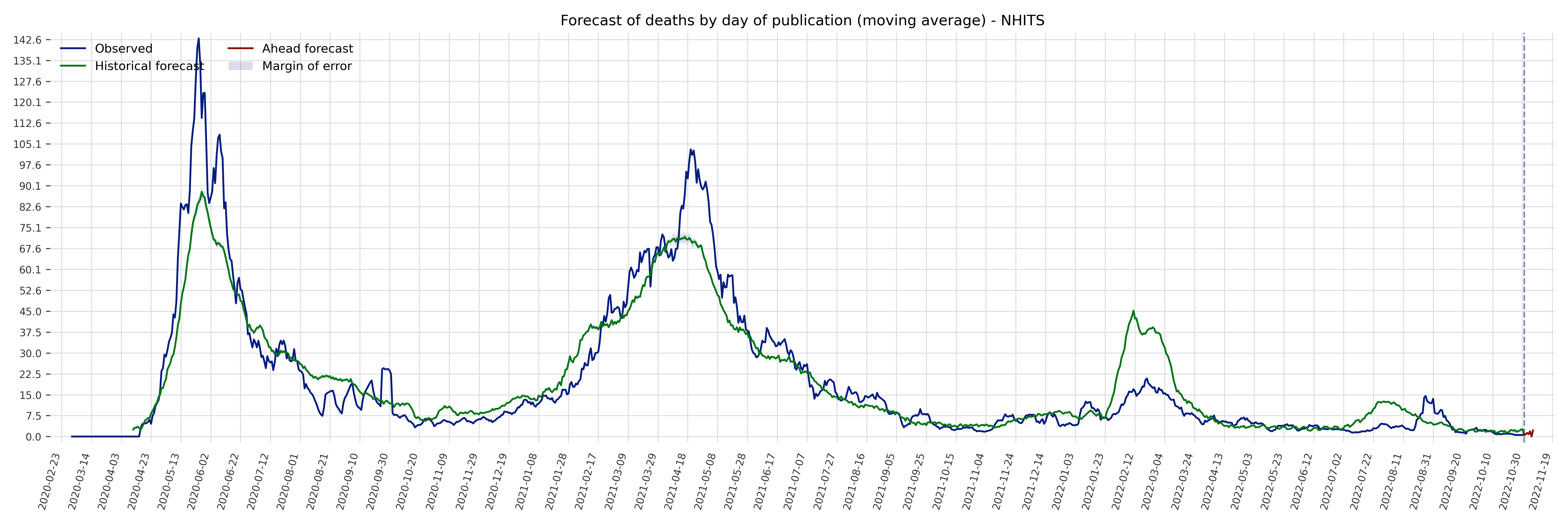


(A)


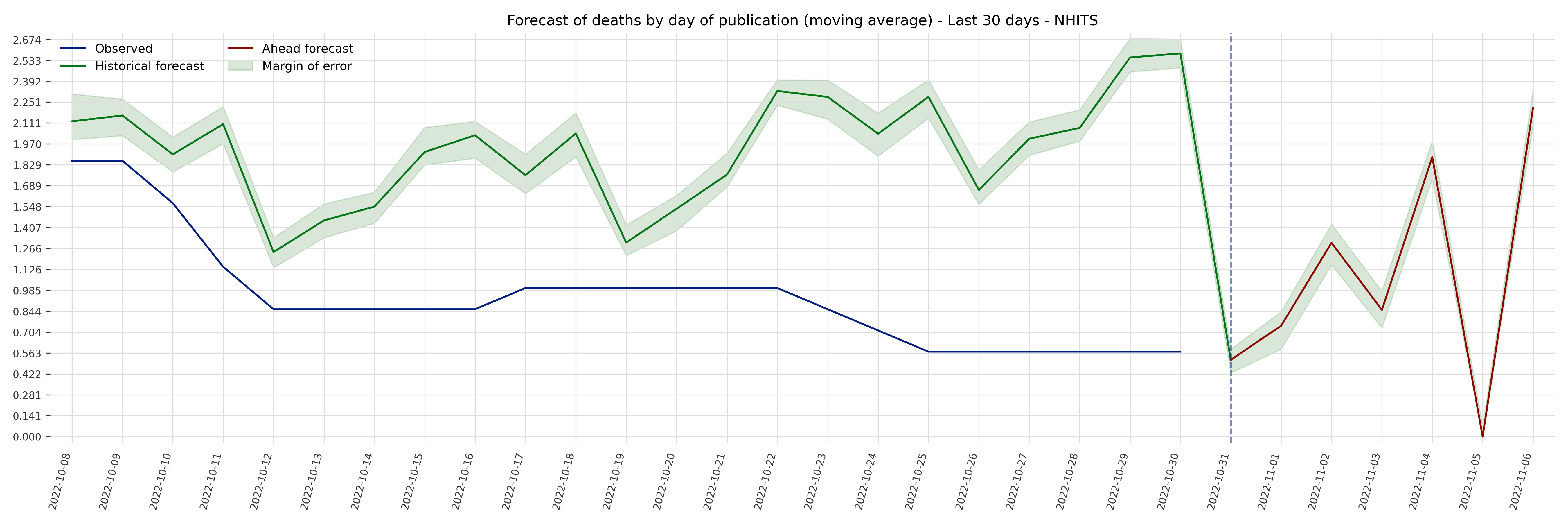


(B)

**Fig. S1.13.** Projection of the 2 series regarding deaths using the best models selected by the proposed pipeline for deaths by day of publication. A and B in with the actual data and projection 7 ahead (showing last 30 days).


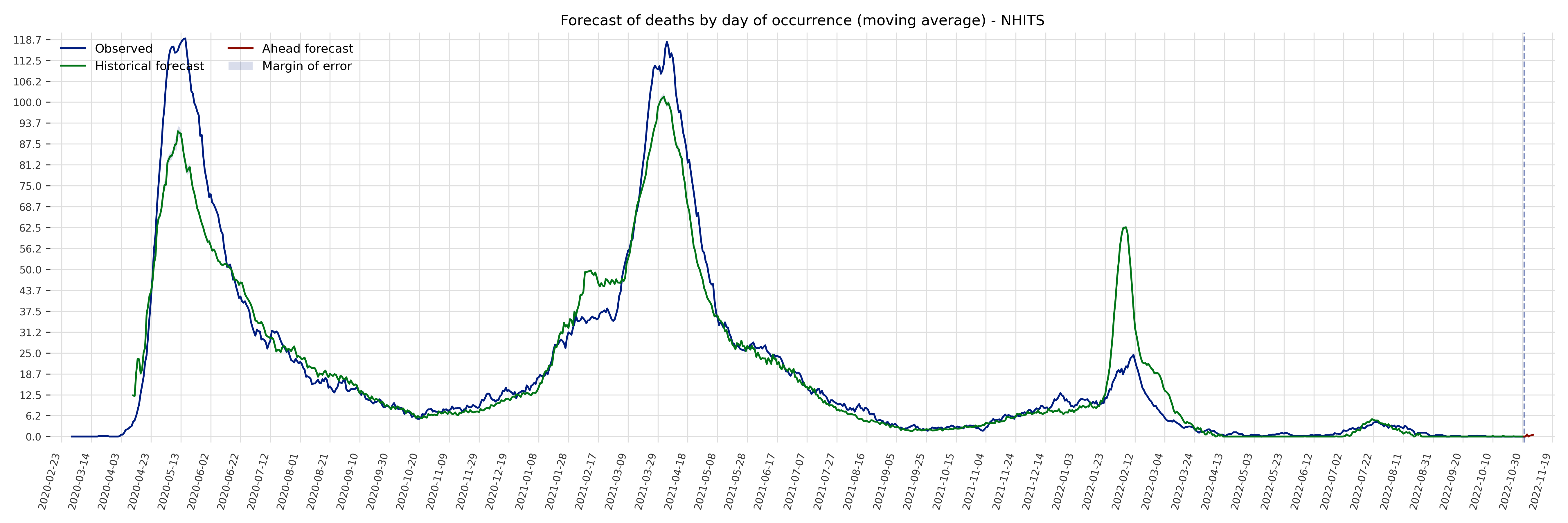


(A)


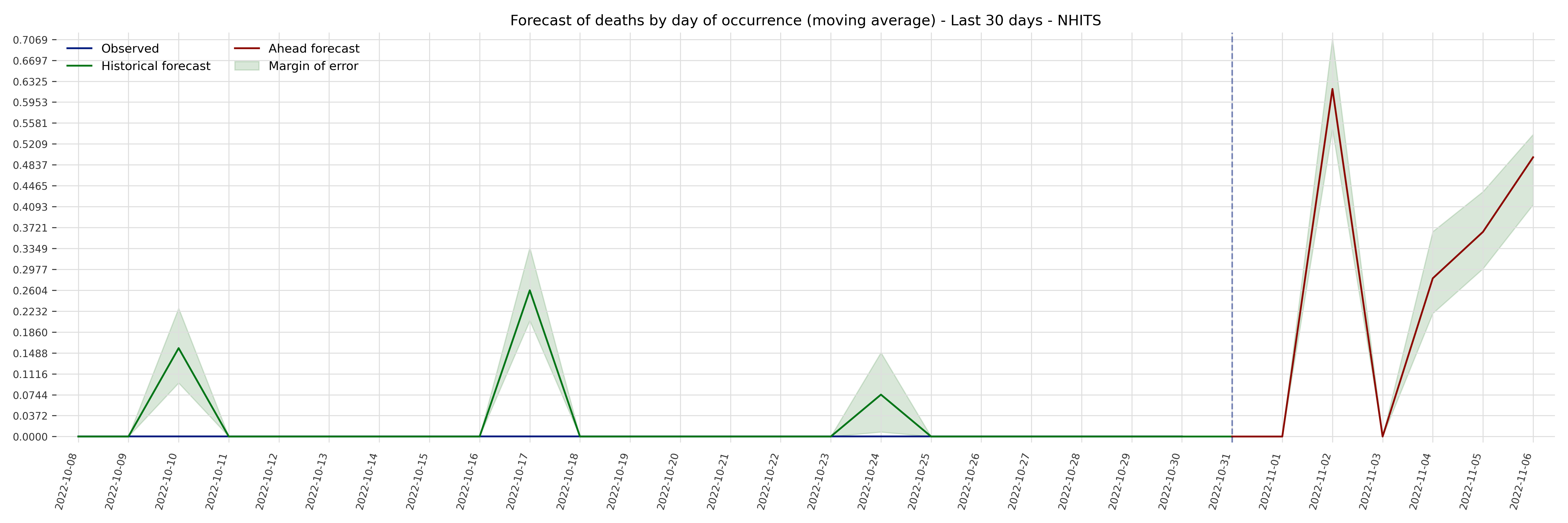


(B)

**Fig. S1.14.** Projection of the 2 series regarding deaths using the best models selected by the proposed pipeline for deaths by day of occurrence. A and B in with the actual data and projection 7 ahead (showing last 30 days).
