# Supplementary material for: Deep learning framework for epidemiological forecasting: A study on COVID-19 cases and deaths in the Amazon state of Pará, Brazil
Source: PLoS One. 2023 Nov 17;18(11):e0291138. doi: 10.1371/journal.pone.0291138 (PMC10656034; doi:10.1371/journal.pone.0291138)
Supplement: S2 File — Presents the graphs for the analysis of the residuals of the best projection models of variables related to COVID-19. Each graph helps us to understand the behavior of the projections and the best models selected for the 4-target series of the present study: number of cases by date of publication, number of cases by date of onset of symptoms, number of deaths by date of publication, and number of deaths by date of death occurrence. (DOCX) [file pone.0291138.s002.docx]

**S2 File – Deep Learning Framework for epidemiological forecasting: A study on COVID-19 cases and deaths in the Amazon state of Pará, Brazil**

**Residual analysis graphs of the best models**

This supplementary material presents the graphs for the analysis of the residuals of the best projection models of variables related to COVID-19. Each graph helps us to understand the behavior of the projections and the best models selected for the 4 target series of the present study: number of cases by date of publication, number of cases by date of onset of symptoms, number of deaths by date of publication, and number of deaths by date of death occurrence. The plots display the values of the residuals, the distribution of the residuals, the ACF plot (Autocorrelation Function), and the Q-Q Plot (Quatile-Quantile).

The graphics were chosen with the intention of identifying and avoiding phenomena such as the models overfitting the training data and learning by chance, leading to a bad model generalization. Through the identification of the regression and the type of distribution, it was possible to identify the model in which the residual errors were minimized and from which the best statistical results were obtained, providing a visual validation of the performance of the model and the errors that were generated. The regression chart was important to monitor the performance of the model over time and the type of error that was generated, thus making it possible to identify and make the necessary adjustments to minimize residual errors.

In the regression chart, we can identify the existence of some inclination associated with the residual values of the model, bearing in mind that the closer the angular coefficient is to 0, the better. This graph is important to identify the biases of the measurement errors generated by the model. It also provides the possibility of analyzing the residual trend evolution over time and its behavior. If the residuals begin to show both an increasing and a decreasing propensity, this means that the model can be showing a biased reaction and the parameters must be adjusted for correction, considering that the most acceptable errors must present a random distribution.

The autocorrelation plot indicates how the sign of the residuals changes over time. Like the graph mentioned above, this one is also intended to validate and verify the residual evolution. This chart, unlike the others, does not measure the frequency of residuals, only their correlation over time. Its use is indicated when you want to analyze the relationship between current and future values in time series to visualize temporal variation, identifying seasonality and other patterns.

The identification of the distribution of residuals is another method of guaranteeing that the data will be presenting satisfactory residual values for the forecasts generated. According to the central limit theorem, as the sample size increases, the distribution approaches a normal one. The ideal normal distribution considered that the errors would be normally distributed, where their mean, median, and mode are approximately 0, as is expected from this type of distribution. The presence of these characteristics would reveal a good performance of the tested model, where the residual values are close to 0 with random and symmetric upper and lower variations. One of the most popular distribution visualization charts is the histogram. Its popularization can be attributed to its easy reading and interpretation. Another graph used for the purpose of identifying characteristics of the residual distribution was the Q-Q plot, considering that some of its advantages include that the size of the samples to be compared does not need to be of equal sizes, as well as the possibility of testing and identifying specific anomalies in the samples, scale irregularities, symmetry change and the presence of outliers.

The graphs, Fig S2.1 to Fig S2.8, show the residual analysis of the projections on the training and validation data using the models selected by the proposed framework for the cases and deaths series.

**
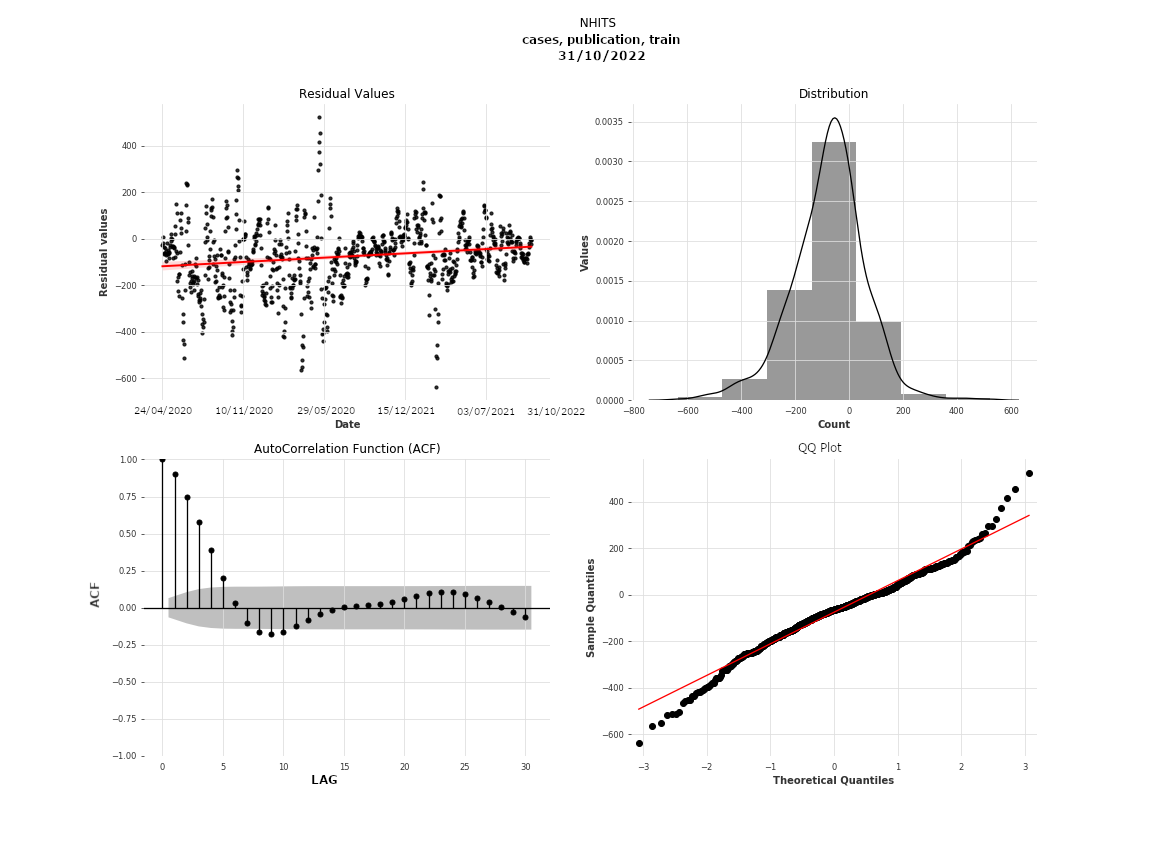
Fig S2.1.** Graphics of the residual analysis of the NHITS model, for the projection based on the training data, from the Publication of Cases series generated on 31-10-2022.


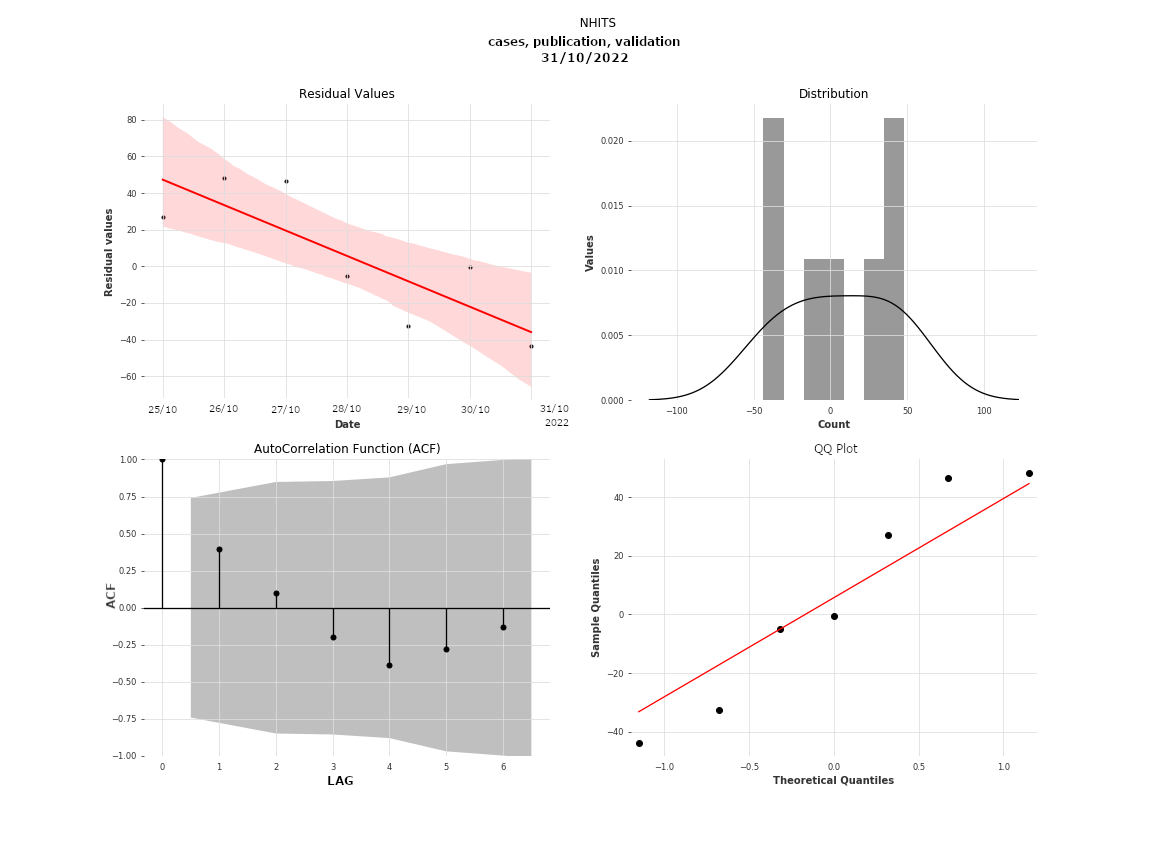
**Fig S2.2**. Graphics of the residual analysis of the NHITS model, for the projection based on the validation data, from the Publication of Cases series generated on 31-10-2022.


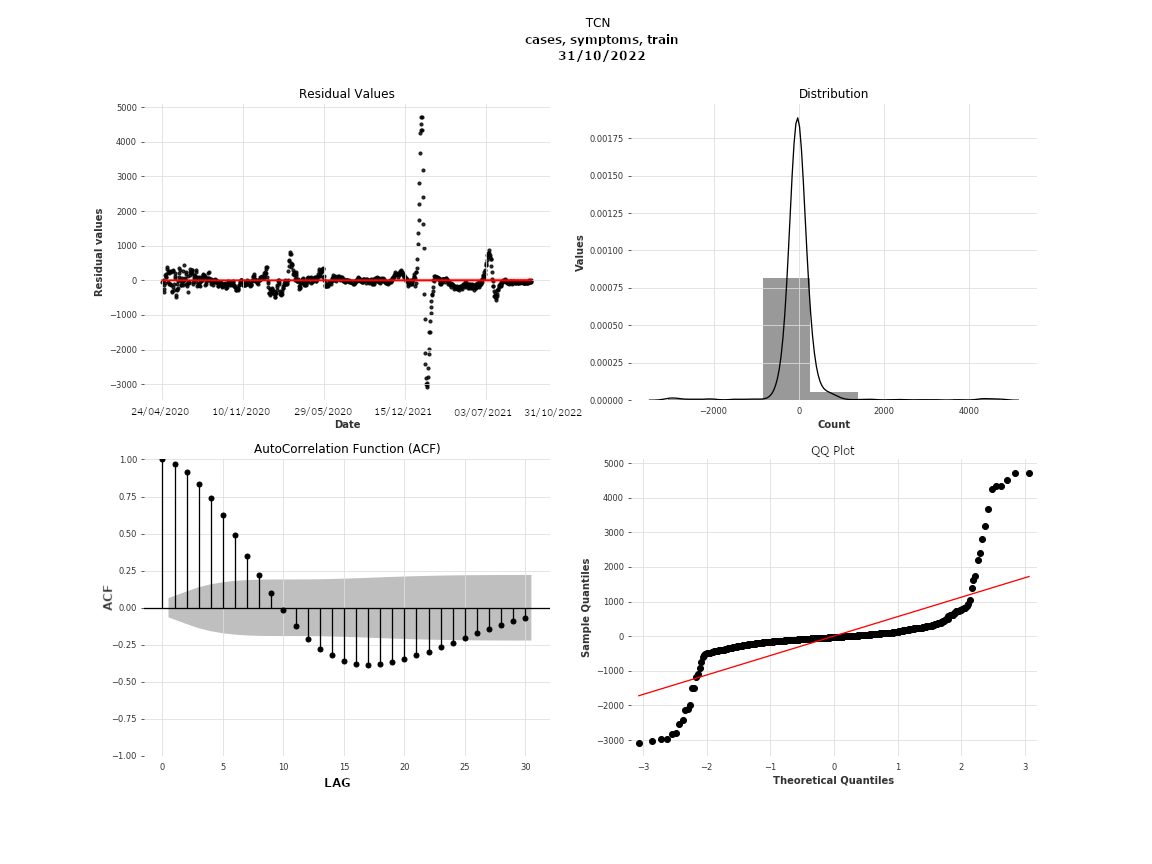
**Fig S2.3.** Graphics of the residual analysis of the TCN model, for the projection on the training data, of the Cases series by date of the beginning of the Symptoms generated on 31-10-2022.


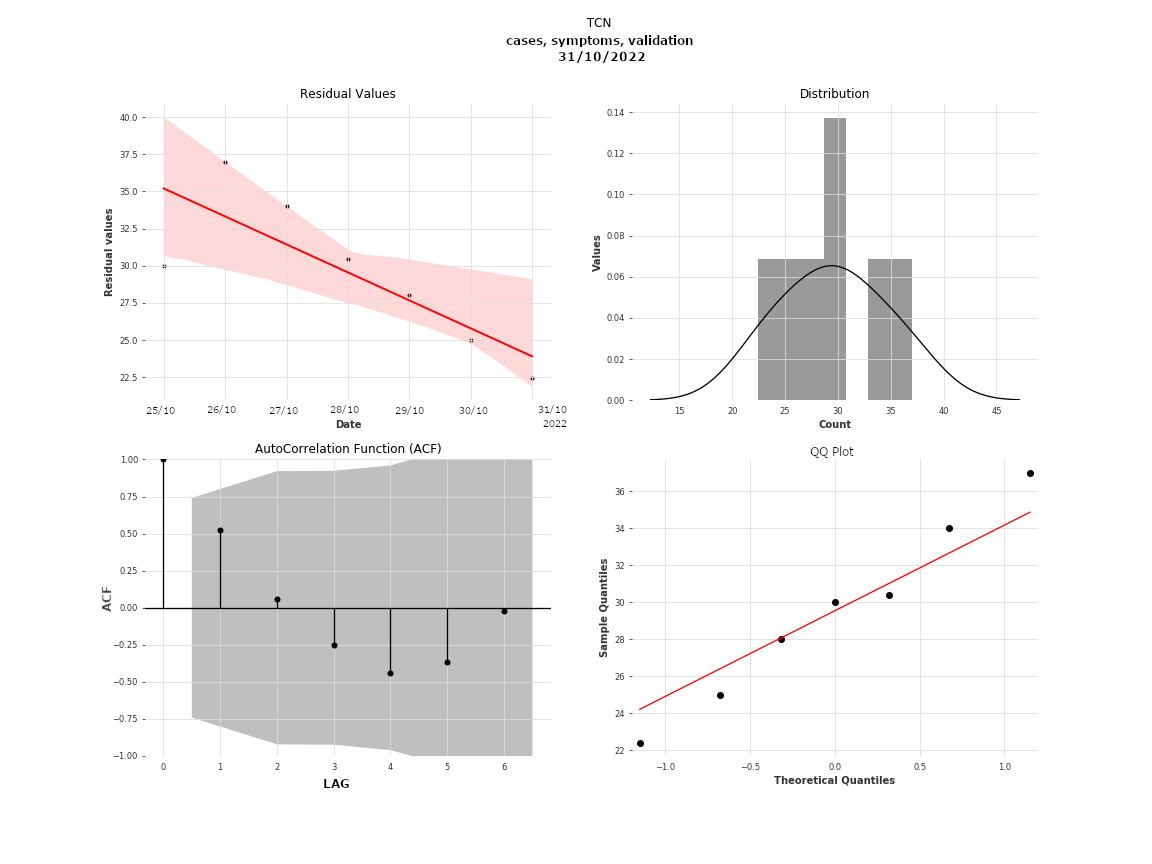


**Fig S2.4**. Graphics of the residual analysis of the TCN model, for the projection on the validation data, of the Cases series by date of the beginning of the Symptoms generated on 31-10-2022.

**
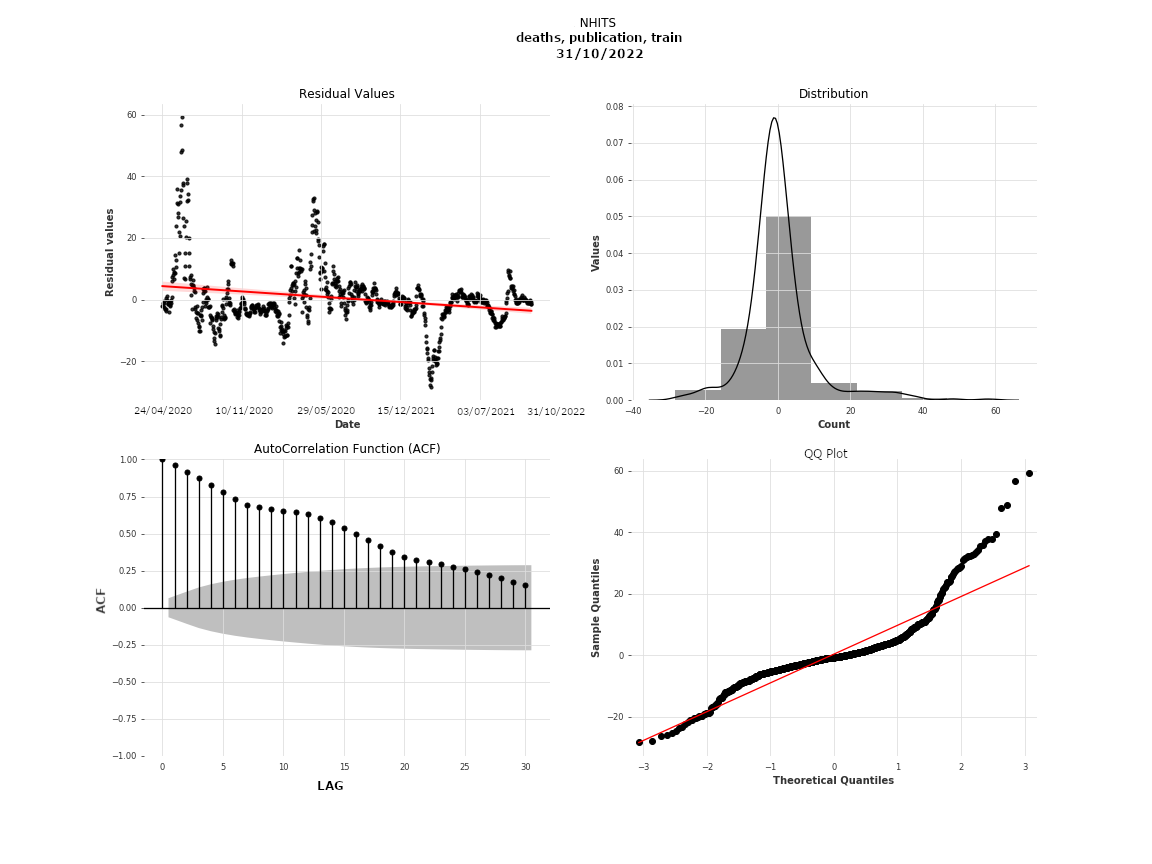
**

**Fig S2.5**. Graphics of the residual analysis of the NHITS model, for the projection on the training data, of the Publication of Deaths series generated on 31-10-2022.

**
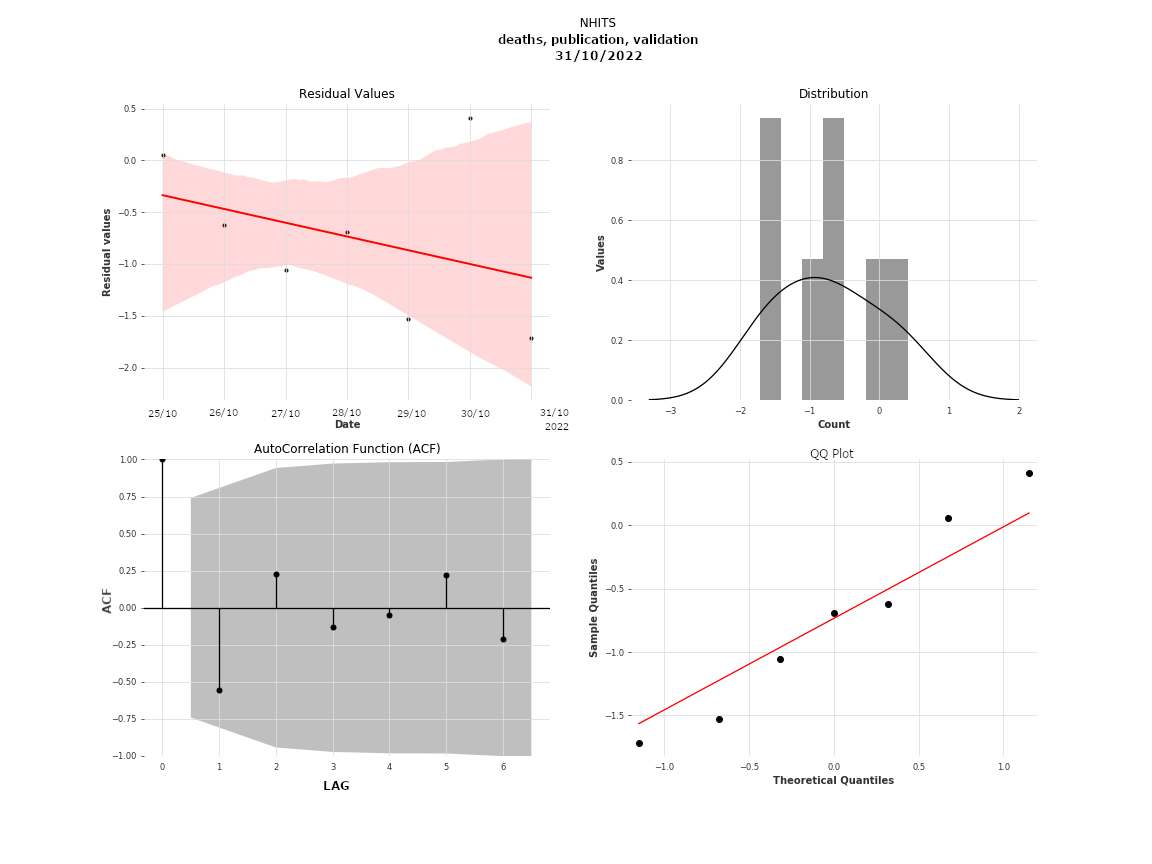
**

**Fig S2.6**. Graphics of the residual analysis of the NHITS model, for the projection on the validation data, of the Publication of Deaths series generated on 31-10-2022.


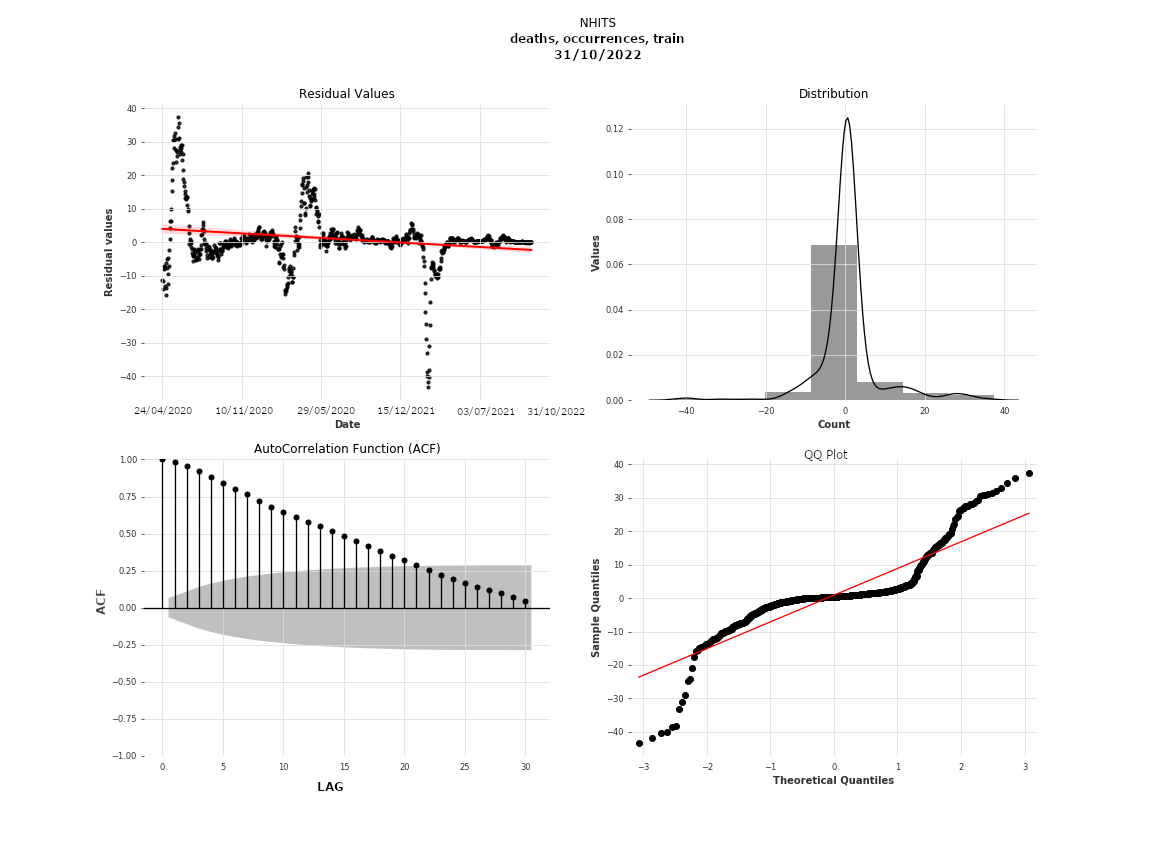
**Fig S2.7**. Graphics of the residual analysis of the NHITS model, for the projection on the training data, of the Occurrence of Deaths series generated on 31-10-2022.


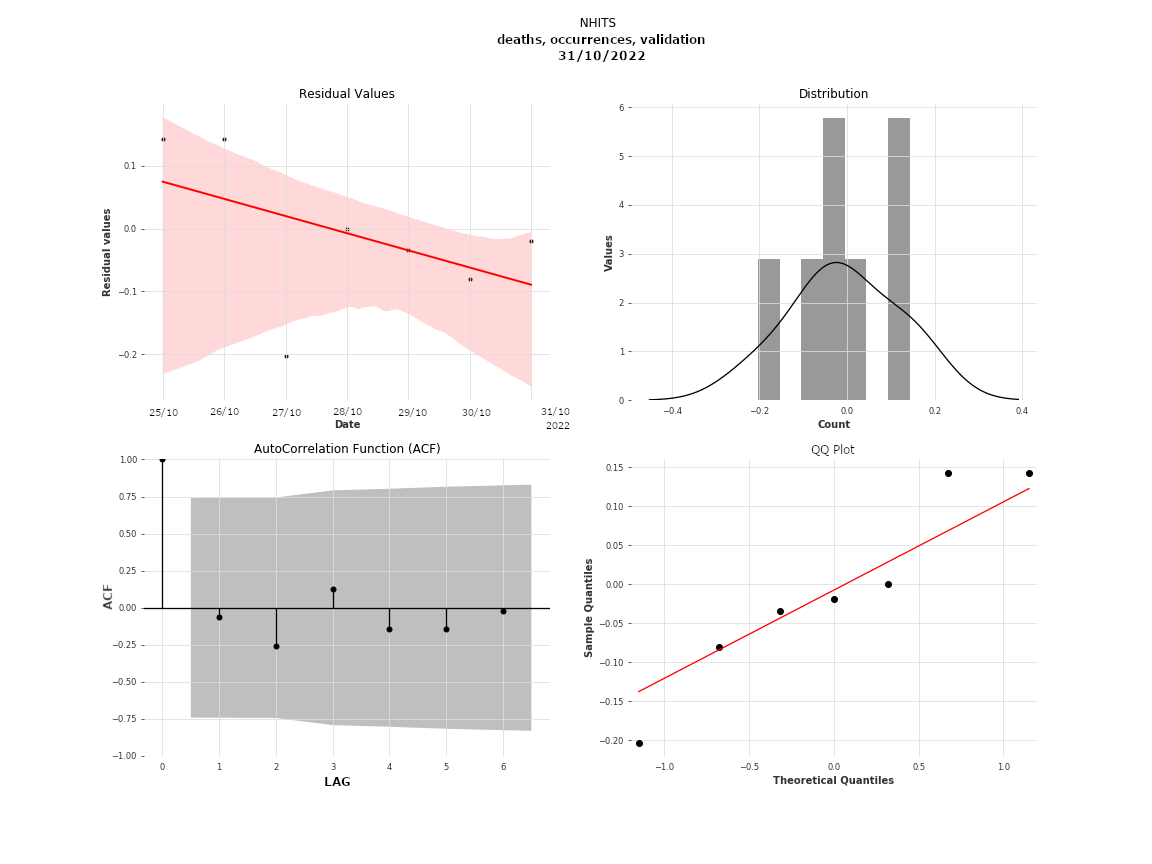


**Fig S2.8**. Graphics of the residual analysis of the NHITS model, for the projection on the validation data, of the Occurrence of Deaths series generated on 31-10-2022.
